# Comparison of the relationship between key demographic features and physical activity levels across 22 countries

**DOI:** 10.1186/s12889-025-23594-3

**Published:** 2025-07-12

**Authors:** Chung Gun Lee, Elizabeth Kwon, Jason Paltzer, Chukwuemeka N. Okafor, Tyler J. VanderWeele, Byron R. Johnson, Joo Hee Choi

**Affiliations:** 1 Department of Physical Education, College of Education 71-1, Seoul National University, Seoul, 08826 South Korea; 2 Institute of Sport Science, Seoul National University, 1 Gwanak-ro, Gwanak-gu, Seoul, 08826 South Korea; 3 Department of Public Health, Baylor University, Waco, TX USA; 4 The Meros Center, Wisconsin Lutheran College, Milwaukee, WI USA; 5 Department of Medicine, Division of Infectious Diseases, Long School of Medicine, University of Texas Health Science Center San Antonio, Antonio, TX USA; 6 Departments of Epidemiology and Biostatistics, Harvard T.H. Chan School of Public Health, Boston, MA USA; 7 Human Flourishing Program, Harvard University, Cambridge, MA USA; 8 Institute for Studies of Religion & Institute for Global Human Flourishing, Baylor University, Waco, TX USA

**Keywords:** Physical activity behavior, Demographic factors, Country differences, Meta-analysis

## Abstract

**Background:**

The present study aims to examine global trends in physical activity levels and explore the potential influence of demographic factors on physical activity participation.

**Methods:**

Using data from the Global Flourishing Study (GFS), which includes 202,898 participants from 22 geographically and culturally diverse countries, we assessed the average number of days of physical activity across various demographic groups (age, gender, marital status, employment, religious service attendance, education, immigration status) and across different countries.

**Results:**

While patterns varied across the 22 countries, certain consistent trends overall emerged. Physical activity levels were higher among individuals aged 60–69 compared to younger and older age groups, men compared to women, and those in domestic partnerships compared to single individuals. Higher activity levels were also observed among the self-employed, those with higher educational attainment, frequent religious service attendees, and individuals born in the country of residence compared to immigrants. Notably, Egypt reported the lowest average number of physical activity days, whereas the Philippines reported the highest.

**Conclusions:**

These results contribute to a deeper understanding of the sociodemographic disparities in physical activity participation. By documenting physical activity patterns across key demographic groups and countries worldwide, this study provides valuable insights into the social determinants of physical activity behavior.

**Supplementary Information:**

The online version contains supplementary material available at 10.1186/s12889-025-23594-3.

## Introduction

Overwhelming evidence has established that physical activity is associated with myriad health benefits. physical activity reduces mortality rates by 13% [1] improves quality of life for patients with chronic diseases [2] and lowers risk of chronic diseases including hypertension [3], diabetes [4], cancer [5], and cardiovascular diseases [6]. Further, physical activity has positive effects on mental health. Physical activity lowers risk of dementia [7] and enhances cognitive functioning [8, 9].

Promoting physical activity is of importance as the association between physical activity and health outcomes follows a dose-response pattern. For example, compared to insufficiently active individuals (less than 600 metabolic equivalent (MET) minutes), those who are low active (600–3999 MET minutes), moderately active (4000–7999 MET minutes), and highly active (≥ 8000 MET minutes) evinced reduction in risk of diabetes by 14%, 25%, and 28%, respectively [6]. In the same study, corresponding risk reductions for ischemic heart disease were 6%, 23%, and 25% [6].

Despite the established health benefits of physical activity, age-standardized prevalence of insufficient physical activity remains high (27.5%) globally [10]. Several demographic factors are known to be associated with physical inactivity. Prevalence of physical inactivity in high-income countries was more than double (36.8%) compared to low-income countries (16.2%) in 2016 [10]. Women (33.9%) were more likely to be inactive than men (27.9%) [11]. Although physical activity levels typically decrease with age, heterogenous patterns were observed in different regions. Older adults (60 + years) in South-East Asia were more active compared to their counterparts in all other regions, and they were more active than young adults (15–29 years) from the Americas, Eastern Mediterranean, and Western Pacific regions [11]. However, existing data are outdated and scarce to inform demographic features that determine physical activity levels.

Although numerous studies have examined the relationship between demographic characteristics and physical activity, existing research is often limited in several ways. Many studies have been conducted within single-country contexts, such as in the United States or European countries [12, 13], making it difficult to generalize findings across culturally and economically diverse populations. For example, prior studies have consistently shown that women and older adults are less likely to meet recommended levels of physical activity [14], and both employment status and education level have also been identified as relevant predictors [15, 16]. However, these findings are typically based on single nation samples and lack a cross-national comparative perspective. Furthermore, few studies have examined multiple demographic variables simultaneously within large international datasets. Most research tends to focus on one or two predictors, such as age or gender [17], without considering how these variables might interact or operate differently across countries with varying social, religious, or economic structures. Additionally, some influential factors such as religious service attendance or immigration status are often overlooked, despite their potential relevance to lifestyle behaviors. While a few studies have examined interventions based on religious beliefs, religiosity, or spirituality, findings suggest that such approaches may have positive effects on increasing physical activity or reducing sedentary behavior [18]; however, the evidence remains limited.

Given these limitations, there remains a need for a comprehensive, multi-country analysis that systematically investigates how a wide range of demographic factors relate to physical activity levels.

Monitoring world-wide trends in physical activity levels is essential to lay a groundwork for national or international intervention and program development. Thus, the current study was conducted to better understand trends in physical activity levels globally and investigate the potential role of demographic factors in determining physical activity levels. Specifically, we investigated key demographic features (age, gender, marital status, employment, religious service attendance, education, immigration status) across 22 countries in our sample; mean levels of physical activity across countries; and how demographic features are associated with physical activity levels globally.

## Methods

The description of the methods below has been adapted from Crabtree et al. (2024). Further methodological detail is available elsewhere [19–25].

### Data

The Global Flourishing Study (GFS) is a study of 202,898 participants from 22 geographically and culturally diverse countries, with nationally representative sampling within each country, concerning the distribution of determinants of well-being. Wave 1 of the data included the following countries and territories: Argentina, Australia, Brazil, Egypt, Germany, Hong Kong (Special Administrative Region of China, with mainland China included from 2024 onwards), India, Indonesia, Israel, Japan, Kenya, Mexico, Nigeria, the Philippines, Poland, South Africa, Spain, Sweden, Tanzania, Türkiye, United Kingdom, and the United States. The countries were selected to (a) maximize coverage of the world’s population, (b) ensure geographic, cultural, and religious diversity, and (c) prioritize feasibility and existing data collection infrastructure. Data collection was carried out by Gallup Inc. Data for Wave 1 were collected principally during 2023, with some countries beginning data collection in 2022 and exact dates varying by country [24]. Four additional waves of panel data on the participants will be collected annually from 2024 to 2027. The precise sampling design to ensure nationally representative samples varied by country and further details are available in [24]. Survey items included aspects of well-being such as happiness, health, meaning, character, relationships, and financial stability [26], along with other demographic, social, economic, political, religious, personality, childhood, community, health, and well-being variables. The data are publicly available through the Center for Open Science (https://www.cos.io/gfs). During the translation process, Gallup adhered to TRAPD model (translation, review, adjudication, pretesting, and documentation) for cross-cultural survey research (ccsg.isr.umich.edu/chapters/translation/overview).

### Measures

#### Demographics variables

Continuous age was classified as 18–24, 25–29, 30–39, 40–49, 50–59, 60–69, 70–79, and 80 or older. Gender was assessed as male, female, or other. Marital status was assessed as single/never married, married, separated, divorced, widowed, and domestic partner. Employment was assessed as employed, self-employed, retired, student, homemaker, unemployed and searching, and other. Education was assessed as up to 8 years, 9–15 years, and 16 + years. Service attendance was assessed as more than once/week, once/week, one-to-three times/month, a few times/years, or never. Immigration status was dichotomously assessed with: “Were you born in this country, or not?” Religious tradition/affiliation with categories of Christianity, Islam, Hinduism, Buddhism, Judaism, Sikhism, Baha’i, Jainism, Shinto, Taoism, Confucianism, Primal/Animist/Folk religion, Spiritism, African-Derived, some other religion, or no religion/atheist/agnostic; precise response categories varied by country [27]. Racial/ethnic identity was assessed in some, but not all, countries, with response categories varying by country. For additional details on the assessments see the COS GFS codebook or [12].

#### Outcome variable

Physical activity in the GFS was assessed with the following questions: “how many days did you exercise or engage in vigorous physical activities for 30 minutes or more in the past week?” Respondents could answer 0 days to 7 days (Every day). This variable was directly coded from 0 days (0) to 7 days (7).

### Analysis

Descriptive statistics for the full sample, weighted to be nationally representative within each country, were estimated for each of the demographic variables. Nationally representative mean days of physical activity were estimated separately for each country and ordered from highest to lowest along with 95% confidence intervals, standard deviations, and Gini coefficients. Variation in means in days of physical activity across demographic categories were estimated, with all analyses initially conducted by country (see supplementary Information). Primary results consisted of a random effects meta-analysis of country-specific mean days of physical activity in each specific demographic category [28, 29] along with 95% confidence intervals, standard errors, upper and upper limits of a 95% prediction interval across countries, heterogeneity (τ), and I2 for evidence concerning variation within a particular demographic variable across countries [30]. Forest plots of estimates are available in the online supplement. All meta-analyses were conducted in R (R Core Team) using the metafor package [31]. Within each country, a global test of variation of outcome across levels of each particular demographic variable was conducted, and a pooled *p*-value [32] across countries reported concerning evidence for variation within any country. Bonferroni corrected *p*-value thresholds are provided based on the number of demographic variables [33, 34]. Religious affiliation/tradition and race/ethnicity were used, when available, as control variables within country, but were not included in the meta-analyses since the availability of these response categories varied by country. As a supplementary analysis, population weighted meta-analyses were also conducted. All analyses were pre-registered with COS prior to data access (https://osf.io/ewyr5/?view_only=1fceb9e7dac440a88ad1d5764a6ea6bd,); all code to reproduce analyses are openly available in an online repository [22].

#### Missing data

Missing data on all variables was imputed using multivariate imputation by chained equations, and five imputed datasets were used [35–38]. To account for variation in the assessment of certain variables across countries (e.g., religious affiliation/tradition and race/ethnicity), the imputation process was conducted separately in each country. This within-country imputation approach ensured that the imputation models accurately reflected country-specific contexts and assessment methods. Sampling weights were included in the imputation model to account for specific-variable missingness that may have been related to probability of inclusion in the study.

#### Accounting for complex sampling design

The GFS used different sampling schemes across countries based on availability of existing panels and recruitment needs [22]. All analyses accounted for the complex survey design components by including weights, primary sampling units, and strata. Additional methodological detail, including accounting for the complex sampling design is provided elsewhere [38].

## Results

Table 1 presents the distribution of individuals across different demographic groups within the observed sample. Most participants were middle-aged falling within the 30–39 years (20%), 40–49 years (17%), or 50–59 years (16%) age groups. Gender distribution was nearly equal, with women comprising 51% and men 49% of the sample. Most of the participants were married (53%) and worked for an employer(39%). Religious attendance exhibited significant variation, with 37% of participants reporting that they never attended, 20% attending a few times a year, and 19% attending once a week. Most respondents (94%) were born in the country where the survey was conducted. Table 1 also provides the distribution of participants by country. The United States (19%) and Japan (10%) accounted for the largest proportions of participants, whereas Turkey (0.7%) and South Africa (1.3%) represented the smallest. Tables S1a to S22a present the variation in number and percentage of individuals within each demographic group across the 22 countries.

**Table 1:** Nationally representative descriptive statistics of the observed sample

Characteristic	*N* = 202,898^a^
**Age group**	
18–24	27,007 (13%)
25–29	20,700 (10%)
30–39	40,256 (20%)
40–49	34,464 (17%)
50–59	31,793 (16%)
60–69	27,763 (14%)
70–79	16,776 (8.1%)
80 or older	4,119 (2.0%)
Missing	20 (< 0.1%)
**Gender**	
Male	98,411 (49%)
Female	103,488 (51%)
Other	602 (< 0.1%)
Missing	397 (< 0.1%)
**Marital status**	
Married	107,354 (53%)
Separated	5,195 (3%)
Divorced	11,654 (6%)
Widowed	9,823 (5%)
Domestic Partner	14,921 (7%)
Single/Never married	52,115 (26%)
Missing	1,826 (1%)
**Employment**	
Employed for an employer	78,815 (39%)
Self-employed	36,362 (18%)
Retired	29,303 (14%)
Student	10,726 (5%)
Homemaker	21,677 (11%)
Unemployed and looking for a job	16,790 (8%)
None of these/other	8,431 (4%)
Missing	793 (0%)
**Religious service attendance**	
> 1/week	26,537 (13%)
1/week	39,157 (19%)
1–3/month	19,749 (10%)
A few times a year	41,436 (20%)
Never	75,297 (37%)
Missing	722 (0%)
**Education**	
up to 8 years	45,078 (22%)
9–15 years	115,097 (57%)
16 + years	42,578 (21%)
Missing	146 (0%)
**Immigration**	
Born in this country	190,998 (94%)
Born in another country	9,791 (5%)
Missing	2,110 (1%)
**Country**	
Argentina	6,724 (3.3%)
Australia	3,844 (1.9%)
Brazil	13,204 (6.5%)
Egypt	4,729 (2.3%)
Germany	9,506 (4.7%)
Hong Kong	3,012 (1.5%)
Indonesia	6,992 (3.4%)
Israel	3,669 (1.8%)
Japan	20,543 (10%)
Kenya	11,389 (5.6%)
Mexico	5,776 (2.8%)
Nigeria	6,827 (3.4%)
Philippines	5,292 (2.6%)
Poland	10,389 (5.1%)
South Africa	2,651 (1.3%)
Spain	6,290 (3.1%)
Sweden	15,068 (7.4%)
Turkey	1,473 (0.7%)
United Kingdom	5,368 (2.6%)
United States	38,312 (19%)

^a^n (%)

Focusing on the primary outcome of this study, Table 2 orders the countries based on the mean days of physical activity. It is important to note that the physical activity frequency was coded on a scale from 0 days (0) to 7 days (7). The countries with the highest mean days of physical activity were Philippines (3.82), Tanzania (3.26), and India (3.12) whereas the countries with the lowest mean days of physical activity were Poland (1.39), Israel (1.29), and Egypt (0.70). In this context, larger standard deviations indicate greater variability. The country with the highest standard deviations is India (3.18), while Israel has the lowest (1.63). Additionally, the Gini coefficient shown in the table reflects the inequality in the distribution of physical activity levels, with values ranging from 0 to 1, where values closer to 1 indicate greater disparity in participation. Among the countries, Egypt exhibits the highest inequality (0.87), suggesting highly uneven physical activity participation across the population, whereas the Philippines shows the lowest (0.41), indicating a more equal distribution of physical activity.

**Table 2:** Ordered means of days of exercise with standard deviations and GINI coefficients

Country	Mean	95% CI	SD	Gini
Philippines	3.82	(3.73, 3.92)	2.84	0.41
Tanzania	3.26	(3.13, 3.39)	2.72	0.47
India	3.12	(3.04, 3.21)	3.18	0.54
Nigeria	3.00	(2.88, 3.12)	2.59	0.49
Indonesia	2.96	(2.85, 3.06)	2.76	0.51
Turkey	2.80	(2.62, 2.99)	2.79	0.55
Sweden	2.77	(2.73, 2.81)	2.26	0.46
Australia	2.68	(2.59, 2.77)	2.27	0.48
Spain	2.68	(2.60, 2.76)	3.18	0.49
Kenya	2.64	(2.54, 2.74)	2.59	0.56
United States	2.58	(2.53, 2.63)	2.76	0.49
United Kingdom	2.55	(2.46, 2.64)	2.37	0.52
Mexico	2.45	(2.36, 2.54)	2.54	0.57
Germany	2.38	(2.32, 2.44)	2.15	0.50
South Africa	2.33	(2.20, 2.46)	2.44	0.57
Hong Kong	2.24	(2.13, 2.35)	2.17	0.53
Brazil	2.16	(2.10, 2.21)	2.43	0.60
Japan	2.04	(2.00, 2.08)	2.45	0.63
Argentina	1.96	(1.88, 2.04)	2.33	0.63
Poland	1.39	(1.30, 1.48)	1.87	0.67
Israel	1.29	(1.18, 1.40)	1.63	0.64
Egypt	0.70	(0.64, 0.76)	1.70	0.87

Table 3 provides the meta-analytic means for each demographic group across the 22 countries. This analysis indicates that, when meta-analytically pooled across countries, physical activity is U-shaped with age from age 18 to 69, and then falls again for age 70+; and is moreover monotonically increasing in both frequency of religious service attendance and in education. Furthermore, this analysis demonstrates that people who were born in the country where the survey was conducted have slightly higher days of physical activity than those who were born in another country.

**Table 3:** Random effects meta-analysis of days of exercise means by demographic category

					Prediction Interval						
Variable	Category	Est	95% CI	SE	LL	UL	Heterogeneity (τ)	I^2		eta-squared (η²)	Global *p*-value
Age group										0.006**	< 0.001**
	18–24	2.56	(2.36,2.76)	0.10	1.39	3.24	0.47	96.1			
	25–29	2.44	(2.17,2.71)	0.14	0.78	3.55	0.64	97.6			
	30–39	2.38	(2.10,2.67)	0.14	0.66	3.79	0.67	98.8			
	40–49	2.39	(2.05,2.72)	0.17	0.45	3.97	0.79	99.0			
	50–59	2.46	(2.09,2.83)	0.19	0.36	4.40	0.88	99.1			
	60–69	2.57	(2.15,2.99)	0.21	0.35	4.59	0.99	99.1			
	70–79	2.40	(2.00,2.79)	0.20	0.10	4.58	0.90	98.5			
	80 or older	2.19	(1.81,2.57)	0.19	0.75	3.52	0.80	92.6			
Gender										0.052	< 0.001**
	Male	2.69	(2.37,3.01)	0.16	0.99	4.06	0.76	99.5			
	Female	2.21	(1.93,2.49)	0.14	0.41	3.58	0.66	99.5			
	Other	2.06	(1.59,2.53)	0.24	0.78	3.51	0.66	75.7			
Marital status										0.007**	< 0.001**
	Married	2.44	(2.12,2.77)	0.16	0.50	4.05	0.76	99.6			
	Separated	2.47	(2.16,2.79)	0.16	0.78	4.01	0.69	92.5			
	Divorced	2.31	(2.01,2.62)	0.16	0.90	4.33	0.69	95.9			
	Widowed	2.26	(1.86,2.66)	0.21	0.21	4.41	0.93	97.4			
	Domestic partner	2.63	(2.28,2.98)	0.18	1.75	4.62	0.77	97.9			
	Single, never married	2.50	(2.28,2.72)	0.11	1.56	3.49	0.52	98.3			
Employment status										0.013*	< 0.001**
	Employed for an employer	2.49	(2.20,2.79)	0.15	0.85	3.86	0.70	99.4			
	Self-employed	2.66	(2.36,2.97)	0.16	0.80	4.22	0.72	98.3			
	Retired	2.48	(2.08,2.88)	0.20	0.41	4.43	0.93	99.0			
	Student	2.54	(2.34,2.75)	0.11	1.45	3.58	0.47	92.5			
	Homemaker	2.09	(1.78,2.41)	0.16	0.30	3.82	0.73	98.2			
	Unemployed and looking for a job	2.28	(1.97,2.59)	0.16	1.01	3.65	0.72	97.4			
	None of these/other	2.22	(1.87,2.56)	0.18	0.08	3.58	0.79	98.1			
Education										0.007**	< 0.001**
	Up to 8 years	2.33	(1.92,2.74)	0.21	0.36	4.37	0.95	99.1			
	9–15 years	2.44	(2.15,2.72)	0.14	1.05	3.71	0.67	99.5			
	16 + years	2.60	(2.34,2.87)	0.13	1.16	4.14	0.62	99.0			
Religious service attendance										0.010*	< 0.001**
	> 1/week	2.65	(2.32,2.98)	0.17	0.86	3.75	0.78	98.1			
	1/week	2.54	(2.22,2.86)	0.16	0.74	4.06	0.76	98.7			
	1–3/month	2.53	(2.23,2.83)	0.15	0.92	3.82	0.70	97.4			
	A few times a year	2.42	(2.17,2.67)	0.13	0.87	3.43	0.60	98.3			
	Never	2.29	(2.03,2.56)	0.14	0.47	3.50	0.62	99.2			
Immigration status										0.006**	0.051
	Born in this country	2.45	(2.16,2.74)	0.15	0.70	3.82	0.69	99.7			
	Born in another country	2.32	(2.02,2.63)	0.16	0.51	3.73	0.68	95.1			

*p <.05; **p <.007 (Bonferroni corrected threshold)

The global *p*-value is significant (< 0.001) across all demographic groups, emphasizing the differences in days of physical activity in at least one country for each group. The “tau” estimate indicates an estimate of how much the mean in that demographic category varies across countries. These findings suggest substantial international variability in the days of physical activity within these demographic groups. Tables S1b-S22b in the Supplementary Information allow us to examine the actual variation in the mean days of physical activity for each demographic group in each country. We found significant differences in mean days of physical activity across gender categories in the whole sample, with males typically having higher days of physical activity than females. However, in Spain, South Africa, and the United States, individuals identifying as Other gender reported a higher days of physical activity than males. Notably, Tanzania, Kenya, and India exhibited the largest gender disparities, with males reporting a higher mean days of physical activity compared to females. On the contrary, the smallest differences were observed in Sweden, Türkiye, and Germany, where the mean values for males and females were nearly equivalent.

These analyses also indicate that the mean days of physical activity is higher among late teens to early twenties in most countries, except for Hong Kong, Nigeria, and Tanzania, where middle-aged groups report higher mean values. Meanwhile, in Brazil, Spain, United States, and the Mexico, the mean values were relatively uniform across all age groups. The variations across countries for each demographic group are further illustrated in Figures S1 to S34 of the Supplementary Information. For marital status, most individuals categorized as widowed showed the lower mean days of physical activity, except Japan, Kenya, Mexico and Sweden, where the widowed group demonstrated higher mean values. In the education variable, individuals with 16 + years of education generally demonstrated higher mean in most countries, while those with up to 8 years of education showed lower mean values. However, the opposite trend was observed in Indonesia, Philippines, and Turkey.

It is important to note that Table 3 does not present mean days of physical activity across religious affiliation categories and race or ethnicity, as these categories vary by country. Since Table 3 presents pooled data across all countries, only the demographic categories with consistent classification across countries were included. Mean days of physical activity across religious affiliation and race or ethnicity can be found in Tables S1b-S22b in the Supplementary Information.

Table S23 in the Supplementary Information provides complementary analyses to those in Table 3. While Table 3 shows a random effects meta-analysis, treating each person equally across the 22 countries, Table S23 presents a population-weighted meta-analysis, accounting for the population sizes of each country. The results of both analyses are generally consistent.

## Discussion

This study is the first to examine a nationally representative dataset from 22 countries (*N* = 202,898) to investigate the prevalence of physical activity behavior across various demographic characteristics and countries. The Gini coefficient, a measure of physical activity distribution inequality, exhibited an inverse correlation with the average number of days of physical activity. Notably, the Philippines, with the lowest Gini coefficient (0.41), reported the highest mean value for days of physical activity (3.82) among the 22 countries. In contrast, Egypt, which had the highest Gini coefficient, reported the lowest mean value for days of physical activity (0.87) among the 22 countries. A decade after the Arab uprisings, the Middle East and North Africa (MENA) region, including countries like Egypt and Israel, continues to exhibit some of the highest levels of socioeconomic inequality globally, which may also have influenced disparities in physical activity [39–41]. Following Egypt and Israel, countries with Gini coefficients exceeding 0.6—such as Poland, Argentina, Japan, and Brazil —also exhibited relatively high levels of inequality. Notably, with the exception of Poland, these countries have been consistently characterized by substantial income and socioeconomic inequality [42–44]. To enhance overall levels of physical activity, countries with significant socioeconomic inequality may prioritize assessing the extent of disparities in physical activity participation. Such an analysis can shed light on the primary factors contributing to these inequalities and guide policy recommendations aimed at reducing physical activity disparities over time. Understanding the nature of these inequalities and identifying their key determinants are essential steps in developing effective policies to address and mitigate these disparities.

We conducted a random-effects meta-analysis to assess the average number of days of physical activity across demographic groups across all 22 countries. Although results varied across the 22 countries, our findings revealed that individuals aged 60 to 69, male, those in domestic partnerships, self-employed individuals, those with higher levels of education, frequent attendees of religious services, and individuals born in the country where the survey was conducted reported the highest mean days of physical activity. These findings align with previous studies that have demonstrated higher levels of physical activity among males [14], individuals with higher educational attainment [15, 16], frequent attendees of religious services [18], and native-born individuals [45].

Evidence indicates that adults from higher socioeconomic backgrounds engage in more frequent physical activity compared to their counterparts from lower socioeconomic backgrounds [15, 16]. This difference may stem from the influence of higher socioeconomic status on the availability of physical activity facilities and opportunities for physical activity or from increased awareness of the benefits of physical activity among persons with high socioeconomic status. A recent review on the correlation between religion and physical activity behavior suggests that religion and spirituality are positively linked to engaging in moderate to vigorous physical activity across diverse populations [18]. Given that regular physical activity offers comparable health benefits related to psychological well-being, social interactions, and physical health, individuals who are physically active may be more inclined to engage in spiritual and religious practices. According to previous studies, lower participation in physical activity among immigrants may be influenced by a range of complex and interrelated factors. While low levels of acculturation—such as limited language proficiency or unfamiliarity with societal norms—are often cited as key barriers, research also highlights additional challenges that immigrants commonly face. These include difficulties in accessing information about available exercise opportunities, limited social support networks, financial constraints, cultural perceptions about exercise and health, and a general lack of familiarity with the host country’s recreational infrastructure and physical activity culture [45]. Taken together, these findings suggest that improving physical activity levels among immigrant populations requires more than simply encouraging individual behavioral change. Instead, a comprehensive and culturally sensitive approach is needed—one that not only supports the acculturation process through efforts like language education and social integration programs, but also addresses broader structural and cultural barriers through tailored strategies that consider the unique experiences and needs of diverse immigrant groups.

This study has several limitations. First, the cross-sectional nature of the data precludes making causal interpretations regarding demographic variations in days of physical activity. For instance, while we found a positive association between religious service attendance and physical activity behavior, this may not imply that more frequent religious service attendance increases physical activity participation; it could also be that more days of physical activity encourage more frequent religious service attendance or both could be an indication of greater leisure time available. Again, this was intended as a purely descriptive analysis and the results of this study do not allow us to determine the causality. Second, despite using a large dataset of 202,898 individuals from 22 countries, our findings should not be generalized to countries not included in our sample. Third, participants may have differences in understanding and interpreting the physical activity behavior item. It remains unclear what specific types of physical activity or vigorous physical activities participants engaged in. Combining questions about types and durations of physical activity within a single item may enhance result quality. Further caution is needed in interpreting cross-national differences as these may also be influenced by different modes of assessment, differing interpretation of response scales, and seasonal effects arising from data being collected in different countries at different times of the year.

Despite these limitations, the study has notable strengths including its substantial sample size, extensive global coverage, and nationally representative samples. Our findings advance understanding of the distribution and sociodemographic disparities in physical activity participation. By documenting physical activity proportions across key demographic groups and countries worldwide, this study provides foundational insights for the emerging literature on the social determinants of physical activity. We trust that these findings will be valuable for policymakers and researchers in public health, social sciences, economics, and behavioral sciences, guiding the development of tailored physical activity interventions. Our findings provide the foundation for utilizing future waves of data from the GFS (2024 through 2027), in order to inform the development of interventions aimed at promoting physical activity behavior among adults worldwide.

### Practical recommendations

To reduce physical activity disparities, countries with high socioeconomic inequality should conduct regular assessments of participation gaps and implement community-based programs in underserved areas. Educational campaigns that promote the benefits of exercise may be particularly effective when targeted toward lower-educated populations. For immigrant communities, culturally tailored interventions that address linguistic, cultural, and structural barriers—such as providing multilingual resources and inclusive fitness programs—can enhance participation. Encouraging partnerships between local governments and religious institutions may further promote physical activity through faith-based initiatives. Finally, workplace wellness programs and flexible scheduling could support physical activity among the self-employed and older adults, helping integrate movement into daily routines.

## Supplementary Information

Supplementary Material 1.

## Data Availability

Data are available for download at the Center for Open Science (COS) website (https://www.cos.io/gfs). All analyses were pre-registered with COS prior to data access (osf.io/gam7u). Code to reproduce the analyses is openly available in the online COS repository.

## Abbreviation

GFS: Global Flourishing Study
