# Supplementary material for: Comparison of the relationship between key demographic features and physical activity levels across 22 countries
Source: BMC Public Health. 2025 Jul 12;25:2440. doi: 10.1186/s12889-025-23594-3 (PMC12255111; doi:10.1186/s12889-025-23594-3)
Supplement: Supplementary file 1 — Supplementary Material 1. [file 12889_2025_23594_MOESM1_ESM.docx]

**Supplementary Online Content**

**Figure S1.** Forest plot for ‘Age group’ – 18-24

**Figure S2.** Forest plot for ‘Age group’ – 25-29

**Figure S3.** Forest plot for ‘Age group’ – 18-24

**Figure S4.** Forest plot for ‘Age group’ – 40-49

**Figure S5.** Forest plot for ‘Age group’ – 50-59

**Figure S6.** Forest plot for ‘Age group’ – 60-69

**Figure S7.** Forest plot for ‘Age group’ – 70-79

**Figure S8.** Forest plot for ‘Age group’ – 80 or older

**Figure S9.** Forest plot for ‘Gender’ – ‘Male’

**Figure S10.** Forest plot for ‘Gender’ – ‘Female’

**Figure S11.** Forest plot for ‘Gender’ – ‘Other’

**Figure S12.** Forest plot for ‘Marital status’ – ‘Married’

**Figure S13.** Forest plot for ‘Marital status’ – ‘Separated’

**Figure S14.** Forest plot for ‘Marital status’ – ‘Divorced’

**Figure S15.** Forest plot for ‘Marital status’ – ‘Widowed’

**Figure S16.** Forest plot for ‘Marital status’ – ‘Domestic partner’

**Figure S17.** Forest plot for ‘Marital status’ – ‘Single, never married’

**Figure S18.** Forest plot for ‘Employment status’– ‘Employed for an employer’

**Figure S19.** Forest plot for ‘Employment status’ – ‘Self-employed’

**Figure S19.** Forest plot for ‘Employment status’ – ‘Self-employed’

**Figure S19.** Forest plot for ‘Employment status’ – ‘Self-employed’

**Figure S20.** Forest plot for ‘Employment status’ – ‘Retired’

**Figure S21.** Forest plot for ‘Employment status’ – ‘Student’

**Figure S22.** Forest plot for ‘Employment status’ – ‘Homemaker’

**Figure S23.** Forest plot for ‘Employment status’ – ‘Unemployed and looking for a job’

**Figure S24.** Forest plot for ‘Employment status’ – ‘None of these/other’

**Figure S25.** Forest plot for ‘Education’ – ‘Up to 8 years’

**Figure S26.** Forest plot for ‘Education’ – ‘9-15 years’

**Figure S27.** Forest plot for ‘Education’ – ‘16+ years’

**Figure S28.** Forest plot for ‘Religious service attendance’ – ‘>1/week’

**Figure S29.** Forest plot for ‘Religious service attendance’ – ‘1/week’

**Figure S30.** Forest plot for ‘Religious service attendance’ – ‘1-3/month’

**Figure S31.** Forest plot for ‘Religious service attendance’ – ‘A few times a year’

**Figure S32.** Forest plot for ‘Religious service attendance’ – ‘Never’

**Figure S33.** Forest plot for ‘Immigration status’ – ‘Born in this country’

**Figure S34.** Forest plot for ‘Immigration status’ – ‘Born in another country’

**Table S1a:** Nationally Representative Descriptive Statistics of the Observed Sample (Argentina)

**Table S1b:** Variations Across Demographic Characteristics (Argentina)

**Table S2a:** Nationally Representative Descriptive Statistics of the Observed Sample (Australia)

**Table S2b:** Variations Across Demographic Characteristics (Australia)

**Table S3a:** Nationally Representative Descriptive Statistics of the Observed Sample (Brazil)

**Table S3b:** Variations Across Demographic Characteristics (Brazil)

**Table S4a:** Nationally Representative Descriptive Statistics of the Observed Sample (Egypt)

**Table S4b:** Variations Across Demographic Characteristics (Egypt)

**Table S5a:** Nationally Representative Descriptive Statistics of the Observed Sample (Germany)

**Table S5b:** Variations Across Demographic Characteristics (Germany)

**Table S6a:** Nationally Representative Descriptive Statistics of the Observed Sample (Hong Kong)

**Table S6b:** Variations Across Demographic Characteristics (Hong Kong)

**Table S7a:** Nationally Representative Descriptive Statistics of the Observed Sample (India)

**Table S7b:** Variations Across Demographic Characteristics (India)

**Table S8a:** Nationally Representative Descriptive Statistics of the Observed Sample (Indonesia)

**Table S8b:** Variations Across Demographic Characteristics (Indonesia)

**Table S9a:** Nationally Representative Descriptive Statistics of the Observed Sample (Israel)

**Table S9b:** Variations Across Demographic Characteristics (Israel)

**Table S10a:** Nationally Representative Descriptive Statistics of the Observed Sample (Japan)

**Table S10b:** Variations Across Demographic Characteristics (Japan)

**Table S11a:** Nationally Representative Descriptive Statistics of the Observed Sample (Kenya)

**Table S11b:** Variations Across Demographic Characteristics (Kenya)

**Table S12a:** Nationally Representative Descriptive Statistics of the Observed Sample (Mexico)

**Table S12b:** Variations Across Demographic Characteristics (Mexico)

**Table S13a:** Nationally Representative Descriptive Statistics of the Observed Sample (Nigeria)

**Table S13b:** Variations Across Demographic Characteristics (Nigeria)

**Table S14a:** Nationally Representative Descriptive Statistics of the Observed Sample (Philippines)

**Table S14b:** Variations Across Demographic Characteristics (Philippines)

**Table S15a:** Nationally Representative Descriptive Statistics of the Observed Sample (Poland)

**Table S15b:** Variations Across Demographic Characteristics (Poland)

**Table S16a:** Nationally Representative Descriptive Statistics of the Observed Sample (South Africa)

**Table S16b:** Variations Across Demographic Characteristics (South Africa)

**Table S17a:** Nationally Representative Descriptive Statistics of the Observed Sample (Spain)

**Table S17b:** Variations Across Demographic Characteristics (Spain)

**Table S18a:** Nationally Representative Descriptive Statistics of the Observed Sample (Sweden)

**Table S18b:** Variations Across Demographic Characteristics (Sweden)

**Table S19a:** Nationally Representative Descriptive Statistics of the Observed Sample (Tanzania)

**Table S19b:** Variations Across Demographic Characteristics (Tanzania)

**Table S20a:** Nationally Representative Descriptive Statistics of the Observed Sample (Turkey)

**Table S20b:** Variations Across Demographic Characteristics (Turkey)

**Table S21a:** Nationally Representative Descriptive Statistics of the Observed Sample (United Kingdom)

**Table S21b:** Variations Across Demographic Characteristics (United Kingdom)

**Table S22a:** Nationally Representative Descriptive Statistics of the Observed Sample (United States)

**Table S22b:** Variations Across Demographic Characteristics (United States)


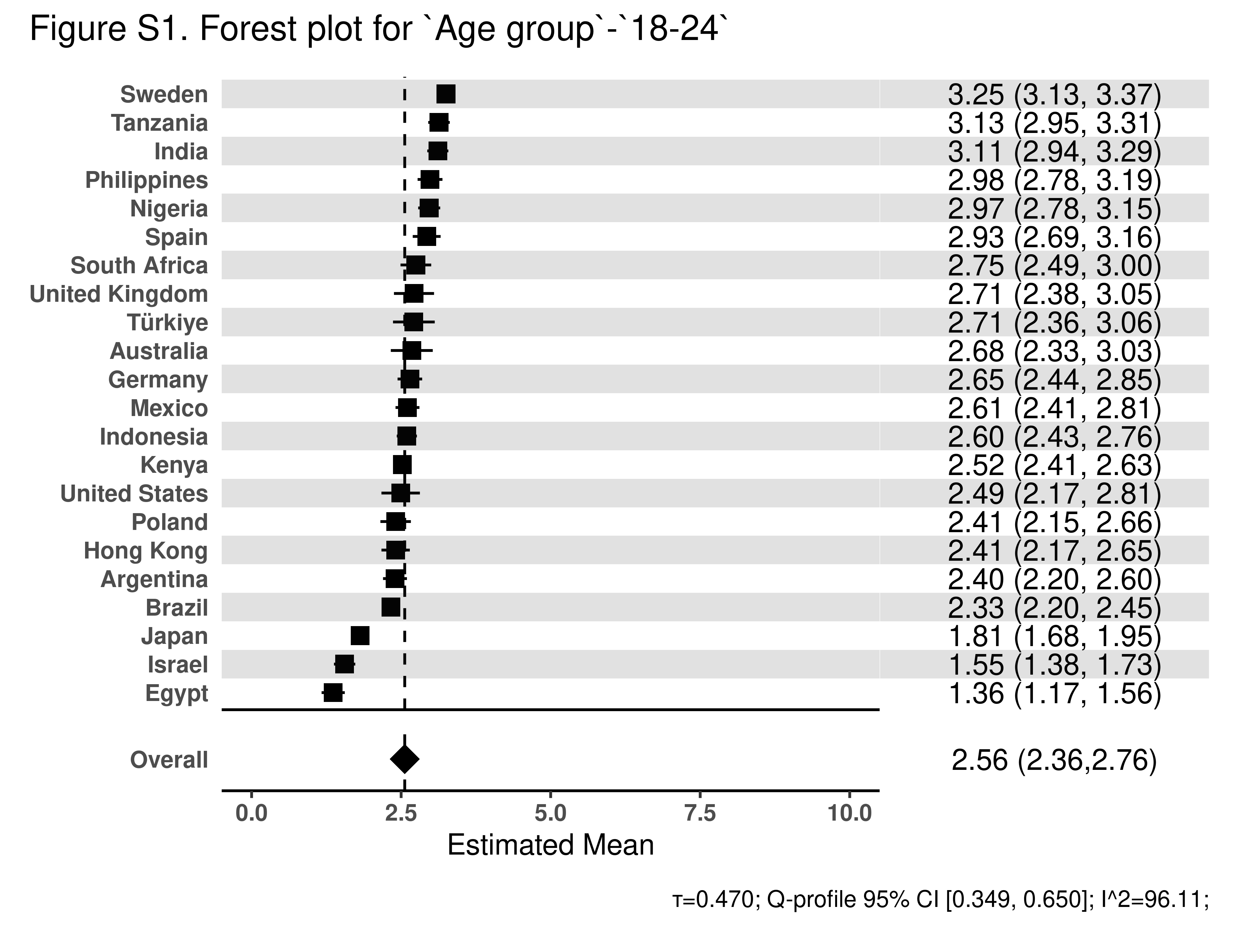

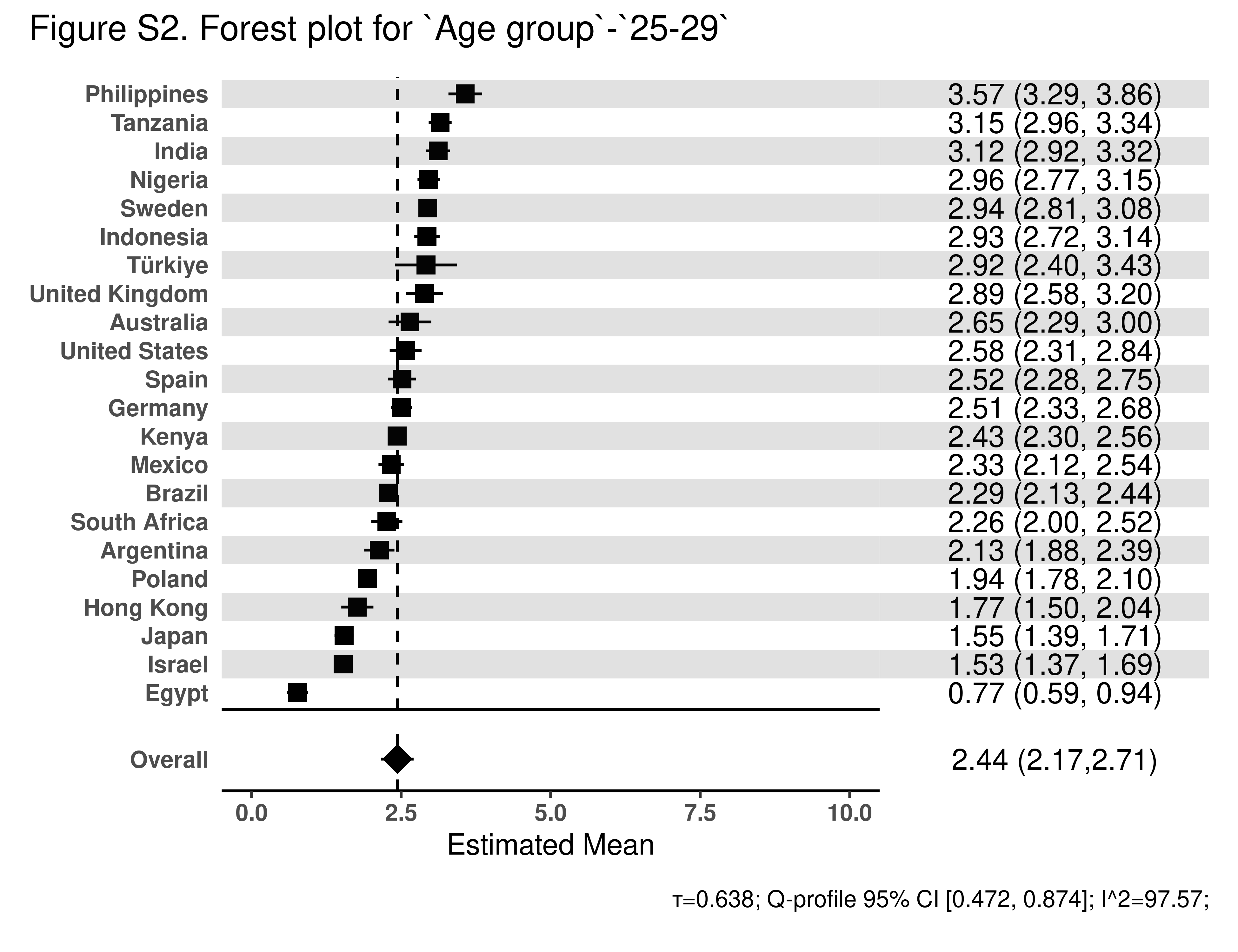

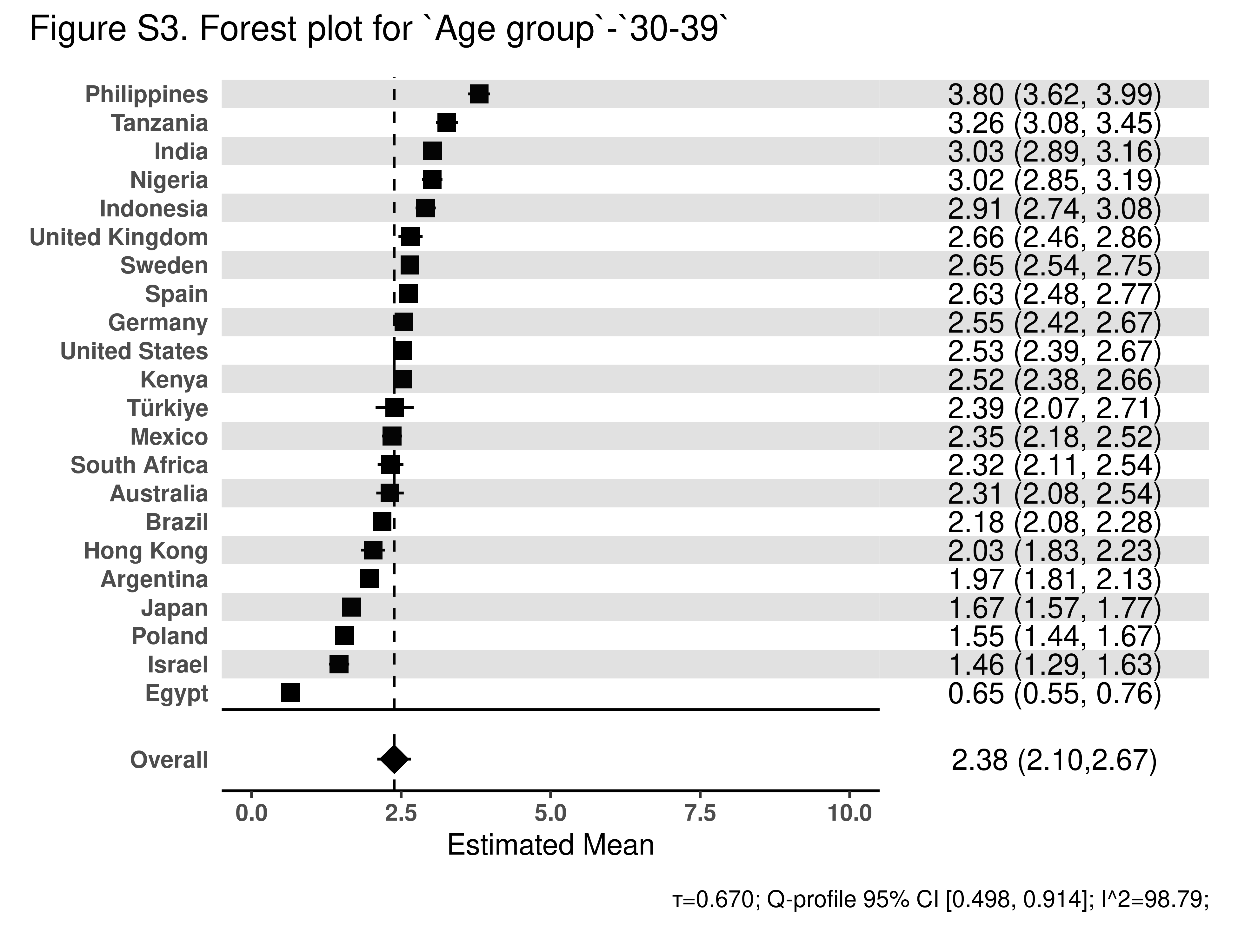

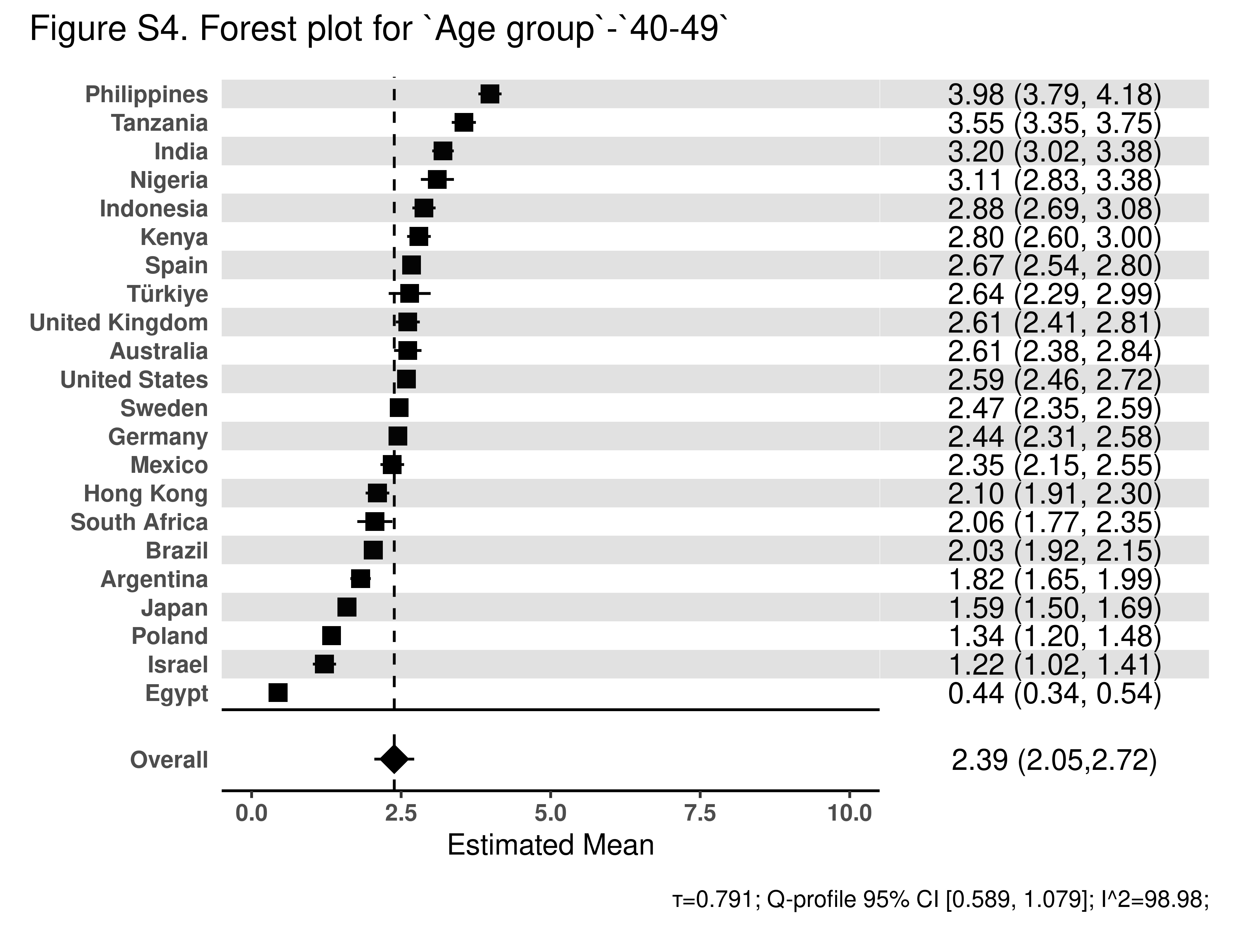

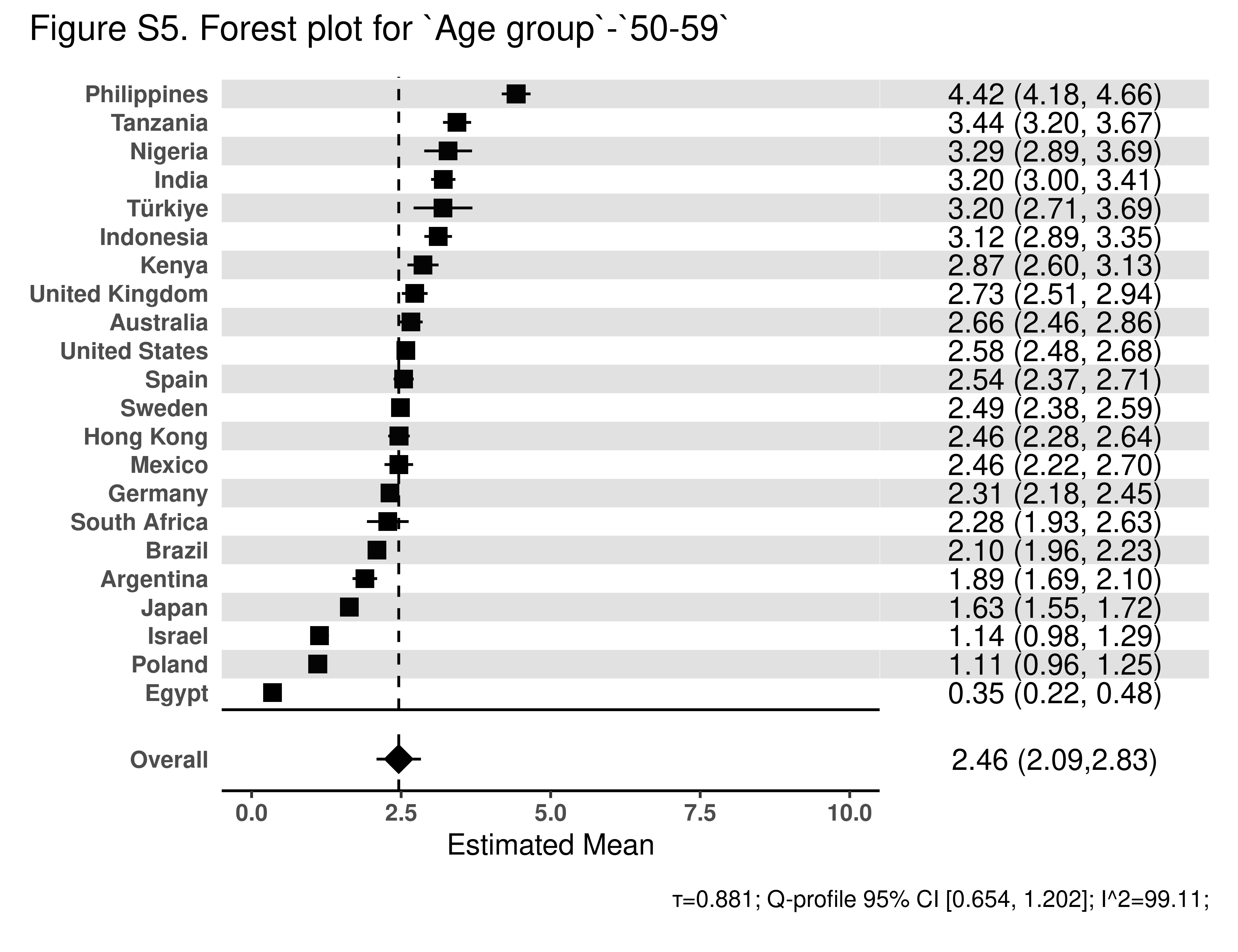

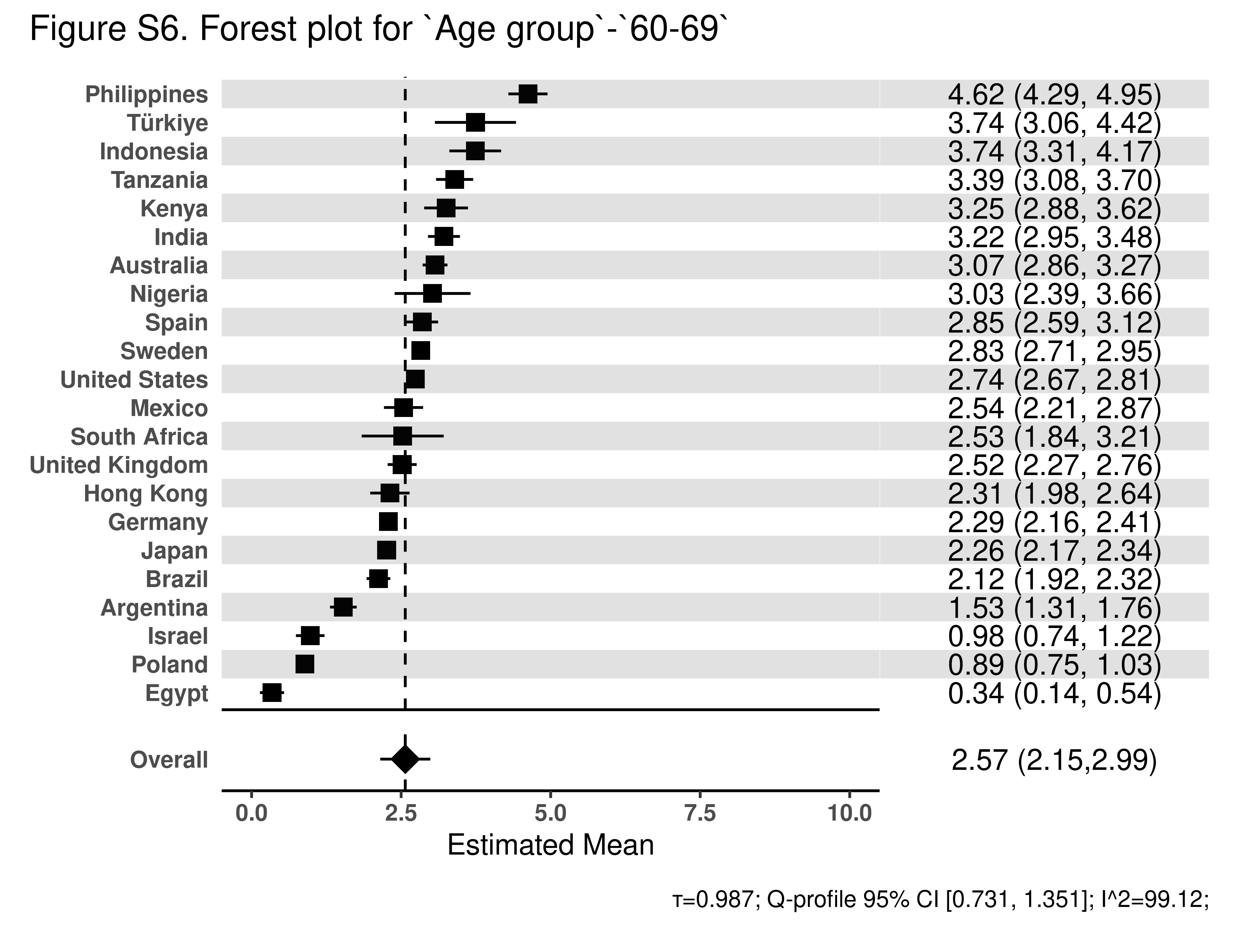

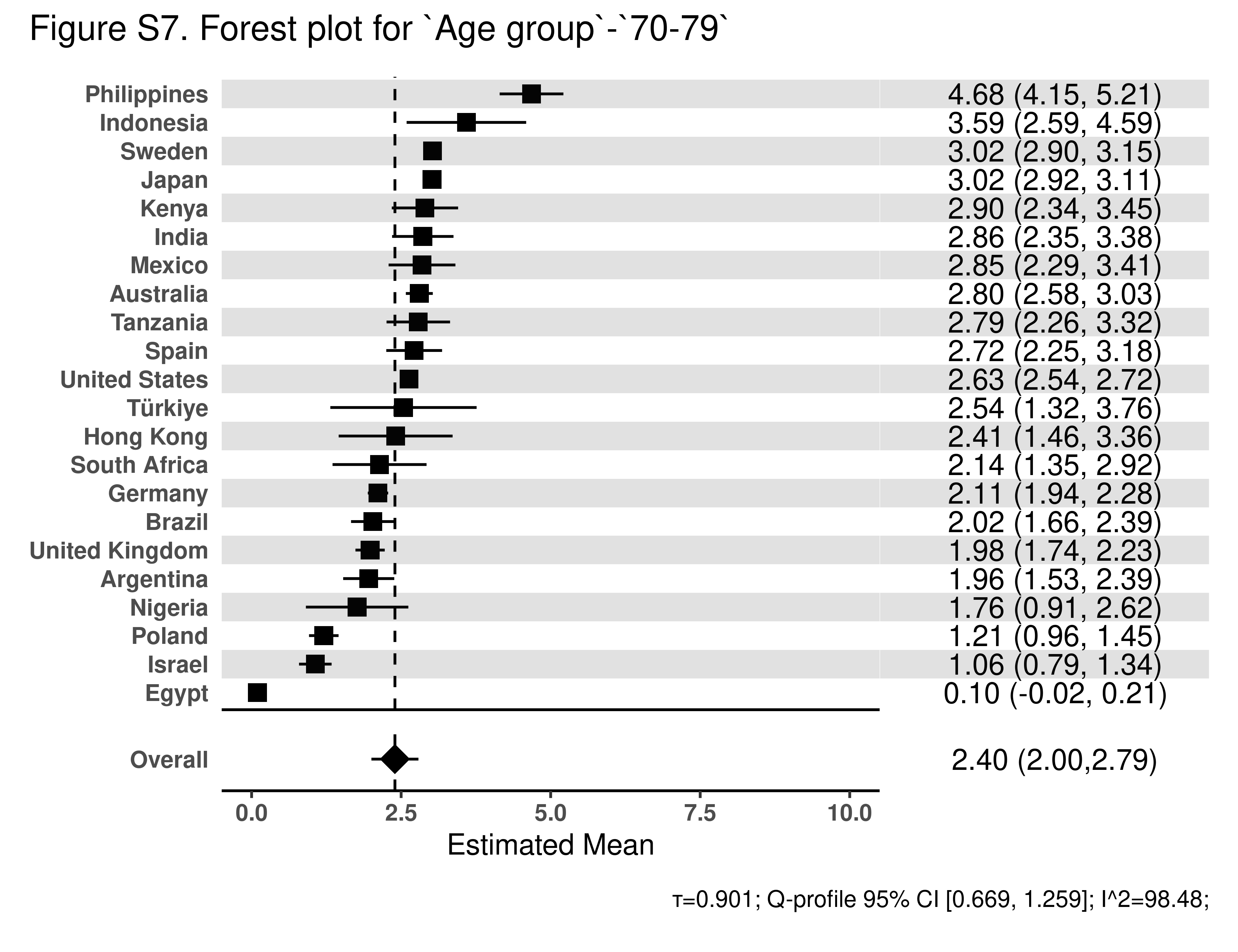

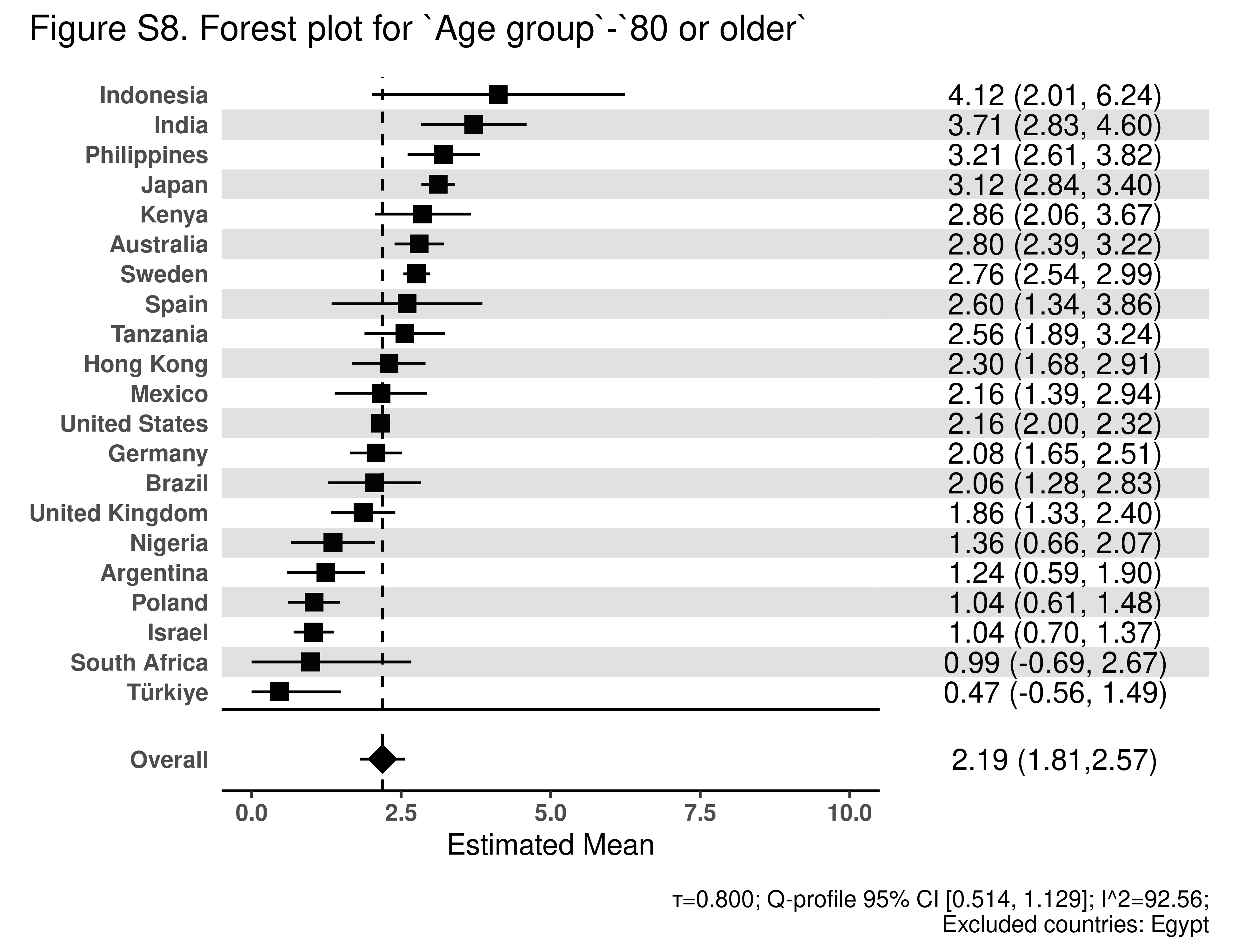

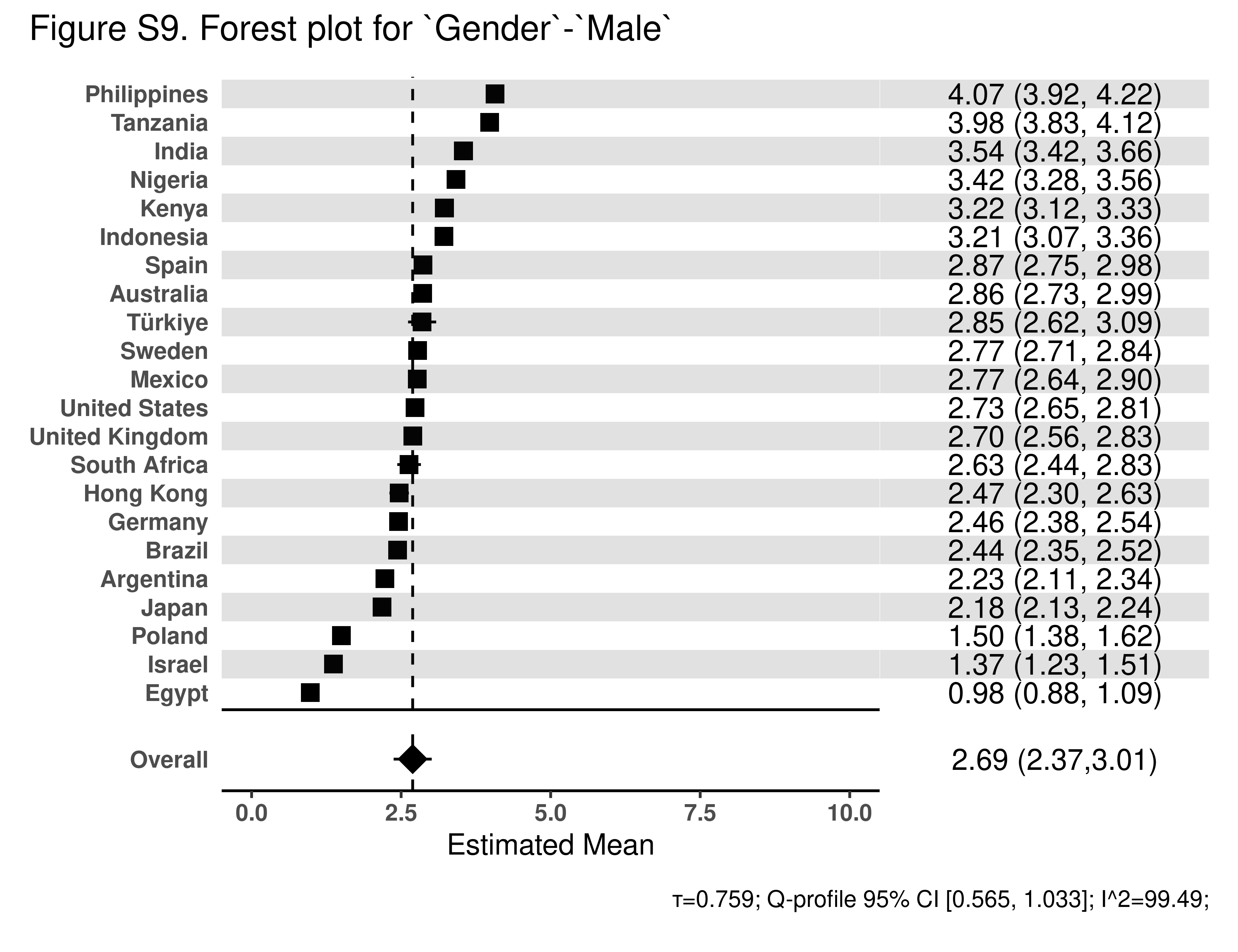

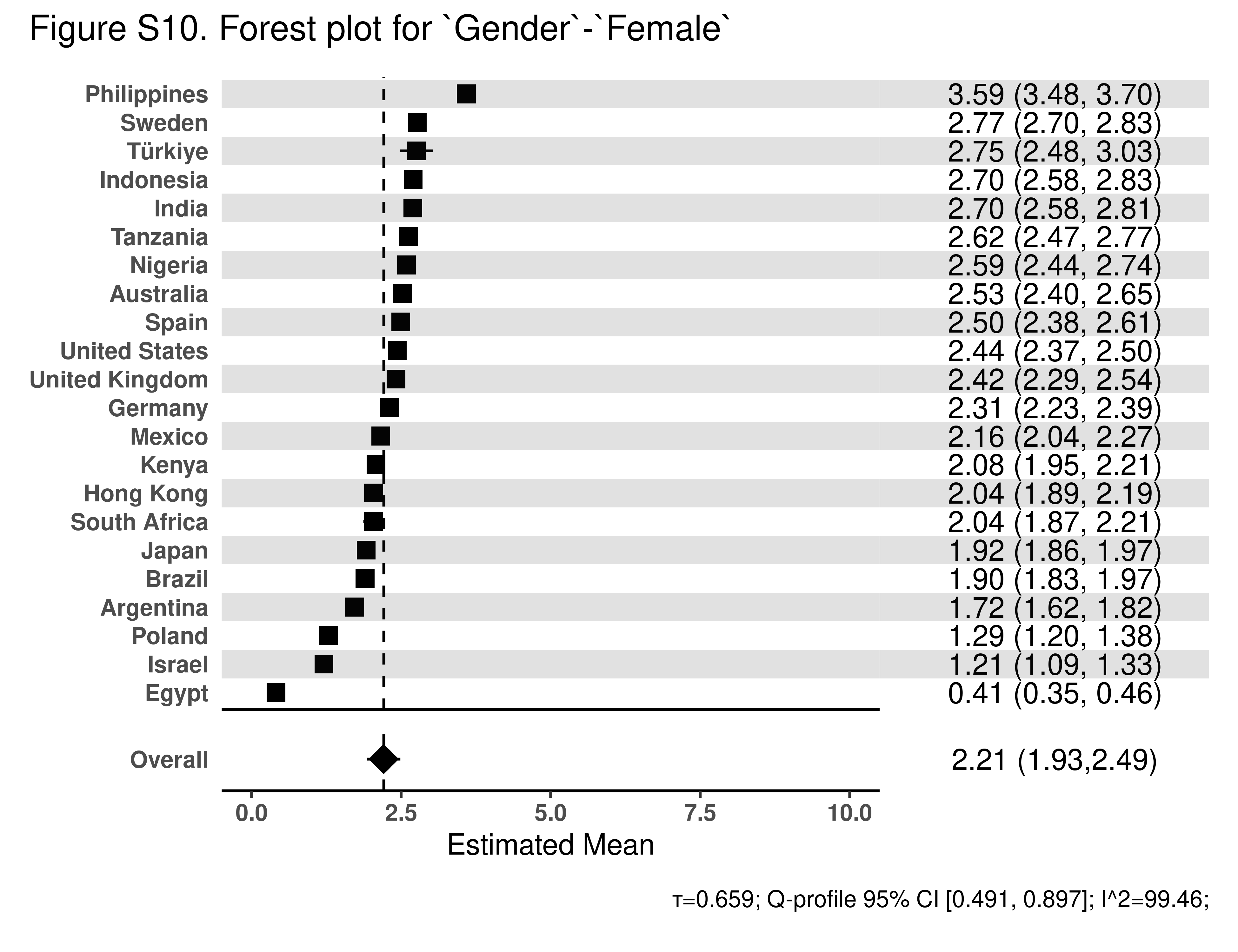

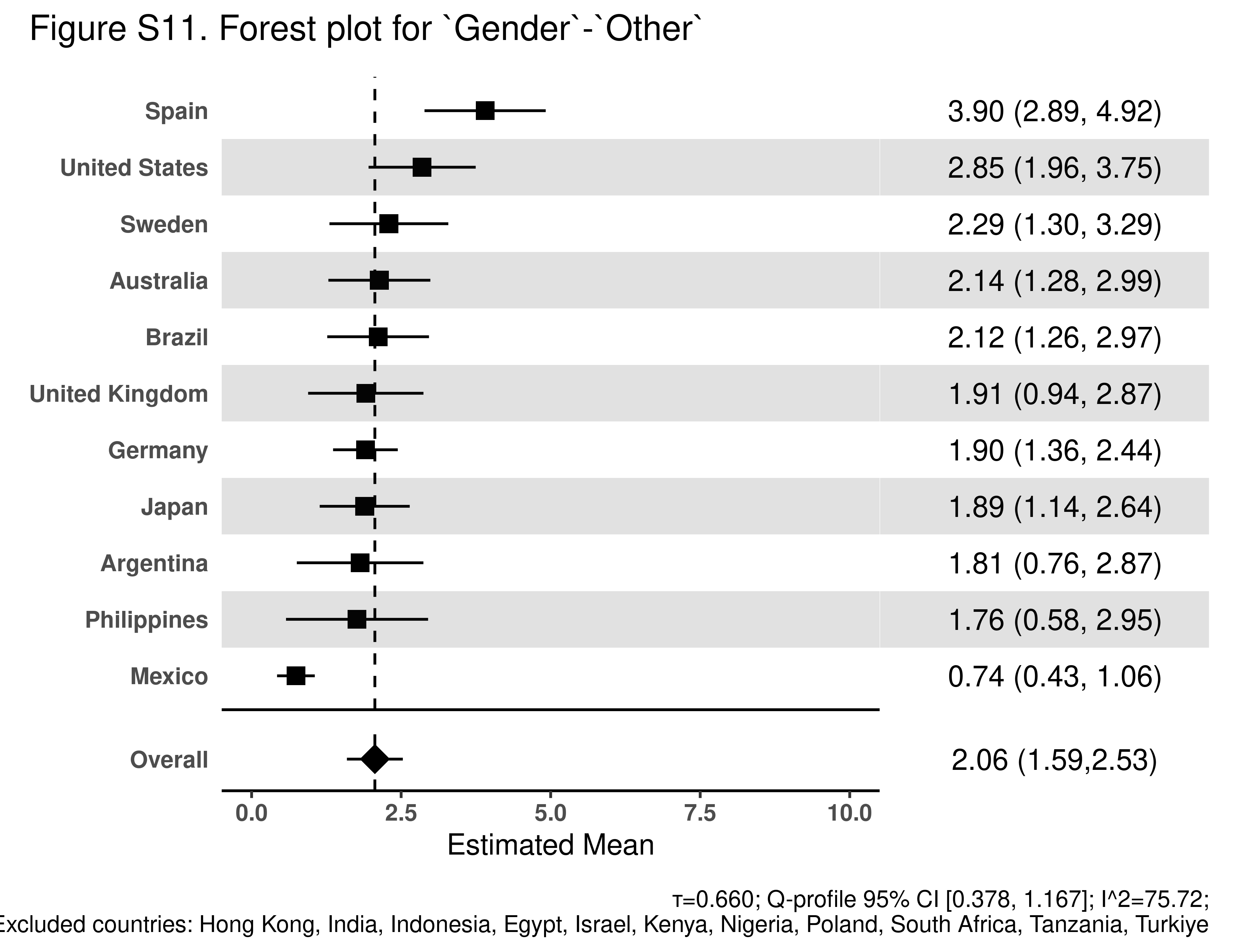

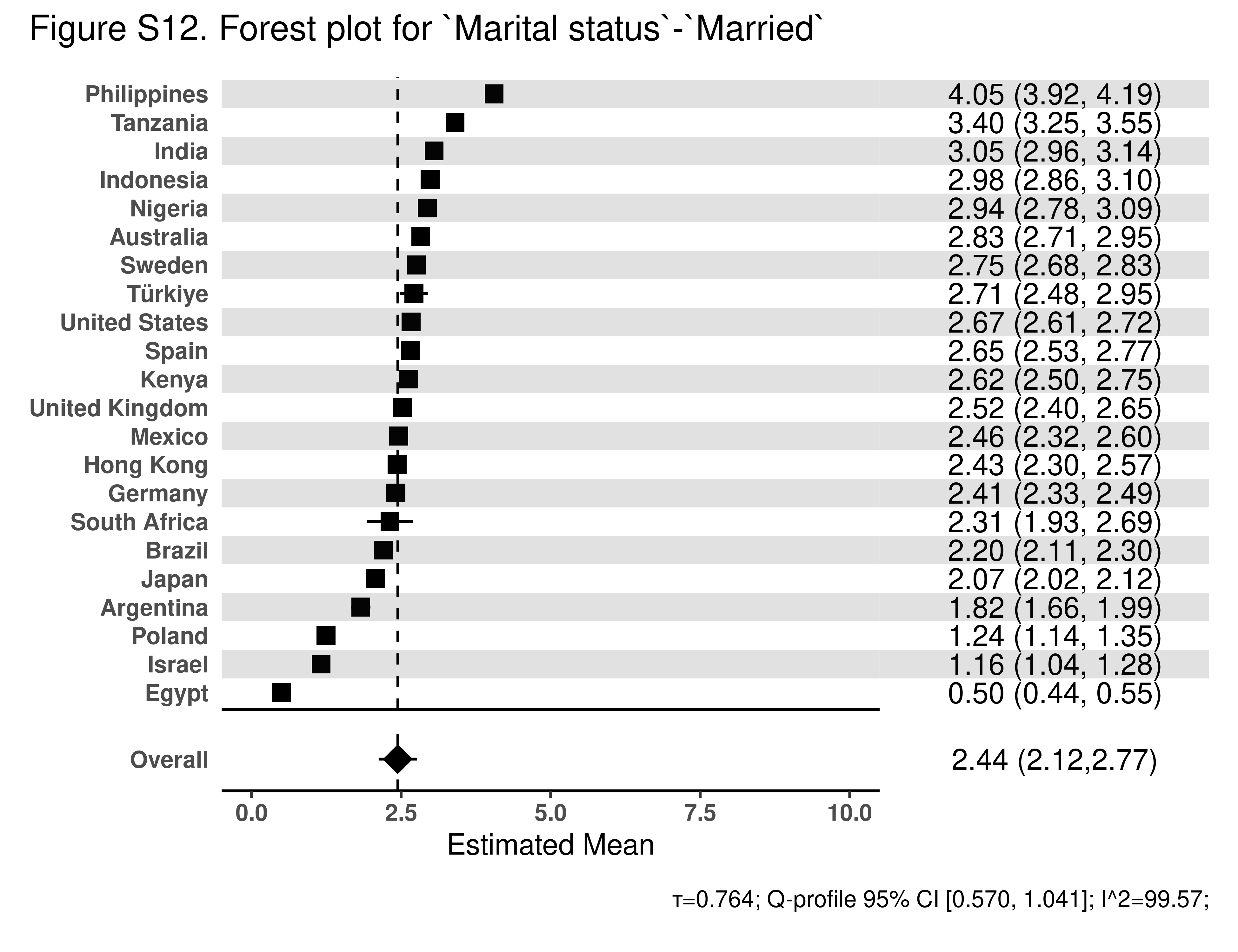

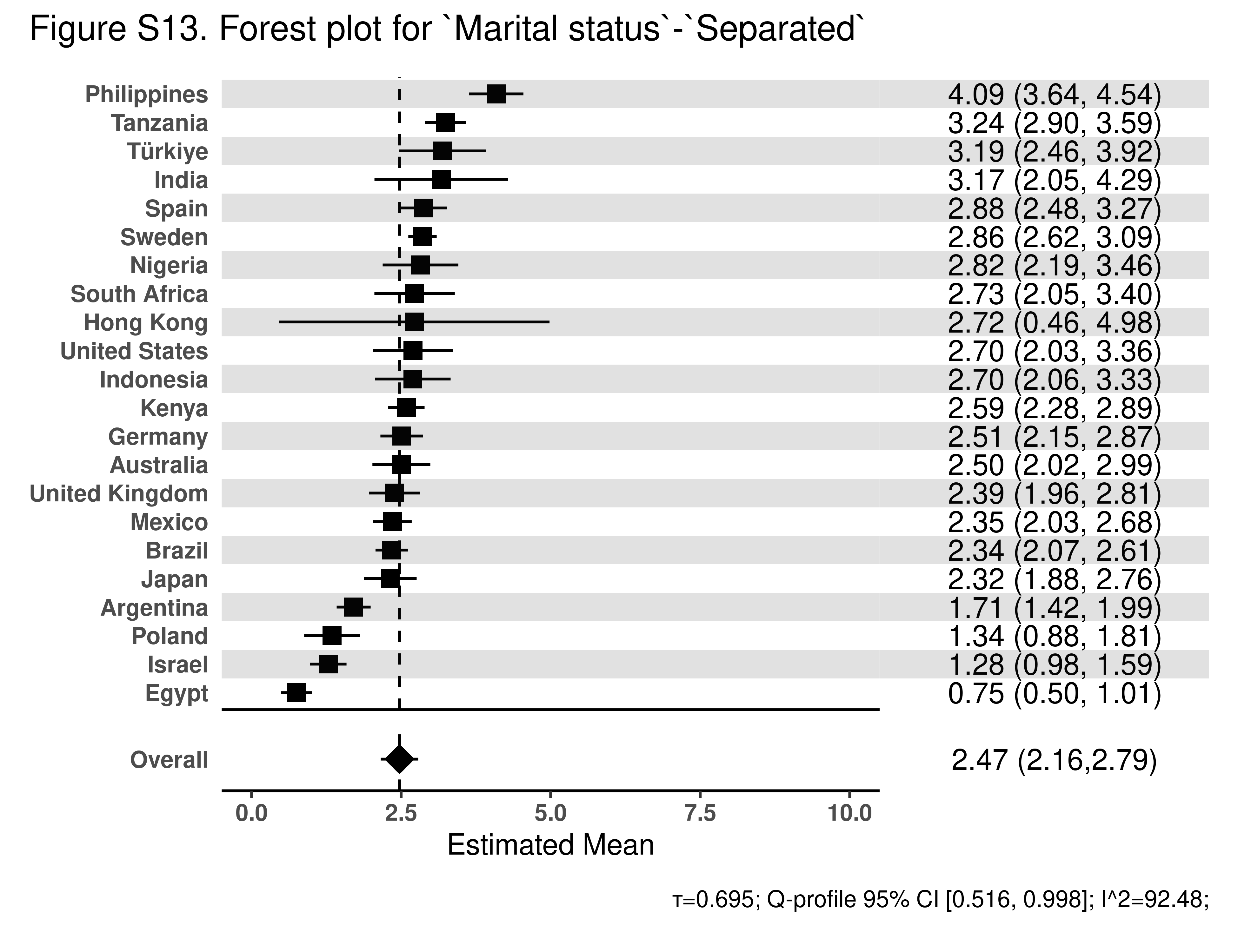

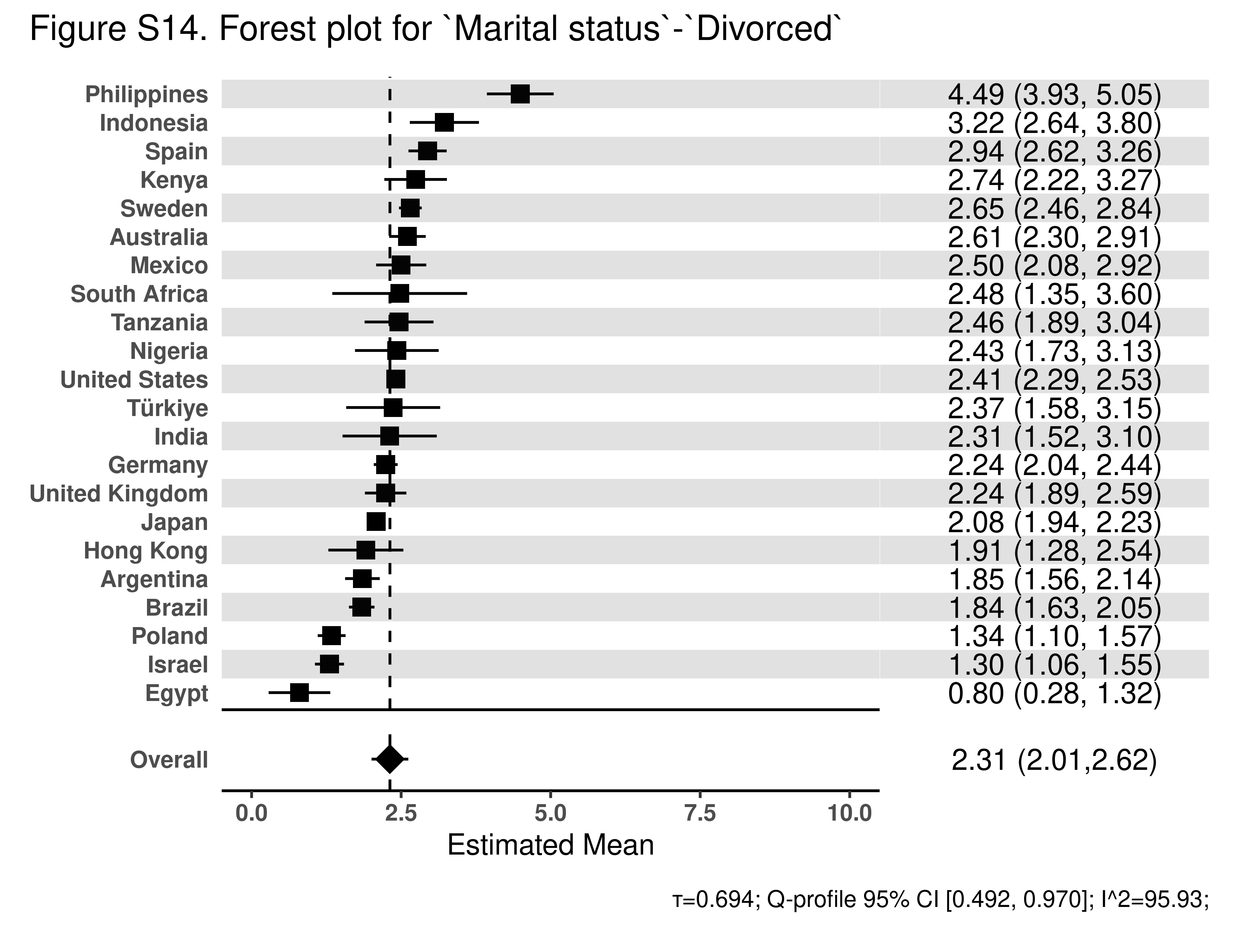

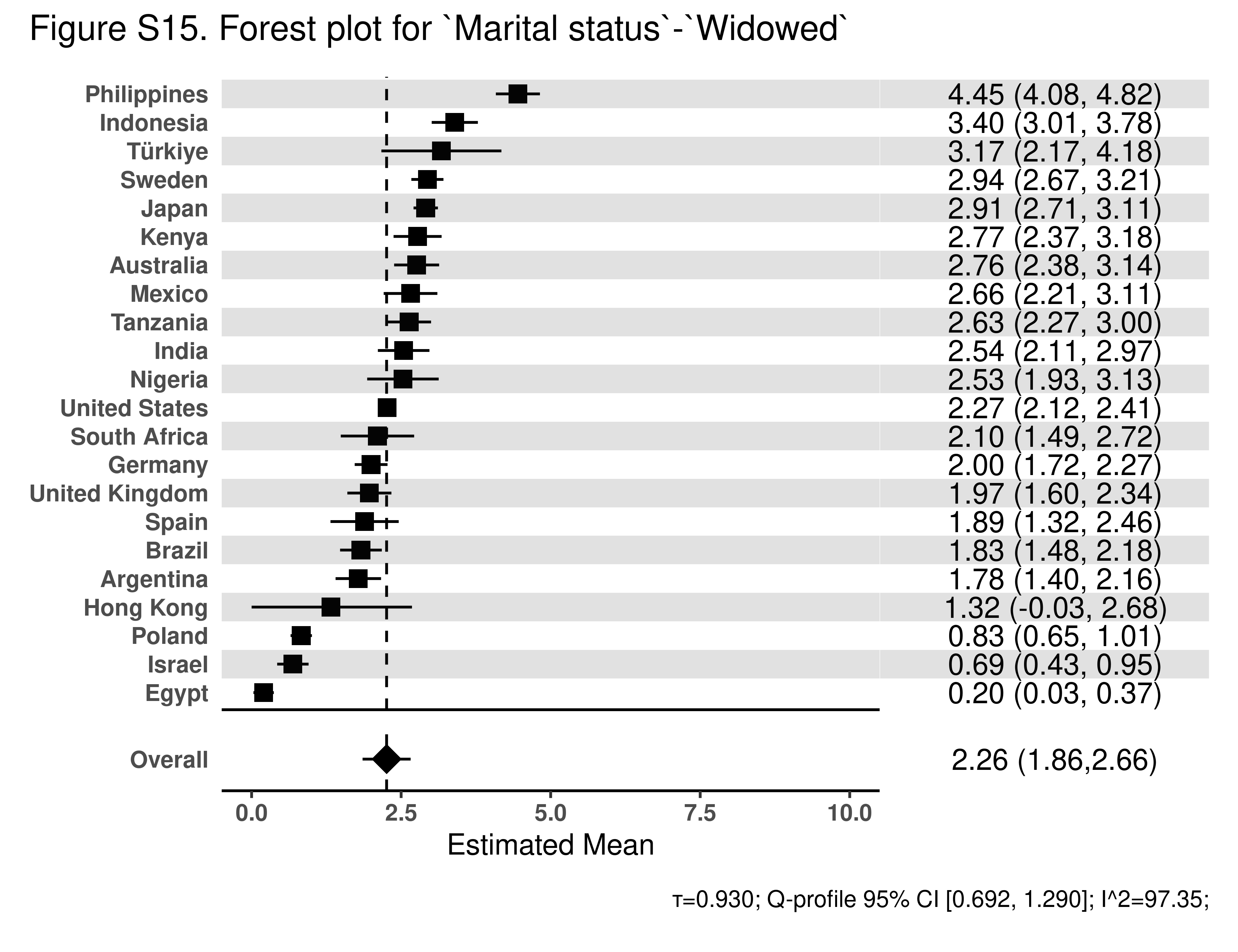

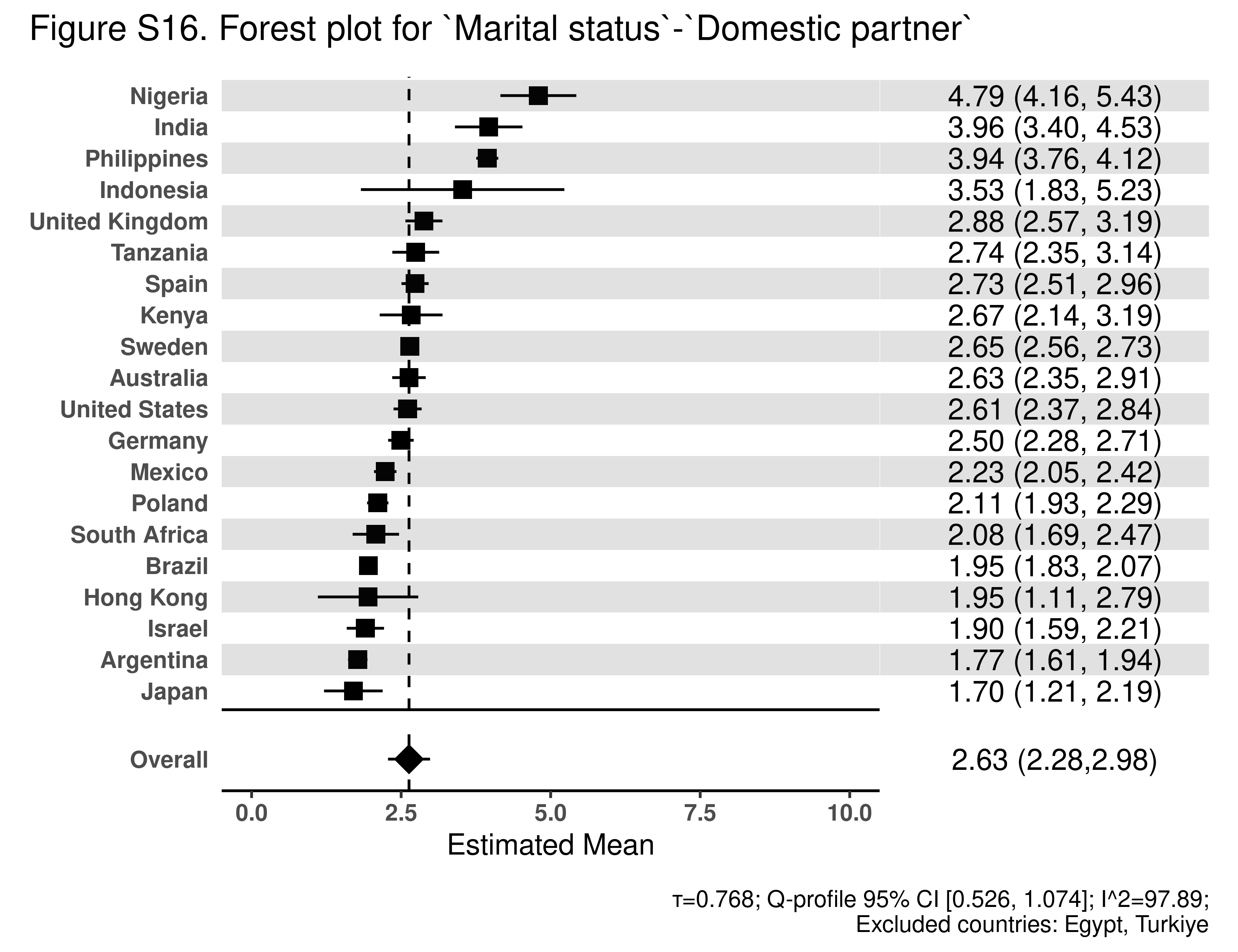

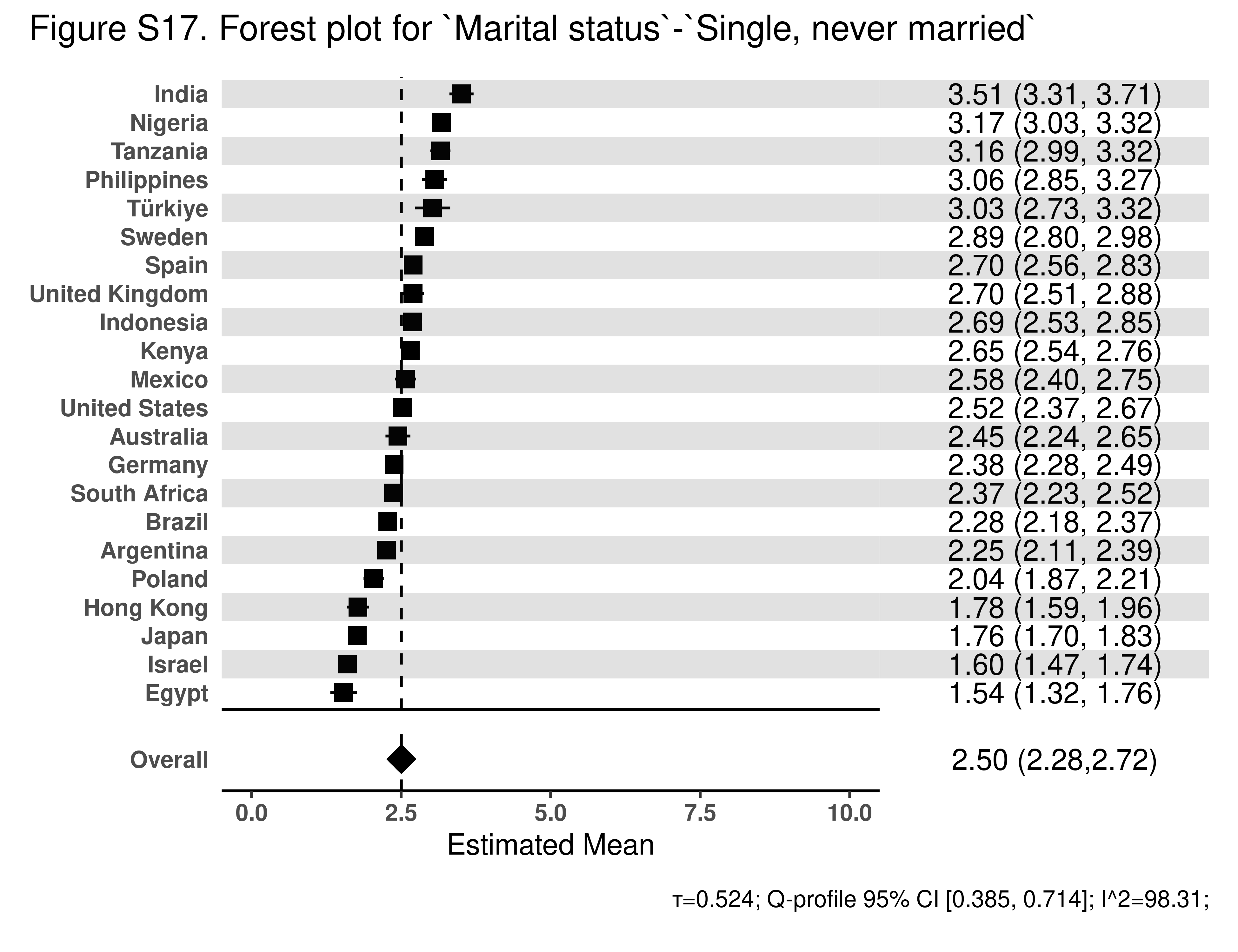

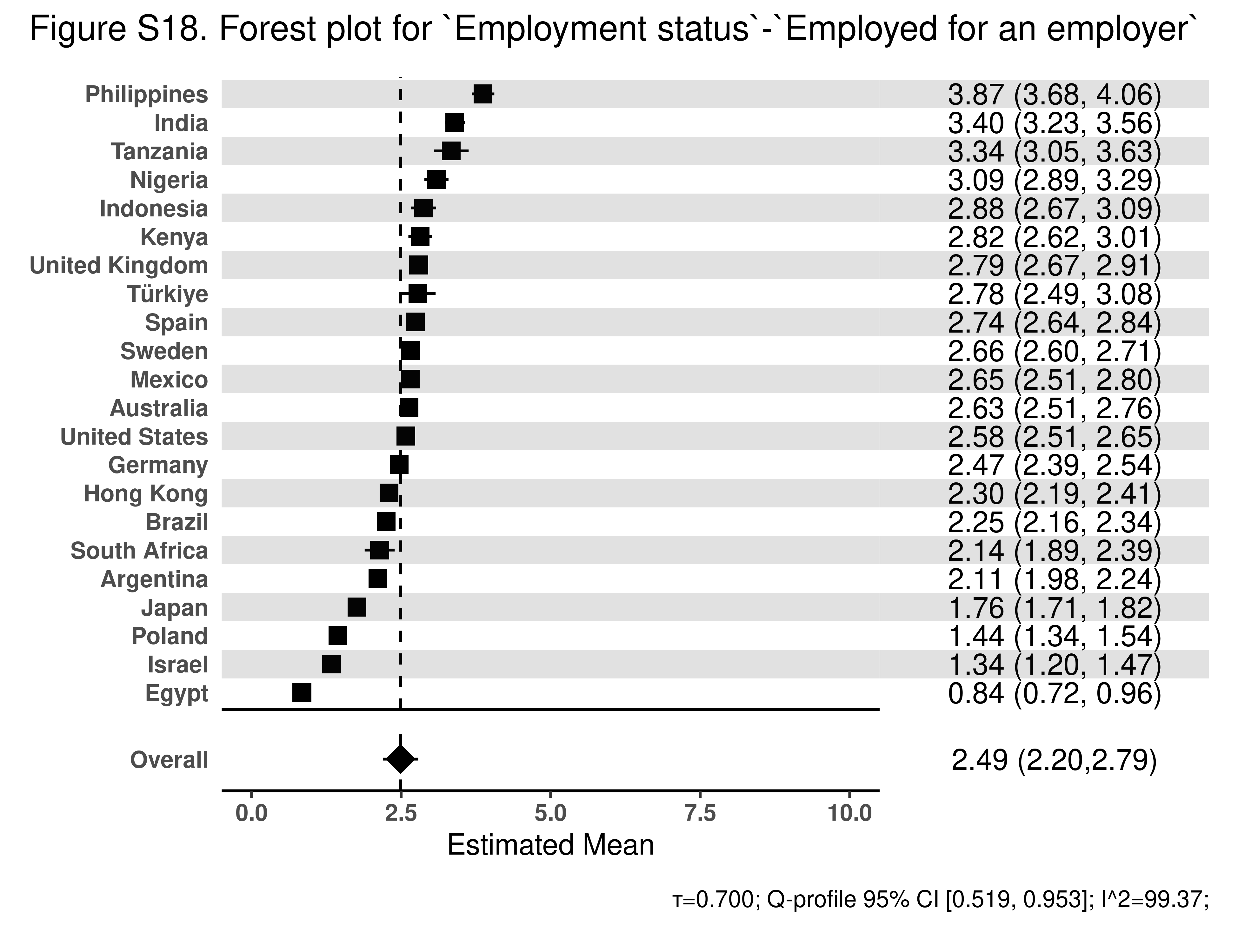

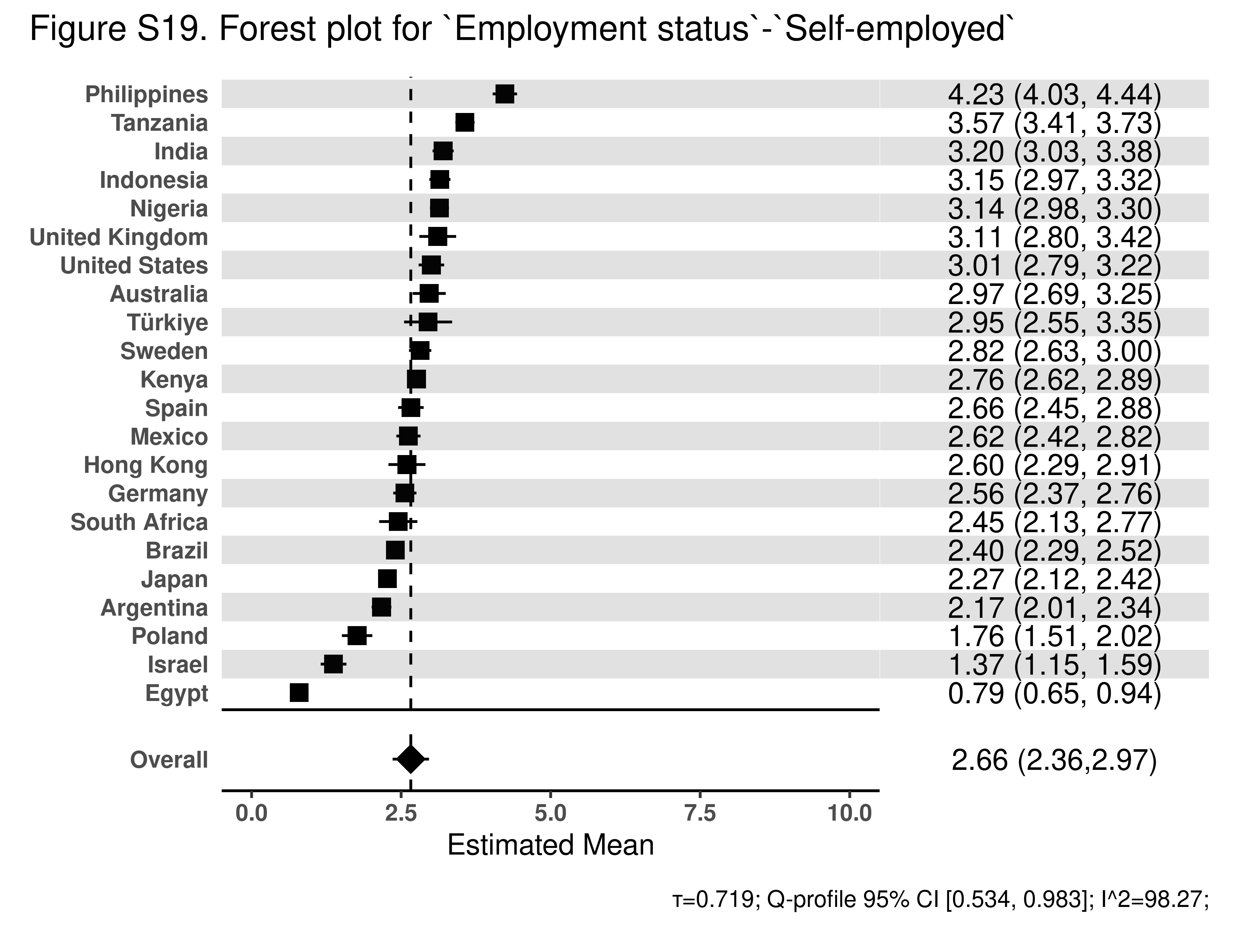

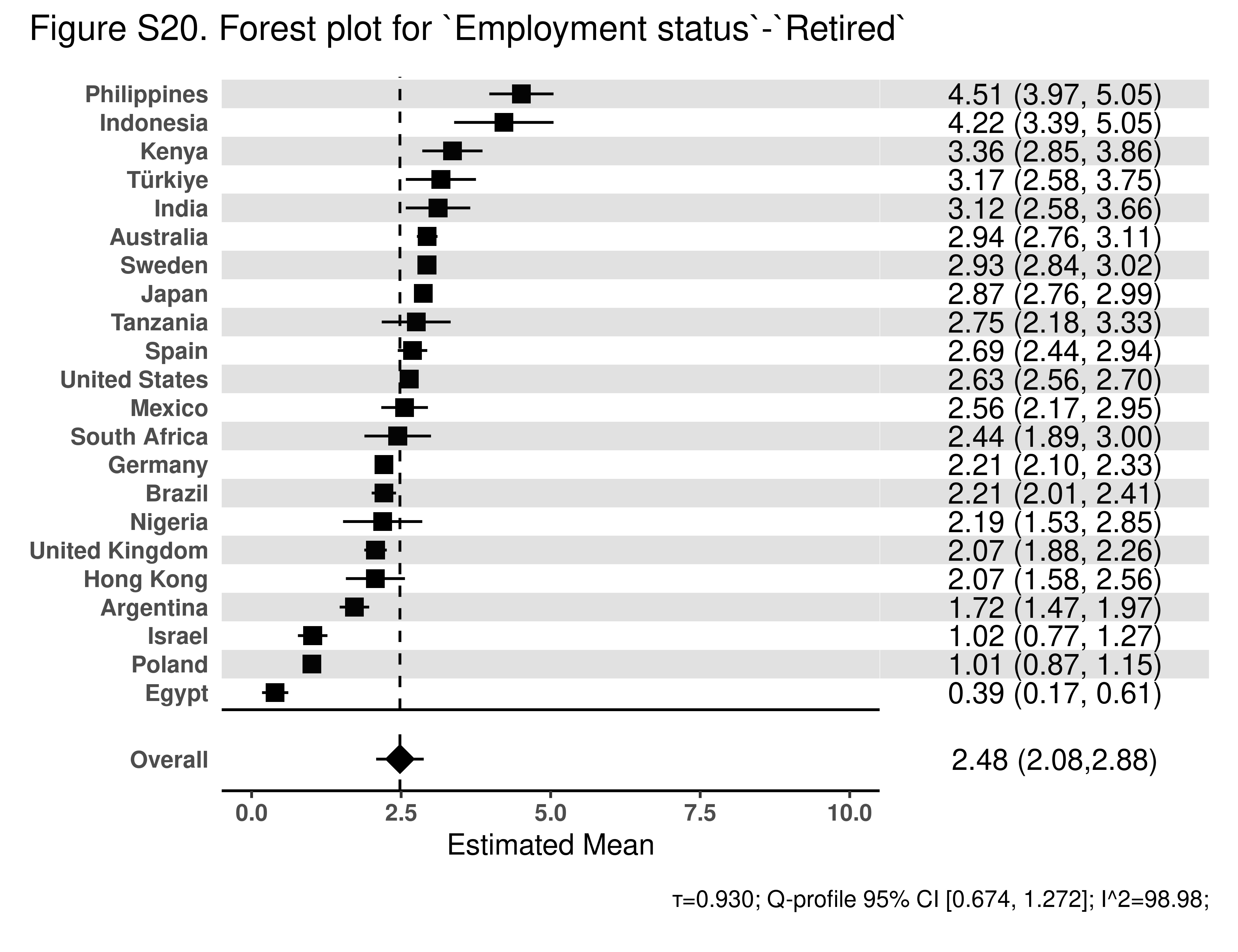

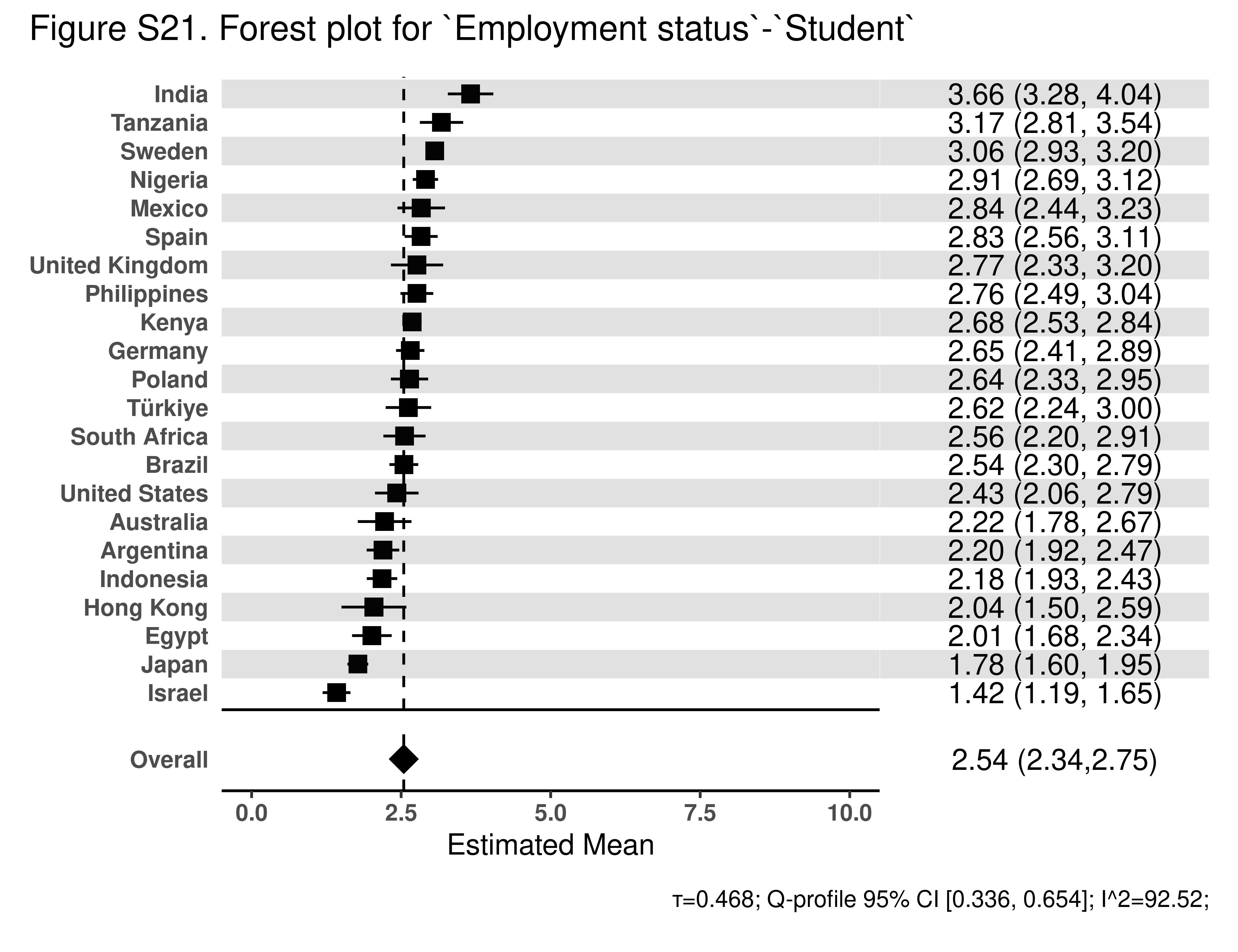

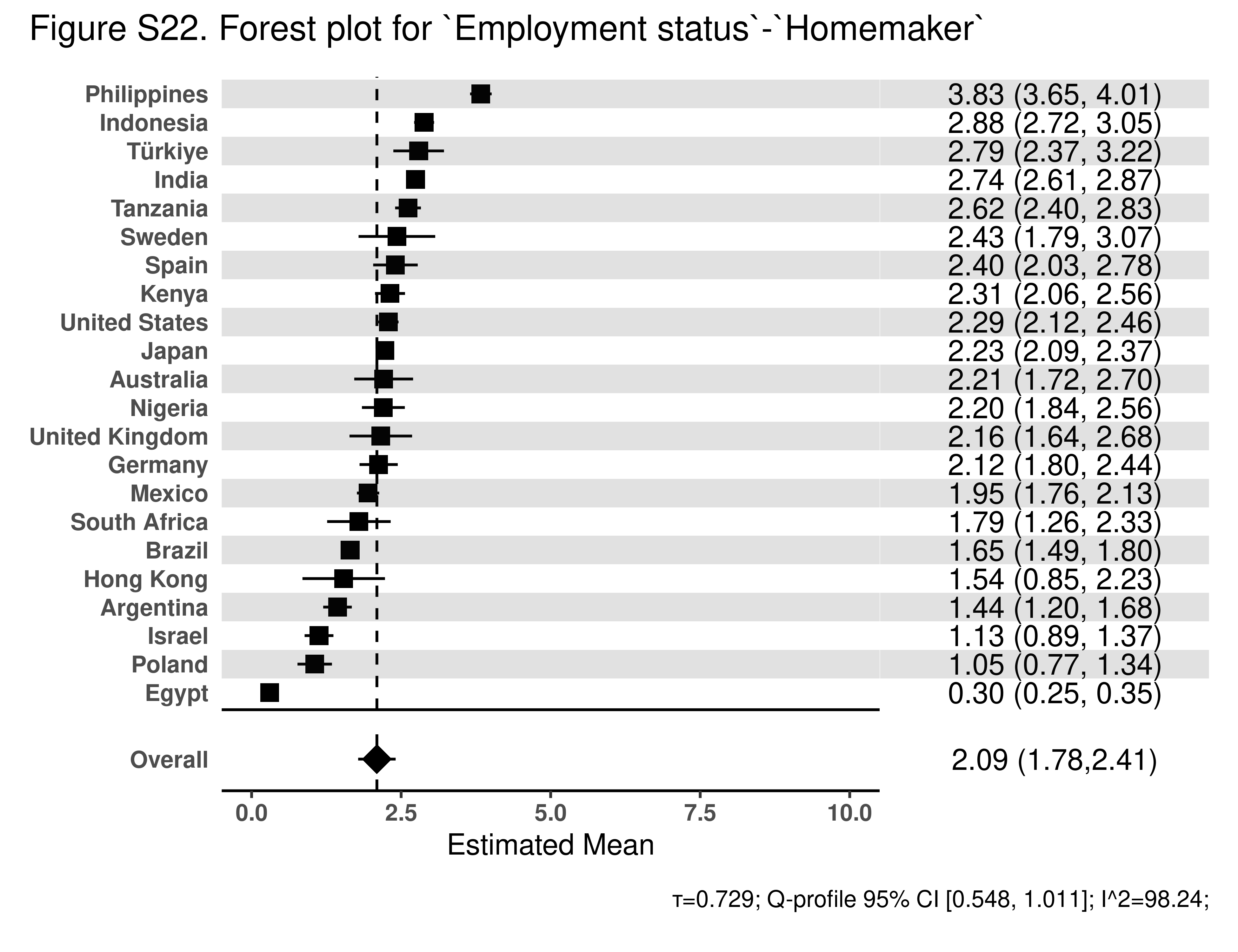

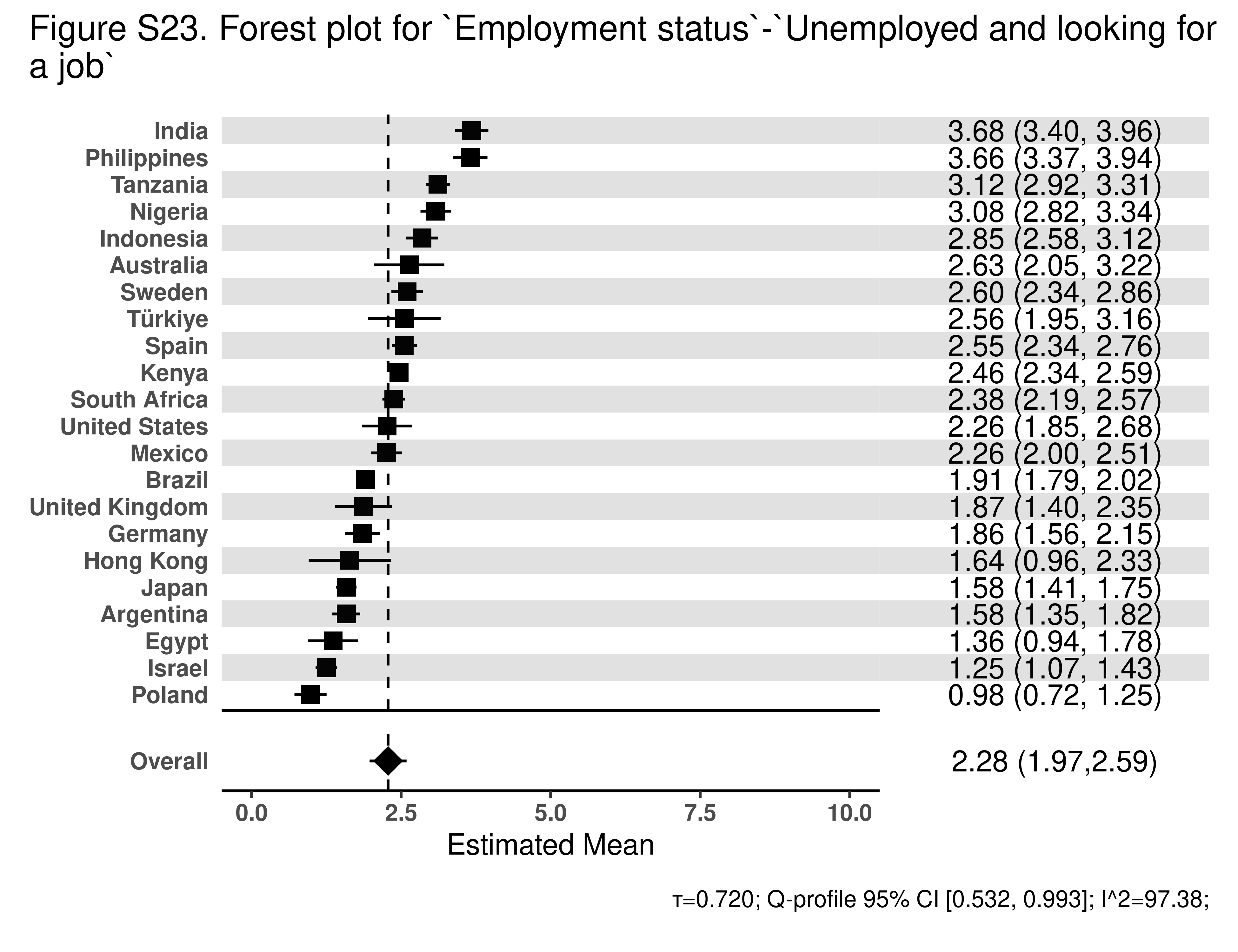

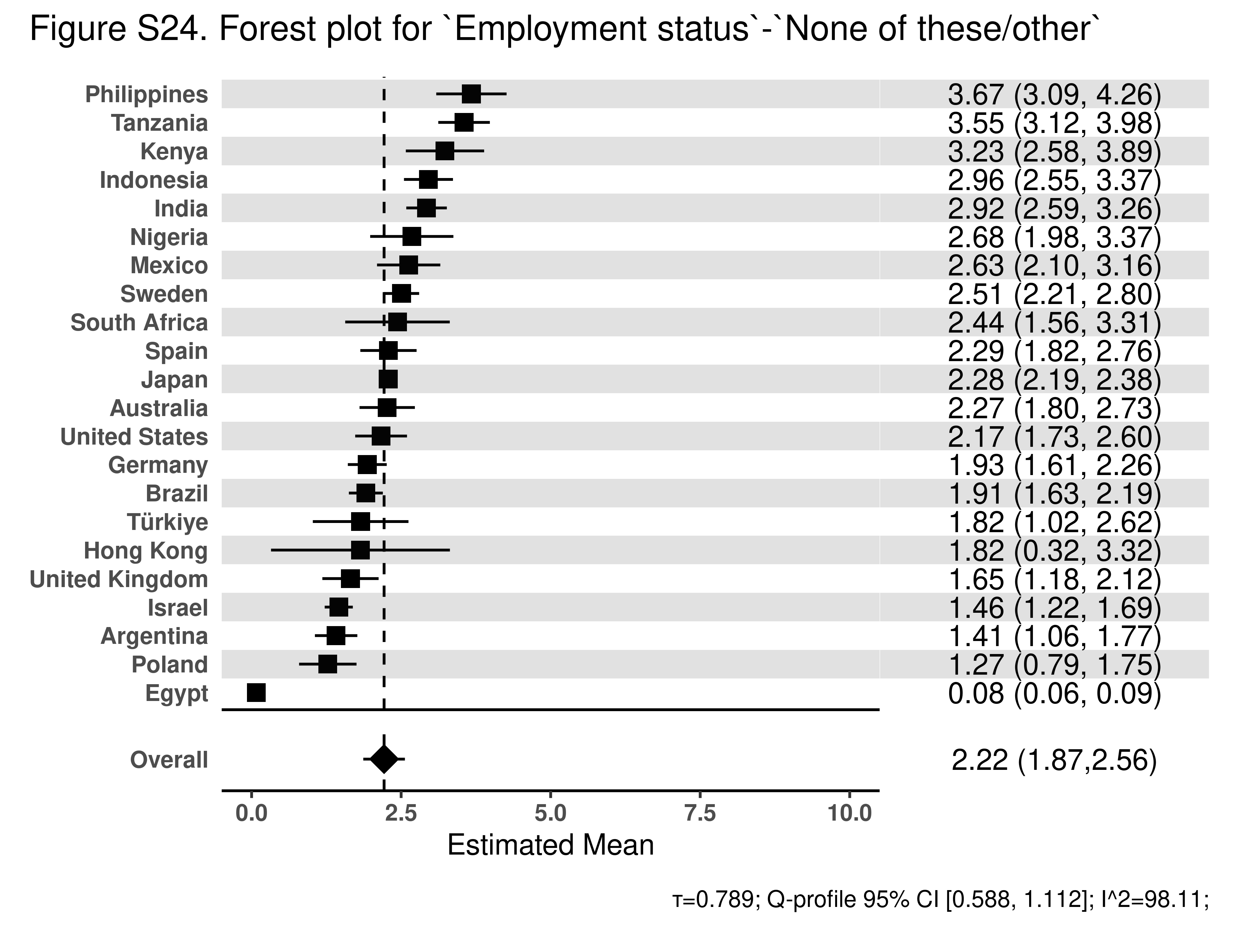

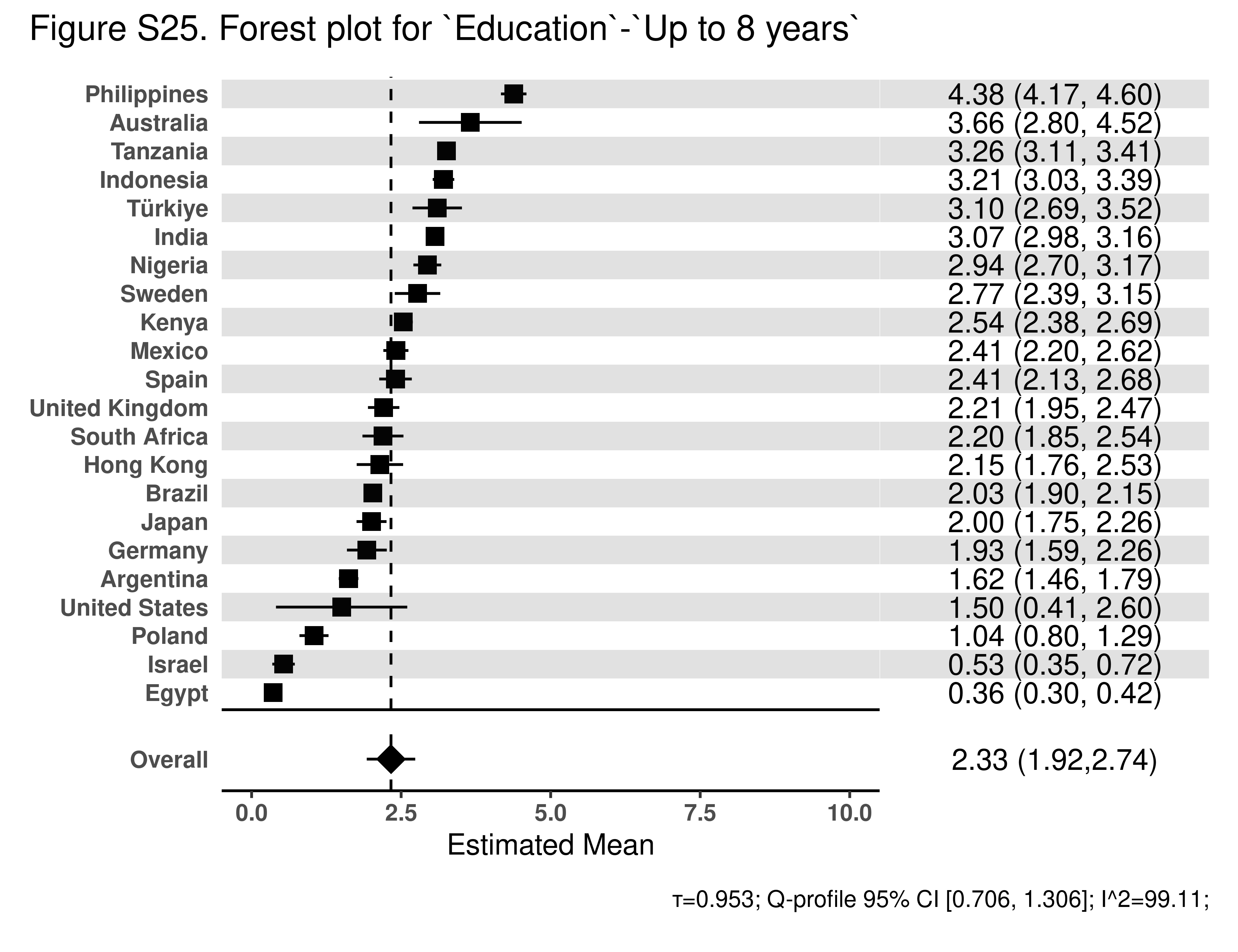

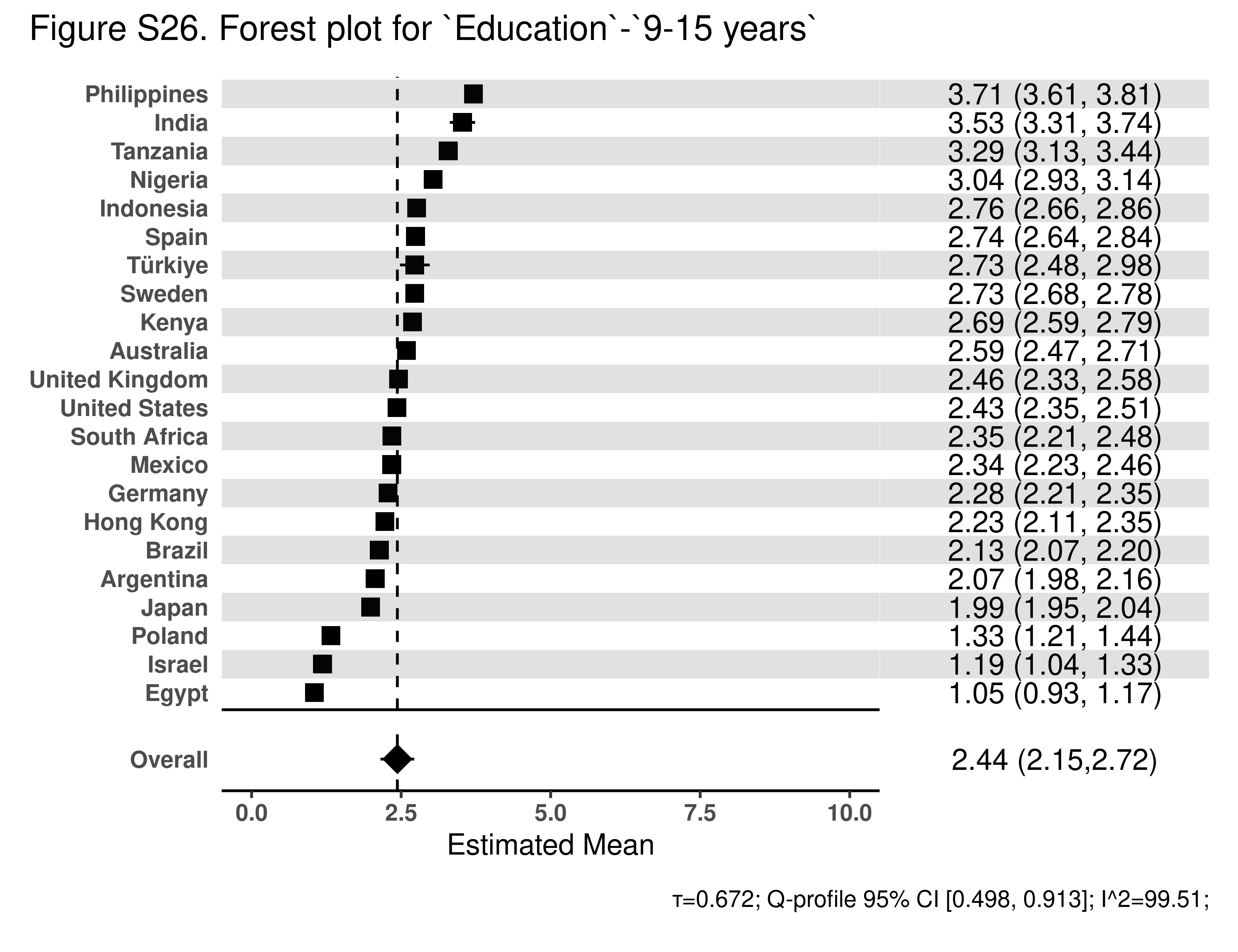

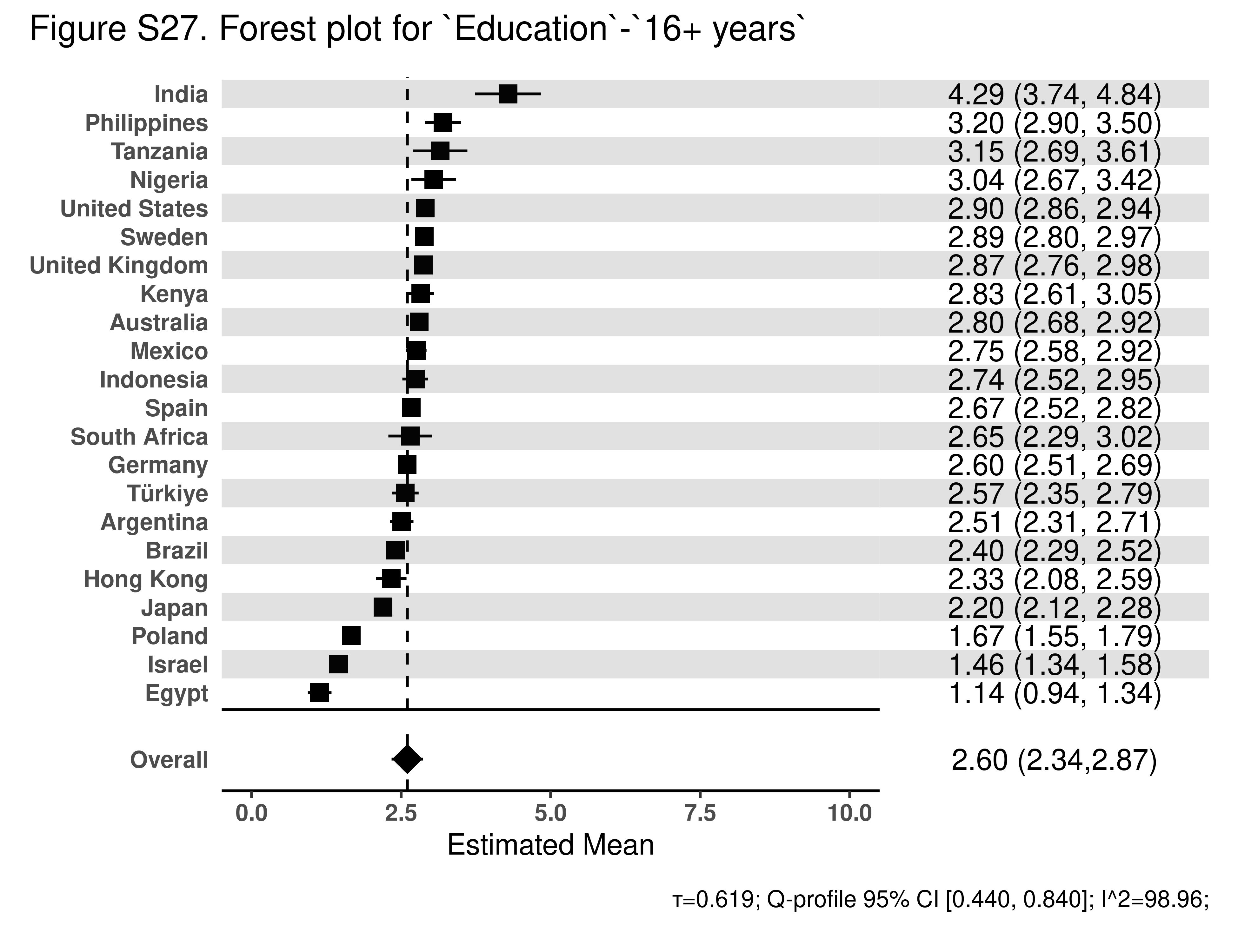

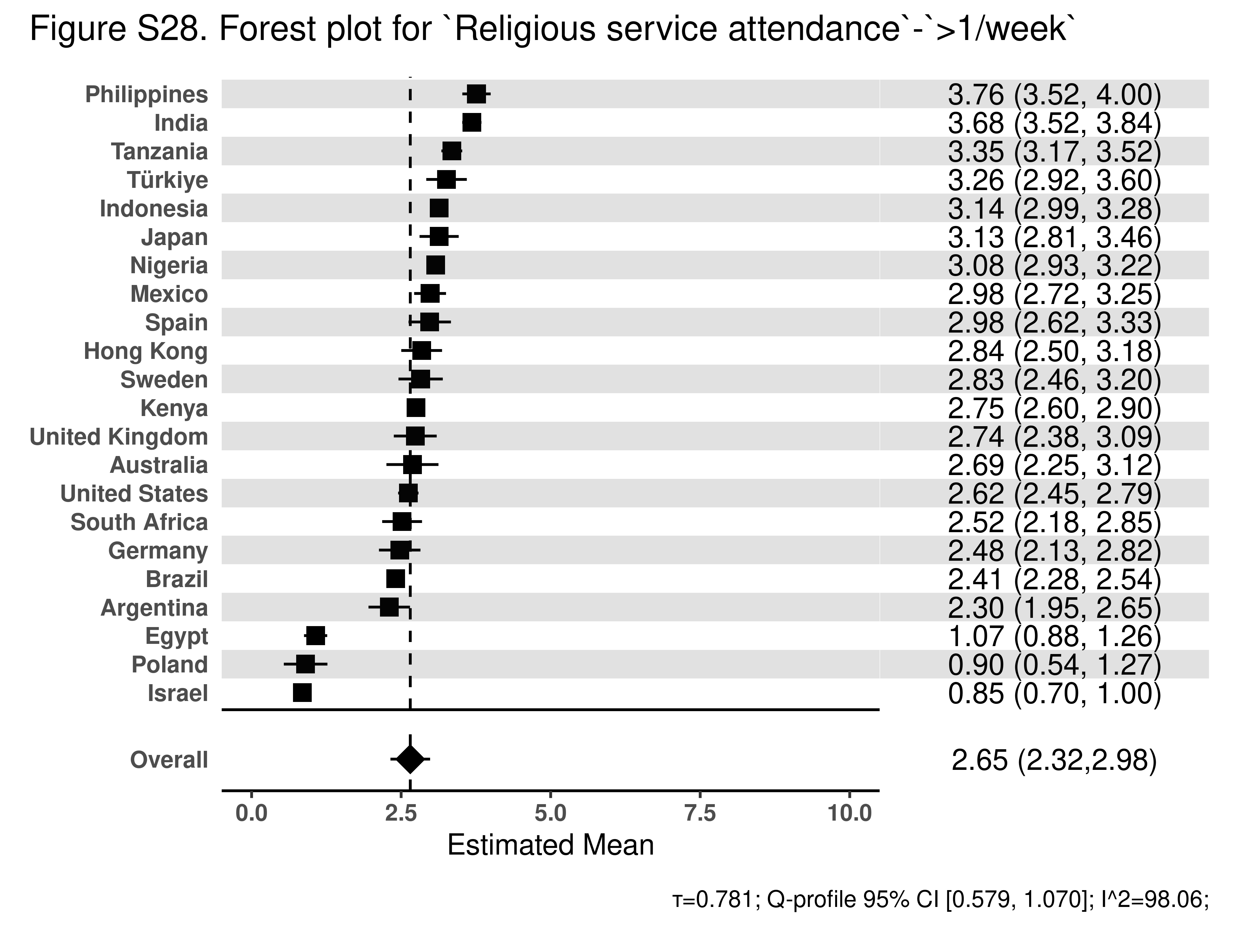

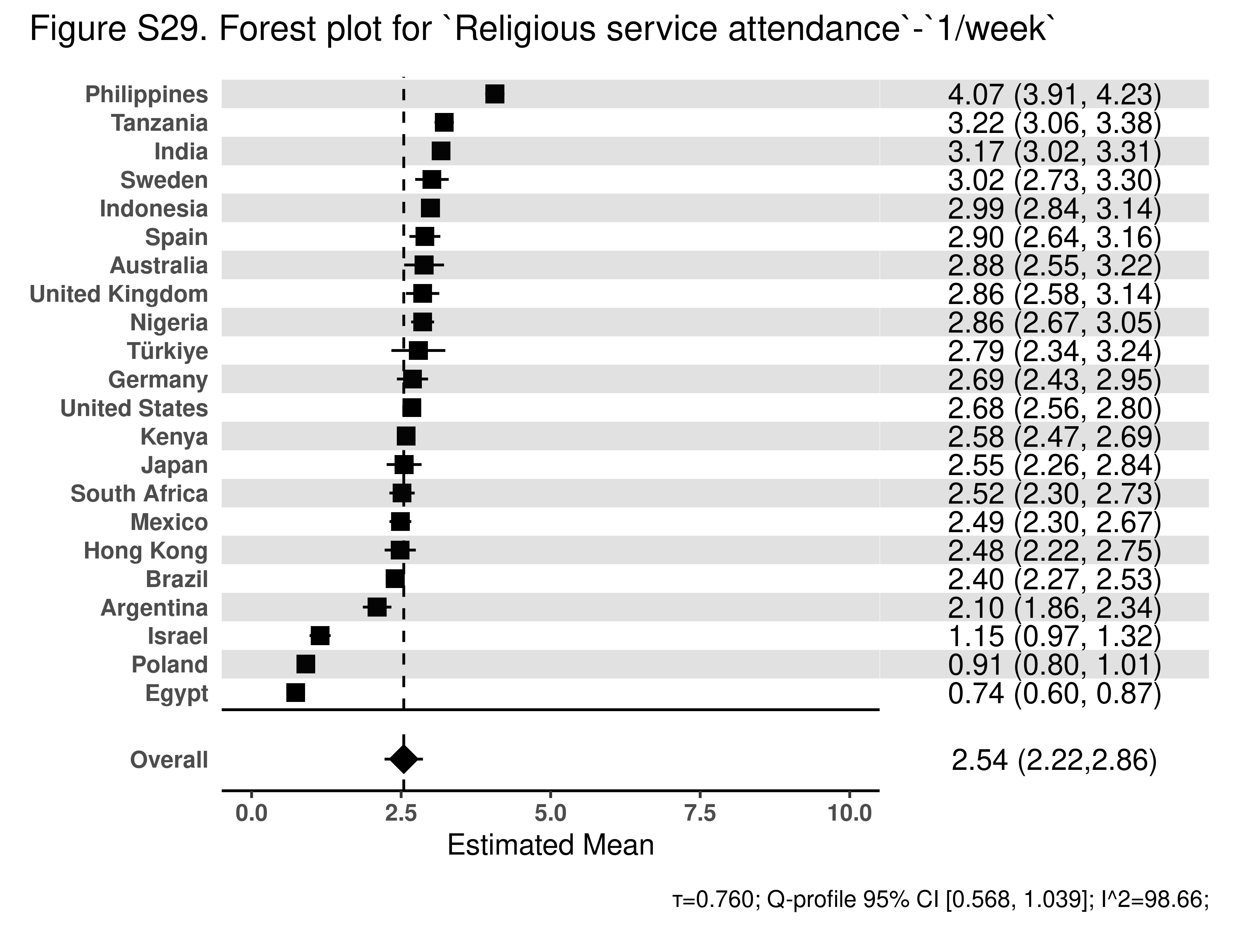

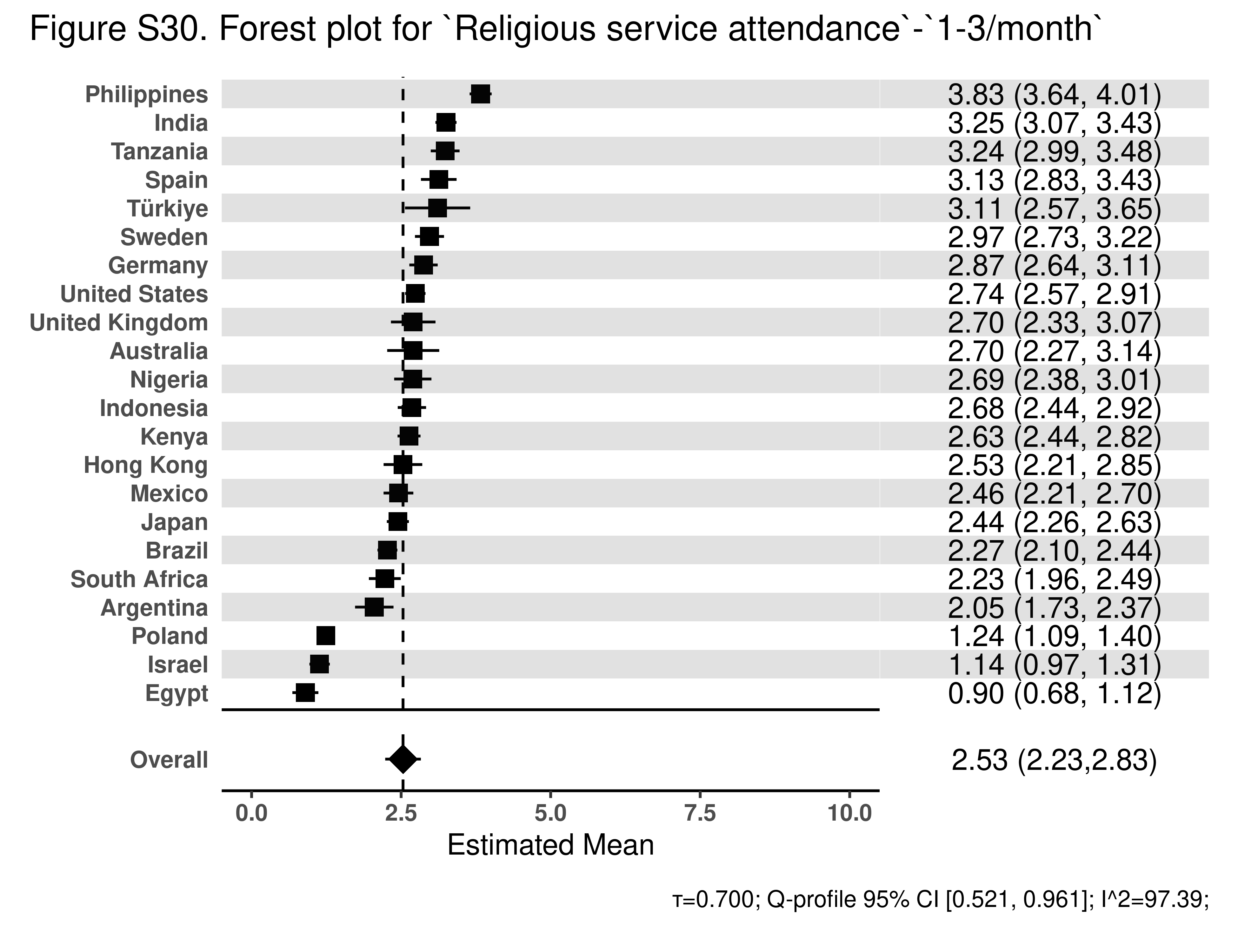

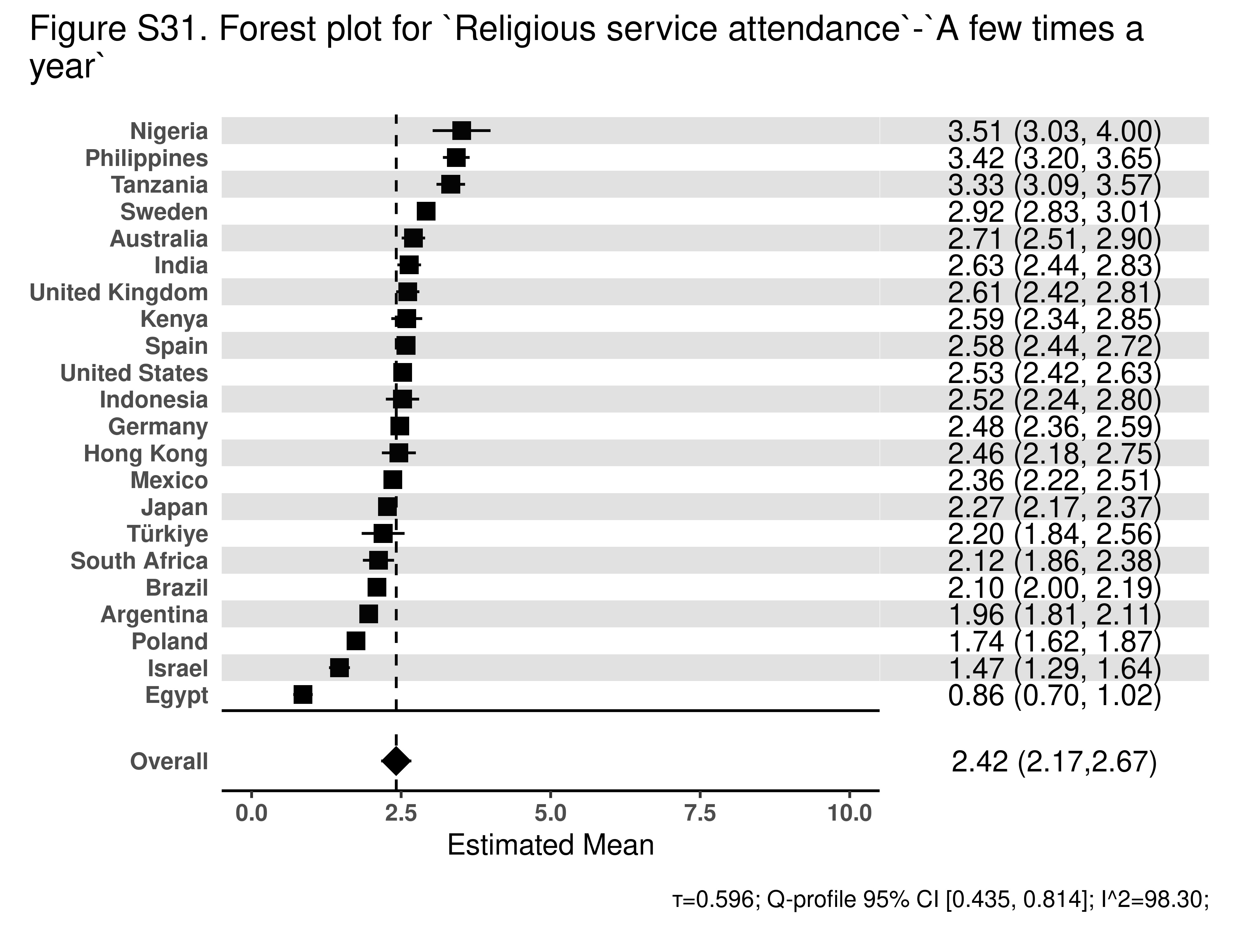

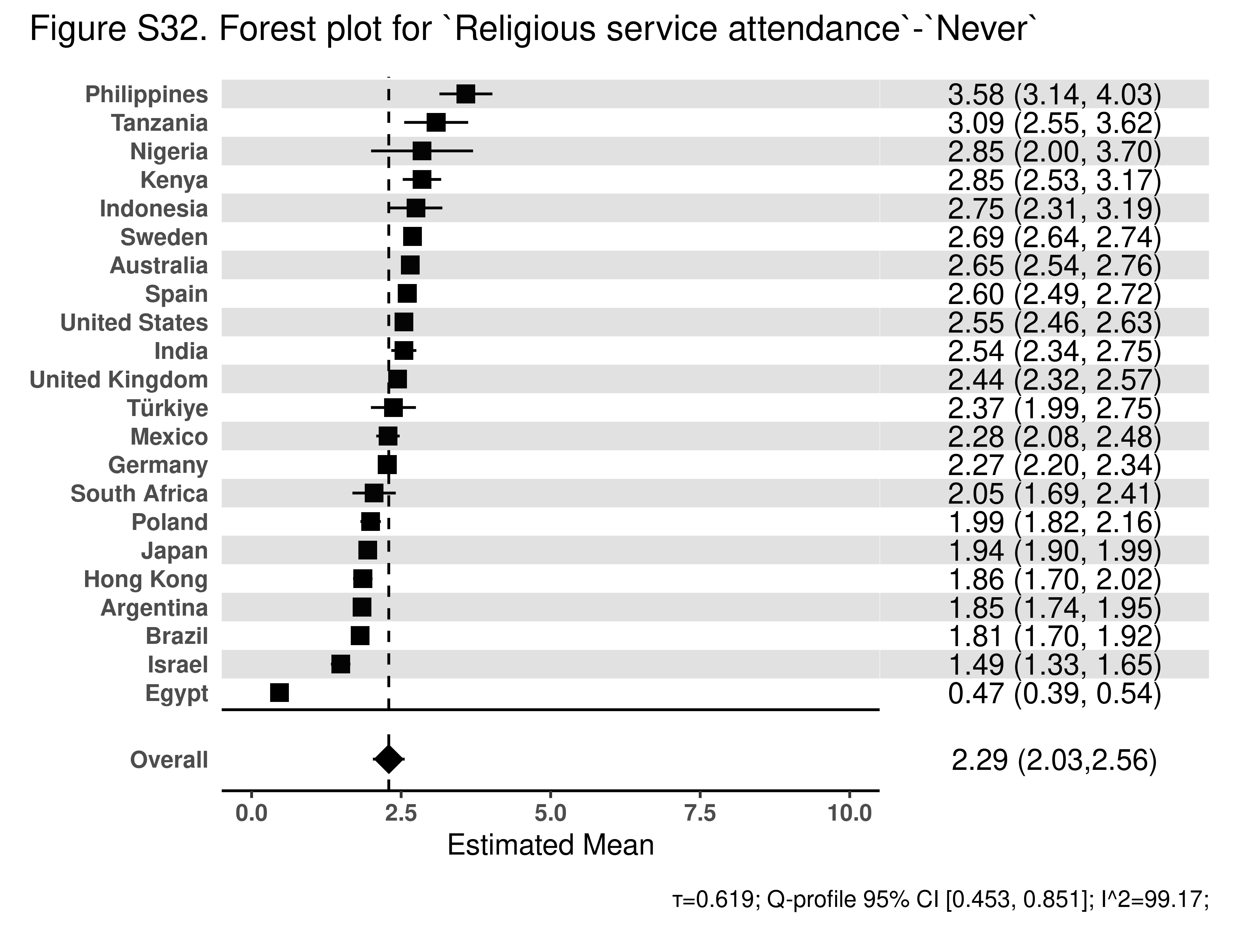

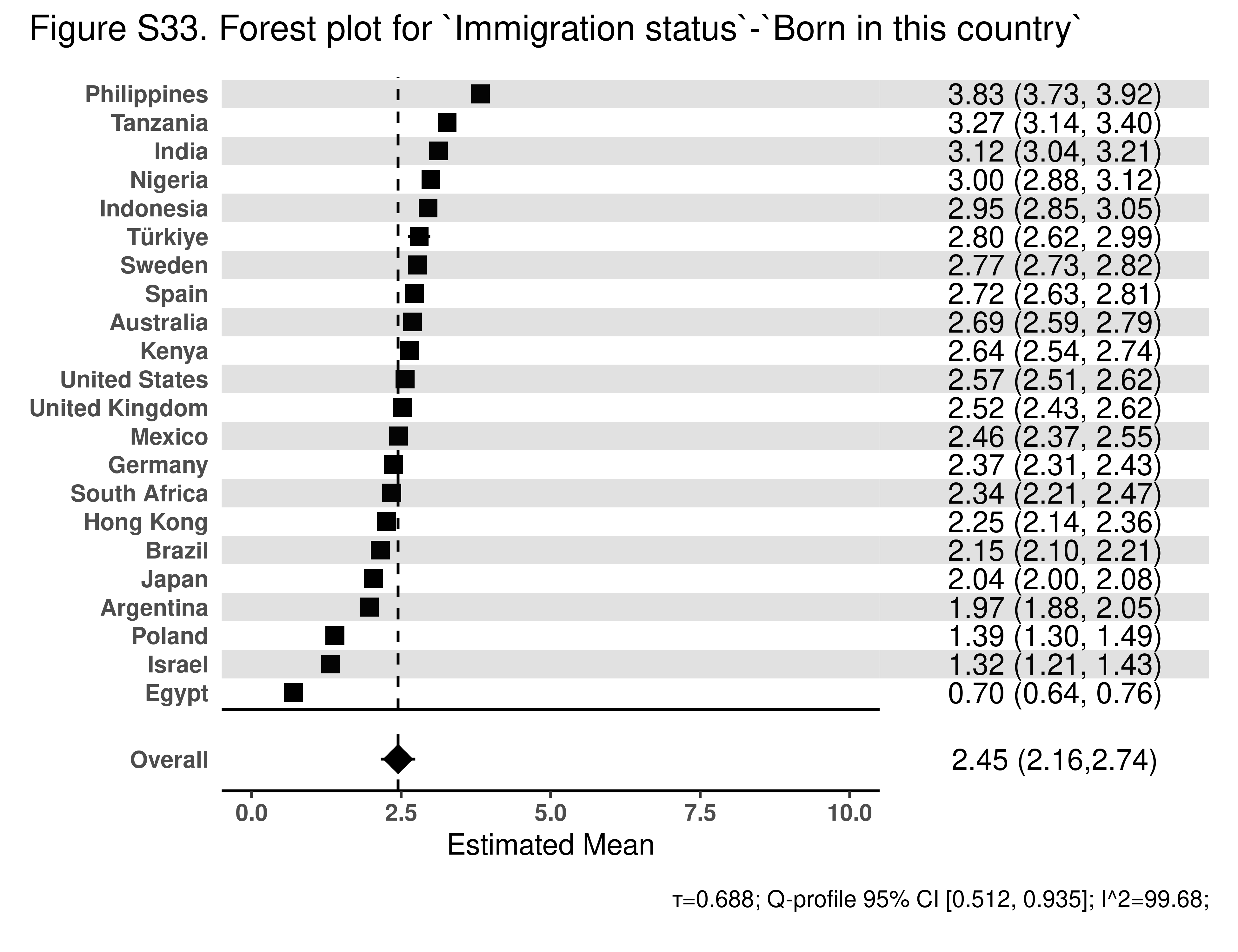

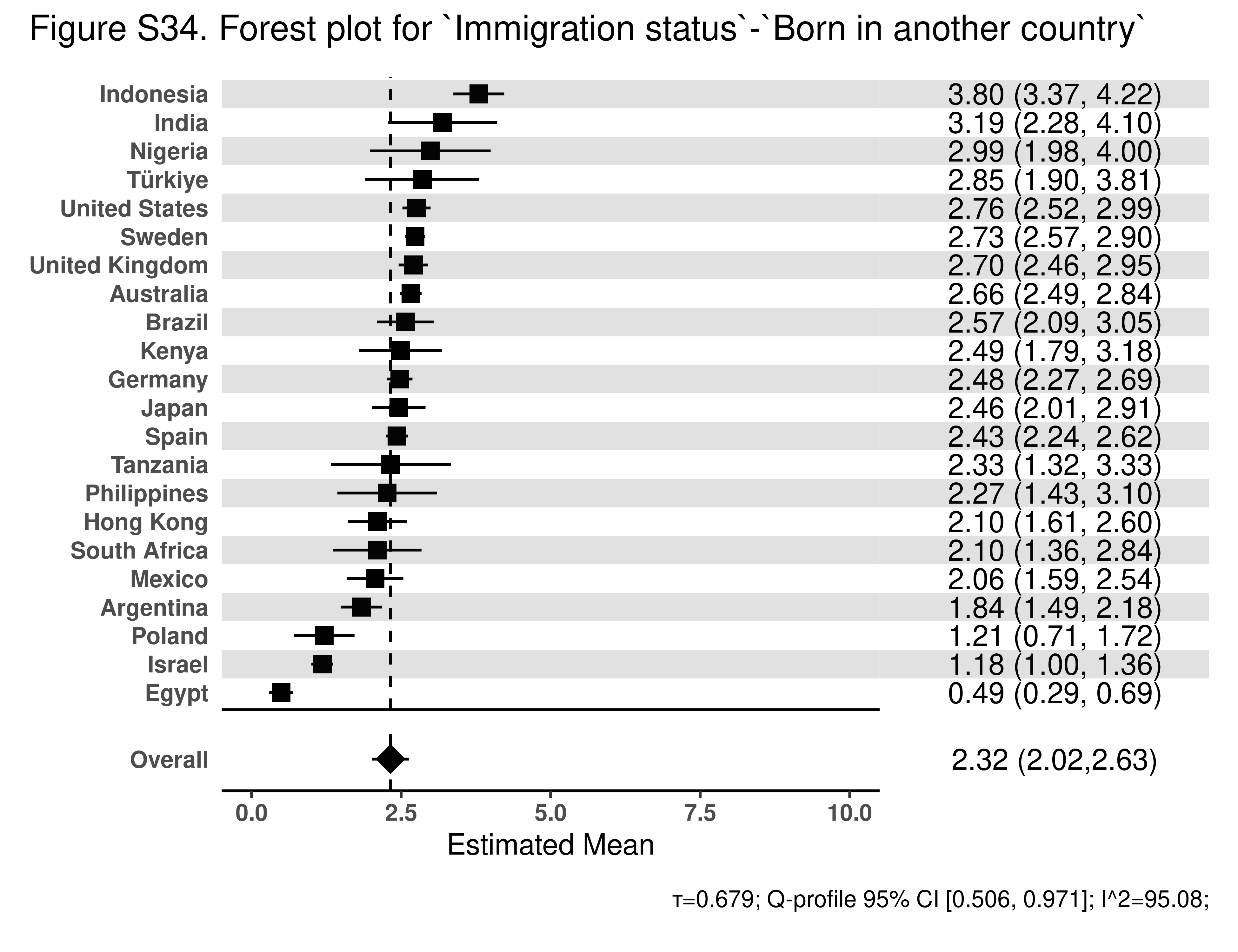


**Table 1a: Nationally Representative Descriptive Statistics of the Observed Sample (Argentina)**

| Variable | Proportion | Frequency |
| --- | --- | --- |
| Age |  |  |
| 18-24 | 0.16 | 1108 |
| 25-29 | 0.11 | 719 |
| 30-39 | 0.21 | 1432 |
| 40-49 | 0.19 | 1254 |
| 50-59 | 0.15 | 1014 |
| 60-69 | 0.11 | 730 |
| 70-79 | 0.05 | 356 |
| 80 or Older | 0.02 | 112 |
| Missing | . | . |
| Gender |  |  |
| Male | 0.47 | 3143 |
| Female | 0.53 | 3542 |
| Other | 0.00 | 21 |
| Missing | 0.00 | 18 |
| Marital Status |  |  |
| Single/Never Been Married | 0.35 | 2381 |
| Married | 0.23 | 1565 |
| Separated | 0.07 | 455 |
| Divorced | 0.05 | 321 |
| Widowed | 0.06 | 401 |
| Domestic Partner | 0.23 | 1514 |
| Missing | 0.01 | 88 |
| Employment |  |  |
| Employed for an Employer | 0.36 | 2440 |
| Self-Employed | 0.26 | 1748 |
| Retired | 0.11 | 773 |
| Student | 0.05 | 354 |
| Homemaker | 0.10 | 639 |
| Unemployed and Looking for a Job | 0.08 | 569 |
| None of These/Other | 0.03 | 179 |
| Missing | 0.00 | 22 |
| Education |  |  |
| Up to 8 Years | 0.34 | 2263 |
| 9-15 Years | 0.57 | 3823 |
| 16+ Years | 0.09 | 635 |
| Missing | 0.00 | 3 |
| Service Attendance |  |  |
| >1/Week | 0.08 | 532 |
| 1/Week | 0.12 | 773 |
| 1-3/Month | 0.07 | 461 |
| A Few Times a Year | 0.29 | 1949 |
| Never | 0.44 | 2982 |
| Missing | 0.00 | 27 |
| Immigration Status |  |  |
| Born in This Country | 0.94 | 6346 |
| Born in Another Country | 0.05 | 348 |
| Missing | 0.00 | 29 |
| Religion |  |  |
| Christianity | 0.74 | 4992 |
| Islam | 0.00 | 9 |
| Hinduism | 0.00 | 6 |
| Buddhism | 0.01 | 35 |
| Judaism | 0.01 | 40 |
| Sikhism | 0.00 | 0 |
| Baha'i | . | . |
| Jainism | . | . |
| Shinto | . | . |
| Taoism | 0.00 | 2 |
| Confucianism | 0.00 | 0 |
| Primal, Animist, or Folk Religion | 0.00 | 19 |
| Spiritism | . | . |
| African-Derived | . | . |
| Chinese | . | . |
| Some Other Religion | 0.02 | 156 |
| No Religion/Atheist/Agnostic | 0.20 | 1352 |
| Missing | 0.02 | 111 |
| Race/Ethnicity |  |  |
| Asian | 0.01 | 43 |
| Black | 0.01 | 95 |
| Indigenous | 0.02 | 129 |
| Mestizo(a) | 0.27 | 1801 |
| Mullato(a) | 0.01 | 75 |
| White | 0.51 | 3406 |
| Other | 0.02 | 104 |
| Missing | 0.16 | 1070 |

**Table 1b: Variations Across Demographic Characteristics (Argentina)**

| Variable | Mean | SE | LCI | UCI | Global p-value |
| --- | --- | --- | --- | --- | --- |
| Age |  |  |  |  |  |
| 18-24 | 2.40 | 0.10 | 2.20 | 2.60 | 0.00 |
| 25-29 | 2.13 | 0.13 | 1.88 | 2.39 | . |
| 30-39 | 1.97 | 0.08 | 2.20 | 2.60 | . |
| 40-49 | 1.82 | 0.09 | 1.88 | 2.39 | . |
| 50-59 | 1.89 | 0.11 | 1.81 | 2.13 | . |
| 60-69 | 1.53 | 0.11 | 1.65 | 1.99 | . |
| 70-79 | 1.96 | 0.22 | 1.69 | 2.10 | . |
| 80 or Older | 1.24 | 0.34 | 1.31 | 1.76 | . |
| Gender |  |  |  |  |  |
| Male | 2.23 | 0.06 | 2.11 | 2.35 | 0.00 |
| Female | 1.72 | 0.05 | 1.62 | 1.82 | . |
| Other | 1.81 | 0.54 | 0.69 | 2.94 | . |
| Marital Status |  |  |  |  |  |
| Single/Never Been Married | 2.25 | 0.07 | 2.12 | 2.39 | 0.00 |
| Married | 1.82 | 0.08 | 1.66 | 1.99 | . |
| Separated | 1.71 | 0.14 | 1.42 | 1.99 | . |
| Divorced | 1.85 | 0.15 | 1.56 | 2.14 | . |
| Widowed | 1.78 | 0.19 | 1.40 | 2.17 | . |
| Domestic Partner | 1.78 | 0.08 | 1.61 | 1.94 | . |
| Employment |  |  |  |  |  |
| Employed for an Employer | 2.11 | 0.07 | 1.98 | 2.24 | 0.00 |
| Self-Employed | 2.17 | 0.08 | 2.01 | 2.34 | . |
| Retired | 1.72 | 0.13 | 1.47 | 1.97 | . |
| Student | 2.20 | 0.14 | 1.92 | 2.47 | . |
| Homemaker | 1.44 | 0.12 | 1.20 | 1.68 | . |
| Unemployed and Looking for a Job | 1.58 | 0.12 | 1.35 | 1.82 | . |
| None of These/Other | 1.41 | 0.18 | 1.06 | 1.77 | . |
| Education |  |  |  |  |  |
| Up to 8 Years | 1.62 | 0.08 | 1.46 | 1.79 | 0.00 |
| 9-15 Years | 2.07 | 0.05 | 1.98 | 2.16 | . |
| 16+ Years | 2.51 | 0.10 | 2.31 | 2.71 | . |
| Service Attendance |  |  |  |  |  |
| >1/Week | 2.30 | 0.18 | 1.95 | 2.65 | 0.051 |
| 1/Week | 2.10 | 0.12 | 1.86 | 2.34 | . |
| 1-3/Month | 2.05 | 0.16 | 1.73 | 2.37 | . |
| A Few Times a Year | 1.96 | 0.08 | 1.81 | 2.11 | . |
| Never | 1.85 | 0.06 | 1.74 | 1.95 | . |
| Immigration Status |  |  |  |  |  |
| Born in This Country | 1.97 | 0.04 | 1.89 | 2.05 | 0.464 |
| Born in Another Country | 1.84 | 0.18 | 1.49 | 2.19 | . |
| Religion |  |  |  |  |  |
| Christianity | 1.91 | 0.05 | 1.81 | 2.00 | 0.00 |
| Islam | 2.31 | 0.92 | 0.17 | 4.45 | . |
| Hinduism | 2.17 | 0.57 | 0.42 | 3.91 | . |
| Buddhism | 2.95 | 0.35 | 2.25 | 3.65 | . |
| Judaism | 1.64 | 0.31 | 1.01 | 2.28 | . |
| Sikhism | 0.00 | . | . | . | . |
| Taoism | 1.98 | 1.03 | -1.75 | 5.72 | . |
| Confucianism | 1.02 | . | . | . | . |
| Primal, Animist, or Folk Religion | 3.07 | 0.92 | 1.06 | 5.09 | . |
| Some Other Religion | 1.82 | 0.26 | 1.30 | 2.34 | . |
| No Religion/Atheist/Agnostic | 2.13 | 0.08 | 1.97 | 2.29 | . |
| Race/Ethnicity |  |  |  |  |  |
| Asian | 2.06 | 0.44 | 1.16 | 2.95 | 0.785 |
| Black | 2.23 | 0.39 | 1.44 | 3.02 | . |
| Indigenous | 1.98 | 0.25 | 1.49 | 2.48 | . |
| Mestizo(a) | 1.87 | 0.08 | 1.71 | 2.03 | . |
| Mullato(a) | 2.13 | 0.39 | 1.35 | 2.91 | . |
| White | 1.99 | 0.05 | 1.89 | 2.10 | . |
| Other | 1.99 | 0.28 | 1.44 | 2.53 | . |

**Table 2a: Nationally Representative Descriptive Statistics of the Observed Sample (Australia)**

| Variable | Proportion | Frequency |
| --- | --- | --- |
| Age |  |  |
| 18-24 | 0.09 | 345 |
| 25-29 | 0.07 | 282 |
| 30-39 | 0.17 | 641 |
| 40-49 | 0.16 | 618 |
| 50-59 | 0.18 | 691 |
| 60-69 | 0.15 | 589 |
| 70-79 | 0.13 | 498 |
| 80 or Older | 0.05 | 178 |
| Missing | 0.00 | 2 |
| Gender |  |  |
| Male | 0.48 | 1861 |
| Female | 0.50 | 1941 |
| Other | 0.01 | 36 |
| Missing | 0.00 | 6 |
| Marital Status |  |  |
| Single/Never Been Married | 0.22 | 855 |
| Married | 0.47 | 1797 |
| Separated | 0.04 | 158 |
| Divorced | 0.09 | 332 |
| Widowed | 0.06 | 215 |
| Domestic Partner | 0.12 | 450 |
| Missing | 0.01 | 38 |
| Employment |  |  |
| Employed for an Employer | 0.49 | 1881 |
| Self-Employed | 0.10 | 380 |
| Retired | 0.24 | 912 |
| Student | 0.05 | 190 |
| Homemaker | 0.04 | 137 |
| Unemployed and Looking for a Job | 0.03 | 134 |
| None of These/Other | 0.05 | 206 |
| Missing | 0.00 | 4 |
| Education |  |  |
| Up to 8 Years | 0.02 | 70 |
| 9-15 Years | 0.63 | 2434 |
| 16+ Years | 0.35 | 1330 |
| Missing | 0.00 | 10 |
| Service Attendance |  |  |
| >1/Week | 0.04 | 162 |
| 1/Week | 0.08 | 299 |
| 1-3/Month | 0.04 | 135 |
| A Few Times a Year | 0.17 | 656 |
| Never | 0.67 | 2584 |
| Missing | 0.00 | 7 |
| Immigration Status |  |  |
| Born in This Country | 0.77 | 2953 |
| Born in Another Country | 0.23 | 885 |
| Missing | 0.00 | 6 |
| Religion |  |  |
| Christianity | 0.41 | 1592 |
| Islam | 0.01 | 45 |
| Hinduism | 0.01 | 31 |
| Buddhism | 0.01 | 36 |
| Judaism | 0.01 | 26 |
| Sikhism | 0.00 | 8 |
| Baha'i | 0.00 | 7 |
| Jainism | . | . |
| Shinto | . | . |
| Taoism | 0.00 | 5 |
| Confucianism | . | . |
| Primal, Animist, or Folk Religion | 0.01 | 23 |
| Spiritism | . | . |
| African-Derived | . | . |
| Chinese | . | . |
| Some Other Religion | 0.01 | 39 |
| No Religion/Atheist/Agnostic | 0.53 | 2020 |
| Missing | 0.00 | 15 |
| Race/Ethnicity |  |  |
| Aboriginal | 0.01 | 53 |
| Australian | 0.51 | 1946 |
| Australian /British/European | 0.27 | 1047 |
| Chinese | 0.02 | 75 |
| Indian | 0.02 | 58 |
| Japanese | 0.00 | 1 |
| Malay | 0.00 | 11 |
| Sinhalese | 0.00 | 1 |
| Spanish | 0.00 | 2 |
| Sri Lankan Moor | 0.00 | 1 |
| Sri Lankan Tamil | 0.00 | 7 |
| Vietnamese | 0.00 | 7 |
| Taiwanese/Holo | . | . |
| Russian | 0.00 | 7 |
| Samoan | 0.00 | 4 |
| New Zealander | 0.02 | 91 |
| Other European | 0.09 | 357 |
| Other | 0.04 | 163 |
| Missing | 0.00 | 14 |

**Table 2b: Variations Across Demographic Characteristics (Australia)**

| Variable | Mean | SE | LCI | UCI | Global p-value |
| --- | --- | --- | --- | --- | --- |
| Age |  |  |  |  |  |
| 18-24 | 2.68 | 0.18 | 2.33 | 3.03 | 0.00 |
| 25-29 | 2.65 | 0.18 | 2.29 | 3.01 | . |
| 30-39 | 2.31 | 0.12 | 2.08 | 2.54 | . |
| 40-49 | 2.61 | 0.12 | 2.38 | 2.84 | . |
| 50-59 | 2.66 | 0.10 | 2.46 | 2.86 | . |
| 60-69 | 3.07 | 0.11 | 2.86 | 3.28 | . |
| 70-79 | 2.80 | 0.11 | 2.58 | 3.03 | . |
| 80 or Older | 2.80 | 0.21 | 2.39 | 3.22 | . |
| Gender |  |  |  |  |  |
| Male | 2.86 | 0.07 | 2.73 | 2.99 | 0.00 |
| Female | 2.53 | 0.06 | 2.40 | 2.65 | . |
| Other | 2.14 | 0.44 | 1.23 | 3.04 | . |
| Marital Status |  |  |  |  |  |
| Single/Never Been Married | 2.45 | 0.11 | 2.24 | 2.65 | 0.040 |
| Married | 2.83 | 0.06 | 2.71 | 2.95 | . |
| Separated | 2.50 | 0.25 | 2.02 | 2.99 | . |
| Divorced | 2.61 | 0.16 | 2.30 | 2.91 | . |
| Widowed | 2.76 | 0.19 | 2.38 | 3.14 | . |
| Domestic Partner | 2.63 | 0.14 | 2.35 | 2.91 | . |
| Employment |  |  |  |  |  |
| Employed for an Employer | 2.63 | 0.06 | 2.51 | 2.76 | 0.00 |
| Self-Employed | 2.97 | 0.14 | 2.69 | 3.25 | . |
| Retired | 2.94 | 0.09 | 2.76 | 3.11 | . |
| Student | 2.22 | 0.23 | 1.77 | 2.68 | . |
| Homemaker | 2.21 | 0.25 | 1.71 | 2.71 | . |
| Unemployed and Looking for a Job | 2.64 | 0.30 | 2.04 | 3.23 | . |
| None of These/Other | 2.27 | 0.24 | 1.80 | 2.73 | . |
| Education |  |  |  |  |  |
| Up to 8 Years | 3.66 | 0.44 | 2.76 | 4.55 | 0.005 |
| 9-15 Years | 2.59 | 0.06 | 2.47 | 2.71 | . |
| 16+ Years | 2.80 | 0.06 | 2.68 | 2.92 | . |
| Service Attendance |  |  |  |  |  |
| >1/Week | 2.69 | 0.22 | 2.25 | 3.13 | 0.786 |
| 1/Week | 2.88 | 0.17 | 2.55 | 3.22 | . |
| 1-3/Month | 2.70 | 0.22 | 2.26 | 3.14 | . |
| A Few Times a Year | 2.70 | 0.10 | 2.51 | 2.90 | . |
| Never | 2.65 | 0.06 | 2.54 | 2.77 | . |
| Immigration Status |  |  |  |  |  |
| Born in This Country | 2.69 | 0.05 | 2.58 | 2.79 | 0.815 |
| Born in Another Country | 2.66 | 0.09 | 2.49 | 2.84 | . |
| Religion |  |  |  |  |  |
| Christianity | 2.72 | 0.07 | 2.58 | 2.86 | 0.00 |
| Islam | 2.29 | 0.43 | 1.42 | 3.16 | . |
| Hinduism | 2.05 | 0.40 | 1.24 | 2.87 | . |
| Buddhism | 2.93 | 0.50 | 1.92 | 3.94 | . |
| Judaism | 2.25 | 0.51 | 1.21 | 3.29 | . |
| Sikhism | 1.82 | . | . | . | . |
| Baha'i | 1.76 | 0.09 | 1.50 | 2.02 | . |
| Taoism | 5.25 | . | . | . | . |
| Primal, Animist, or Folk Religion | 2.00 | 0.43 | 1.07 | 2.93 | . |
| Some Other Religion | 2.26 | 0.53 | 1.17 | 3.35 | . |
| No Religion/Atheist/Agnostic | 2.69 | 0.06 | 2.57 | 2.82 | . |
| Race/Ethnicity |  |  |  |  |  |
| Aboriginal | 2.02 | 0.38 | 1.25 | 2.79 | 0.00 |
| Australian | 2.70 | 0.07 | 2.57 | 2.83 | . |
| Australian /British/European | 2.65 | 0.09 | 2.48 | 2.82 | . |
| Chinese | 2.69 | 0.29 | 2.11 | 3.27 | . |
| Indian | 1.75 | 0.23 | 1.28 | 2.22 | . |
| Japanese | 4.86 | . | . | . | . |
| Malay | 2.81 | 1.02 | 0.20 | 5.42 | . |
| Sinhalese | 3.57 | . | . | . | . |
| Spanish | 1.72 | . | . | . | . |
| Sri Lankan Moor | 0.30 | . | . | . | . |
| Sri Lankan Tamil | 3.02 | 1.76 | -2.12 | 8.16 | . |
| Vietnamese | 4.04 | 0.76 | . | . | . |
| Russian | 1.72 | 0.77 | 2.09 | 5.98 | . |
| Samoan | 0.03 | . | . | . | . |
| New Zealander | 3.43 | 0.35 | 2.73 | 4.13 | . |
| Other European | 2.85 | 0.14 | 2.58 | 3.13 | . |
| Other | 2.45 | 0.20 | 2.05 | 2.86 | . |

**Table 3a: Nationally Representative Descriptive Statistics of the Observed Sample (Brazil)**

| Variable | Proportion | Frequency |
| --- | --- | --- |
| Age |  |  |
| 18-24 | 0.15 | 1986 |
| 25-29 | 0.11 | 1468 |
| 30-39 | 0.22 | 2908 |
| 40-49 | 0.20 | 2638 |
| 50-59 | 0.16 | 2131 |
| 60-69 | 0.11 | 1435 |
| 70-79 | 0.04 | 510 |
| 80 or Older | 0.01 | 126 |
| Missing | . | . |
| Gender |  |  |
| Male | 0.48 | 6320 |
| Female | 0.52 | 6820 |
| Other | 0.00 | 35 |
| Missing | 0.00 | 30 |
| Marital Status |  |  |
| Single/Never Been Married | 0.33 | 4347 |
| Married | 0.35 | 4646 |
| Separated | 0.04 | 594 |
| Divorced | 0.07 | 865 |
| Widowed | 0.03 | 408 |
| Domestic Partner | 0.16 | 2081 |
| Missing | 0.02 | 263 |
| Employment |  |  |
| Employed for an Employer | 0.28 | 3756 |
| Self-Employed | 0.22 | 2918 |
| Retired | 0.12 | 1536 |
| Student | 0.05 | 624 |
| Homemaker | 0.10 | 1305 |
| Unemployed and Looking for a Job | 0.18 | 2419 |
| None of These/Other | 0.03 | 448 |
| Missing | 0.02 | 199 |
| Education |  |  |
| Up to 8 Years | 0.24 | 3139 |
| 9-15 Years | 0.58 | 7665 |
| 16+ Years | 0.18 | 2390 |
| Missing | 0.00 | 10 |
| Service Attendance |  |  |
| >1/Week | 0.18 | 2386 |
| 1/Week | 0.17 | 2272 |
| 1-3/Month | 0.11 | 1398 |
| A Few Times a Year | 0.30 | 3978 |
| Never | 0.24 | 3110 |
| Missing | 0.00 | 61 |
| Immigration Status |  |  |
| Born in This Country | 0.96 | 12688 |
| Born in Another Country | 0.01 | 153 |
| Missing | 0.03 | 363 |
| Religion |  |  |
| Christianity | 0.75 | 9911 |
| Islam | 0.00 | 6 |
| Hinduism | 0.00 | 1 |
| Buddhism | 0.00 | 37 |
| Judaism | 0.00 | 31 |
| Sikhism | . | . |
| Baha'i | 0.00 | 2 |
| Jainism | 0.00 | 2 |
| Shinto | 0.00 | 1 |
| Taoism | 0.00 | 2 |
| Confucianism | 0.00 | 6 |
| Primal, Animist, or Folk Religion | 0.00 | 15 |
| Spiritism | 0.05 | 696 |
| African-Derived | 0.04 | 525 |
| Chinese | . | . |
| Some Other Religion | 0.01 | 144 |
| No Religion/Atheist/Agnostic | 0.13 | 1712 |
| Missing | 0.01 | 113 |
| Race/Ethnicity |  |  |
| Branca | 0.39 | 5169 |
| Preta | 0.12 | 1615 |
| Parda | 0.39 | 5125 |
| Amarela | 0.02 | 238 |
| Indigena | 0.01 | 131 |
| Other | 0.00 | 61 |
| Missing | 0.07 | 865 |

**Table 3b: Variations Across Demographic Characteristics (Brazil)**

| Variable | Mean | SE | LCI | UCI | Global p-value |
| --- | --- | --- | --- | --- | --- |
| Age |  |  |  |  |  |
| 18-24 | 2.33 | 0.06 | 2.20 | 2.45 | 0.024 |
| 25-29 | 2.29 | 0.08 | 2.13 | 2.44 | . |
| 30-39 | 2.18 | 0.05 | 2.08 | 2.28 | . |
| 40-49 | 2.03 | 0.06 | 1.92 | 2.15 | . |
| 50-59 | 2.10 | 0.07 | 1.96 | 2.23 | . |
| 60-69 | 2.12 | 0.10 | 1.92 | 2.32 | . |
| 70-79 | 2.03 | 0.18 | 1.66 | 2.39 | . |
| 80 or Older | 2.06 | 0.40 | 1.26 | 2.85 | . |
| Gender |  |  |  |  |  |
| Male | 2.44 | 0.04 | 2.35 | 2.52 | 0.00 |
| Female | 1.90 | 0.04 | 1.83 | 1.97 | . |
| Other | 2.11 | 0.43 | 1.23 | 3.00 | . |
| Marital Status |  |  |  |  |  |
| Single/Never Been Married | 2.28 | 0.05 | 2.18 | 2.37 | 0.00 |
| Married | 2.20 | 0.05 | 2.11 | 2.29 | . |
| Separated | 2.34 | 0.14 | 2.07 | 2.61 | . |
| Divorced | 1.84 | 0.11 | 1.63 | 2.06 | . |
| Widowed | 1.83 | 0.18 | 1.48 | 2.18 | . |
| Domestic Partner | 1.95 | 0.06 | 1.83 | 2.07 | . |
| Employment |  |  |  |  |  |
| Employed for an Employer | 2.25 | 0.05 | 2.16 | 2.34 | 0.00 |
| Self-Employed | 2.40 | 0.06 | 2.29 | 2.52 | . |
| Retired | 2.21 | 0.10 | 2.01 | 2.41 | . |
| Student | 2.54 | 0.12 | 2.30 | 2.79 | . |
| Homemaker | 1.65 | 0.08 | 1.49 | 1.80 | . |
| Unemployed and Looking for a Job | 1.91 | 0.06 | 1.79 | 2.02 | . |
| None of These/Other | 1.91 | 0.14 | 1.63 | 2.19 | . |
| Education |  |  |  |  |  |
| Up to 8 Years | 2.03 | 0.06 | 1.90 | 2.15 | 0.00 |
| 9-15 Years | 2.13 | 0.03 | 2.07 | 2.20 | . |
| 16+ Years | 2.41 | 0.06 | 2.29 | 2.52 | . |
| Service Attendance |  |  |  |  |  |
| >1/Week | 2.41 | 0.07 | 2.27 | 2.53 | 0.00 |
| 1/Week | 2.40 | 0.07 | 2.11 | 2.44 | . |
| 1-3/Month | 2.27 | 0.08 | 2.00 | 2.19 | . |
| A Few Times a Year | 2.10 | 0.05 | 1.70 | 1.92 | . |
| Never | 1.81 | 0.06 | 2.27 | 2.53 | . |
| Immigration Status |  |  |  |  |  |
| Born in This Country | 0.91 | 0.00 | 2.10 | 2.21 | 0.084 |
| Born in Another Country | 0.85 | 0.04 | 2.09 | 3.05 | . |
| Religion |  |  |  |  |  |
| Christianity | 2.57 | 0.24 | 2.11 | 2.24 | 0.00 |
| Islam | 2.18 | 0.03 | 0.08 | 4.15 | . |
| Hinduism | 2.12 | 0.83 | -0.70 | 10.97 | . |
| Buddhism | 5.14 | 1.50 | 1.17 | 3.16 | . |
| Judaism | 2.17 | 0.49 | 0.86 | 3.29 | . |
| Sikhism | 2.07 | 0.59 | -2.61 | 12.63 | . |
| Baha'i | 5.01 | 1.56 | . | . | . |
| Jainism | 1.57 | . | . | . | . |
| Shinto | 3.90 | . |  |  | . |
| Taoism | 3.11 | 1.72 | -2.76 | 8.97 | . |
| Confucianism | 2.48 | 2.36 | -5.59 | 10.54 | . |
| Primal, Animist, or Folk Religion | 2.18 | 0.86 | 0.38 | 3.98 | . |
| Spiritism | 2.20 | 0.11 | 1.98 | 2.43 | . |
| African-Derived | 2.07 | 0.13 | 1.81 | 2.33 | . |
| Some Other Religion | 2.31 | 0.28 | 1.76 | 2.87 | . |
| No Religion/Atheist/Agnostic | 2.04 | 0.07 | 1.90 | 2.18 | . |
| Race/Ethnicity |  |  |  |  |  |
| Branca | 2.20 | 0.05 | 2.11 | 2.24 | 0.677 |
| Preta | 2.12 | 0.07 | 1.98 | 2.27 | . |
| Parda | 2.13 | 0.04 | 2.04 | 2.21 | . |
| Amarela | 2.31 | 0.19 | 1.93 | 2.68 | . |
| Indigena | 2.17 | 0.26 | 1.66 | 2.68 | . |
| Other | 1.91 | 0.29 | 1.33 | 2.50 | . |

**Table 4a: Nationally Representative Descriptive Statistics of the Observed Sample (Egypt)**

| Variable | Proportion | Frequency |
| --- | --- | --- |
| Age |  |  |
| 18-24 | 0.20 | 960 |
| 25-29 | 0.13 | 607 |
| 30-39 | 0.25 | 1204 |
| 40-49 | 0.19 | 897 |
| 50-59 | 0.13 | 613 |
| 60-69 | 0.08 | 387 |
| 70-79 | 0.01 | 54 |
| 80 or Older | 0.00 | 7 |
| Missing | . | . |
| Gender |  |  |
| Male | 0.51 | 2394 |
| Female | 0.49 | 2334 |
| Other | . | . |
| Missing | 0.00 | 0 |
| Marital Status |  |  |
| Single/Never Been Married | 0.20 | 947 |
| Married | 0.72 | 3387 |
| Separated | 0.01 | 39 |
| Divorced | 0.02 | 101 |
| Widowed | 0.05 | 238 |
| Domestic Partner | . | . |
| Missing | 0.00 | 17 |
| Employment |  |  |
| Employed for an Employer | 0.27 | 1267 |
| Self-Employed | 0.19 | 892 |
| Retired | 0.05 | 253 |
| Student | 0.06 | 297 |
| Homemaker | 0.37 | 1772 |
| Unemployed and Looking for a Job | 0.05 | 224 |
| None of These/Other | 0.00 | 21 |
| Missing | 0.00 | 3 |
| Education |  |  |
| Up to 8 Years | 0.53 | 2486 |
| 9-15 Years | 0.34 | 1599 |
| 16+ Years | 0.14 | 643 |
| Missing | 0.00 | 1 |
| Service Attendance |  |  |
| >1/Week | 0.18 | 839 |
| 1/Week | 0.20 | 960 |
| 1-3/Month | 0.08 | 368 |
| A Few Times a Year | 0.10 | 458 |
| Never | 0.44 | 2091 |
| Missing | 0.00 | 12 |
| Immigration Status |  |  |
| Born in This Country | 1.00 | 4713 |
| Born in Another Country | 0.00 | 16 |
| Missing | 0.00 | 1 |
| Religion |  |  |
| Christianity | 0.03 | 120 |
| Islam | 0.97 | 4607 |
| Hinduism | . | . |
| Buddhism | . | . |
| Judaism | . | . |
| Sikhism | . | . |
| Baha'i | . | . |
| Jainism | . | . |
| Shinto | . | . |
| Taoism | 0.00 | 0 |
| Confucianism | . | . |
| Primal, Animist, or Folk Religion | . | . |
| Spiritism | . | . |
| African-Derived | . | . |
| Chinese | . | . |
| Some Other Religion | . | . |
| No Religion/Atheist/Agnostic | . | . |
| Missing | 0.00 | 1 |
| Race/Ethnicity |  |  |
| Arab | 0.97 | 4585 |
| Turkish | 0.00 | 9 |
| Greek | 0.00 | 1 |
| Abazas | . | . |
| Bedouin Arab | 0.00 | 4 |
| Swiss | . | . |
| Nubian | 0.01 | 27 |
| Other | . | . |
| Missing | 0.02 | 102 |

**Table 4b: Variations Across Demographic Characteristics (Egypt)**

| Variable | Mean | SE | LCI | UCI | Global p-value |
| --- | --- | --- | --- | --- | --- |
| Age |  |  |  |  |  |
| 18-24 | 1.36 | 0.10 | 1.17 | 1.56 | 0.00 |
| 25-29 | 0.77 | 0.09 | 0.59 | 0.94 | . |
| 30-39 | 0.65 | 0.06 | 0.54 | 0.76 | . |
| 40-49 | 0.44 | 0.05 | 0.34 | 0.54 | . |
| 50-59 | 0.35 | 0.07 | 0.22 | 0.48 | . |
| 60-69 | 0.34 | 0.10 | 0.14 | 0.54 | . |
| 70-79 | 0.10 | 0.06 | -0.02 | 0.22 | . |
| 80 or Older | 0.00 | 0.00 | 0.00 | 0.00 | . |
| Gender |  |  |  |  |  |
| Male | 0.98 | 0.05 | 0.88 | 1.09 | 0.00 |
| Female | 0.41 | 0.03 | 0.36 | 0.46 | . |
| Other | . | . | . | . | . |
| Marital Status |  |  |  |  |  |
| Single/Never Been Married | 1.54 | 0.11 | 1.32 | 1.76 | 0.00 |
| Married | 0.50 | 0.03 | 0.44 | 0.55 | . |
| Separated | 0.75 | 0.13 | 0.49 | 1.02 | . |
| Divorced | 0.80 | 0.26 | 0.28 | 1.32 | . |
| Widowed | 0.20 | 0.09 | 0.03 | 0.37 | . |
| Domestic Partner | 0.50 | 0.03 | 0.44 | 0.55 | . |
| Employment |  |  |  |  |  |
| Employed for an Employer | 0.84 | 0.06 | 0.72 | 0.96 | 0.00 |
| Self-Employed | 0.79 | 0.08 | 0.65 | 0.94 | . |
| Retired | 0.39 | 0.11 | 0.17 | 0.62 | . |
| Student | 2.01 | 0.17 | 1.68 | 2.34 | . |
| Homemaker | 0.30 | 0.03 | 0.25 | 0.36 | . |
| Unemployed and Looking for a Job | 1.36 | 0.21 | 0.94 | 1.78 | . |
| None of These/Other | 0.08 | 0.01 | 0.06 | 0.09 | . |
| Education |  |  |  |  |  |
| Up to 8 Years | 0.36 | 0.03 | 0.30 | 0.43 | 0.00 |
| 9-15 Years | 1.05 | 0.06 | 0.93 | 1.17 | . |
| 16+ Years | 1.14 | 0.10 | 0.94 | 1.34 | . |
| Service Attendance |  |  |  |  |  |
| >1/Week | 1.07 | 0.10 | 0.87 | 1.26 | 0.00 |
| 1/Week | 0.74 | 0.07 | 0.60 | 0.87 | . |
| 1-3/Month | 0.90 | 0.11 | 0.68 | 1.12 | . |
| A Few Times a Year | 0.86 | 0.08 | 0.69 | 1.02 | . |
| Never | 0.46 | 0.04 | 0.39 | 0.54 | . |
| Immigration Status |  |  |  |  |  |
| Born in This Country | 0.70 | 0.03 | 0.64 | 0.99 | 0.374 |
| Born in Another Country | 0.49 | 0.10 | 0.27 | 0.72 | . |
| Religion |  |  |  |  |  |
| Christianity | 1.11 | 0.30 | 0.51 | 1.71 | 0.18 |
| Islam | 0.69 | 0.03 | 0.63 | 0.75 | . |
| Taoism | 0.00 | . | . | . | . |
| Race/Ethnicity |  |  |  |  |  |
| Arab | 0.70 | 0.03 | 0.98 | 0.99 | 0.00 |
| Turkish | 0.25 | 0.19 | . | . | . |
| Greek | 4.00 | . | . | . | . |
| Abazas | 0.00 | . | . | . | . |
| Bedouin Arab | 1.28 | 0.11 | . | . | . |
| Swiss | 0.70 | 0.03 | . | . | . |
| Nubian | 0.25 | 0.19 | . | . | . |

**Table 5a: Nationally Representative Descriptive Statistics of the Observed Sample (Germany)**

| Variable | Proportion | Frequency |
| --- | --- | --- |
| Age |  |  |
| 18-24 | 0.09 | 829 |
| 25-29 | 0.08 | 774 |
| 30-39 | 0.15 | 1438 |
| 40-49 | 0.16 | 1494 |
| 50-59 | 0.18 | 1729 |
| 60-69 | 0.20 | 1915 |
| 70-79 | 0.12 | 1137 |
| 80 or Older | 0.02 | 190 |
| Missing | . | . |
| Gender |  |  |
| Male | 0.49 | 4641 |
| Female | 0.51 | 4843 |
| Other | 0.00 | 11 |
| Missing | 0.00 | 11 |
| Marital Status |  |  |
| Single/Never Been Married | 0.28 | 2627 |
| Married | 0.50 | 4784 |
| Separated | 0.02 | 219 |
| Divorced | 0.08 | 767 |
| Widowed | 0.04 | 409 |
| Domestic Partner | 0.07 | 619 |
| Missing | 0.01 | 81 |
| Employment |  |  |
| Employed for an Employer | 0.52 | 4950 |
| Self-Employed | 0.07 | 712 |
| Retired | 0.26 | 2480 |
| Student | 0.06 | 605 |
| Homemaker | 0.03 | 251 |
| Unemployed and Looking for a Job | 0.03 | 288 |
| None of These/Other | 0.02 | 204 |
| Missing | 0.00 | 14 |
| Education |  |  |
| Up to 8 Years | 0.02 | 235 |
| 9-15 Years | 0.64 | 6094 |
| 16+ Years | 0.33 | 3164 |
| Missing | 0.00 | 13 |
| Service Attendance |  |  |
| >1/Week | 0.03 | 285 |
| 1/Week | 0.04 | 424 |
| 1-3/Month | 0.06 | 550 |
| A Few Times a Year | 0.25 | 2362 |
| Never | 0.62 | 5876 |
| Missing | 0.00 | 9 |
| Immigration Status |  |  |
| Born in This Country | 0.92 | 8722 |
| Born in Another Country | 0.08 | 744 |
| Missing | 0.00 | 40 |
| Religion |  |  |
| Christianity | 0.53 | 5052 |
| Islam | 0.04 | 351 |
| Hinduism | 0.00 | 12 |
| Buddhism | 0.01 | 51 |
| Judaism | 0.00 | 19 |
| Sikhism | 0.00 | 5 |
| Baha'i | 0.00 | 3 |
| Jainism | . | . |
| Shinto | 0.00 | 2 |
| Taoism | 0.00 | 0 |
| Confucianism | 0.00 | 4 |
| Primal, Animist, or Folk Religion | 0.00 | 34 |
| Spiritism | . | . |
| African-Derived | . | . |
| Chinese | . | . |
| Some Other Religion | 0.01 | 60 |
| No Religion/Atheist/Agnostic | 0.40 | 3815 |
| Missing | 0.01 | 99 |
| Race/Ethnicity |  |  |
| No Data | . | . |

**Table 5b: Variations Across Demographic Characteristics (Germany)**

| Variable | Mean | SE | LCI | UCI | Global p-value |
| --- | --- | --- | --- | --- | --- |
| Age |  |  |  |  |  |
| 18-24 | 2.65 | 0.10 | 2.44 | 2.85 | 0.00 |
| 25-29 | 2.51 | 0.09 | 2.33 | 2.68 | . |
| 30-39 | 2.55 | 0.06 | 2.42 | 2.67 | . |
| 40-49 | 2.44 | 0.07 | 2.30 | 2.58 | . |
| 50-59 | 2.31 | 0.07 | 2.18 | 2.45 | . |
| 60-69 | 2.28 | 0.06 | 2.16 | 2.41 | . |
| 70-79 | 2.11 | 0.09 | 1.94 | 2.28 | . |
| 80 or Older | 2.08 | 0.22 | 1.65 | 2.52 | . |
| Gender |  |  |  |  |  |
| Male | 2.46 | 0.04 | 2.38 | 2.54 | 0.017 |
| Female | 2.31 | 0.04 | 2.23 | 2.39 | . |
| Other | 2.46 | 0.04 | 1.32 | 2.49 | . |
| Marital Status |  |  |  |  |  |
| Single/Never Been Married | 2.38 | 0.05 | 2.28 | 2.49 | 0.037 |
| Married | 2.41 | 0.04 | 2.33 | 2.49 | . |
| Separated | 2.51 | 0.18 | 2.15 | 2.87 | . |
| Divorced | 2.24 | 0.10 | 2.04 | 2.44 | . |
| Widowed | 2.00 | 0.14 | 1.72 | 2.27 | . |
| Domestic Partner | 2.50 | 0.11 | 2.28 | 2.71 | . |
| Employment |  |  |  |  |  |
| Employed for an Employer | 2.47 | 0.04 | 2.39 | 2.54 | 0.00 |
| Self-Employed | 2.56 | 0.10 | 2.37 | 2.76 | . |
| Retired | 2.21 | 0.06 | 2.10 | 2.33 | . |
| Student | 2.65 | 0.12 | 2.42 | 2.89 | . |
| Homemaker | 2.12 | 0.16 | 1.80 | 2.44 | . |
| Unemployed and Looking for a Job | 1.86 | 0.15 | 1.56 | 2.15 | . |
| None of These/Other | 1.93 | 0.17 | 1.61 | 2.26 | . |
| Education |  |  |  |  |  |
| Up to 8 Years | 1.93 | 0.17 | 1.59 | 2.26 | 0.00 |
| 9-15 Years | 2.28 | 0.04 | 2.21 | 2.35 | . |
| 16+ Years | 2.60 | 0.05 | 2.51 | 2.69 | . |
| Service Attendance |  |  |  |  |  |
| >1/Week | 2.48 | 0.18 | 2.13 | 2.82 | 0.00 |
| 1/Week | 2.69 | 0.13 | 2.43 | 2.95 | . |
| 1-3/Month | 2.87 | 0.12 | 2.64 | 3.11 | . |
| A Few Times a Year | 2.48 | 0.06 | 2.36 | 2.59 | . |
| Never | 2.27 | 0.04 | 2.20 | 2.34 | . |
| Immigration Status |  |  |  |  |  |
| Born in This Country | 2.37 | 0.03 | 2.31 | 2.43 | 0.335 |
| Born in Another Country | 2.48 | 0.11 | 2.27 | 2.69 | . |
| Religion |  |  |  |  |  |
| Christianity | 2.41 | 0.04 | 2.33 | 2.48 | 0.00 |
| Islam | 2.60 | 0.17 | 2.28 | 2.93 | . |
| Hinduism | 2.10 | 0.77 | 0.18 | 4.02 | . |
| Buddhism | 2.18 | 0.32 | 1.54 | 2.82 | . |
| Judaism | 2.46 | 0.54 | 1.31 | 3.60 | . |
| Sikhism | 2.36 | 0.17 | 1.79 | 2.93 | . |
| Baha'i | 3.78 | 1.34 | -9.53 | 17.10 | . |
| Jainism | 1.00 | . | . | . | . |
| Shinto | 5.00 | . | . | . | . |
| Taoism | 1.64 | . | . | . | . |
| Confucianism | 2.88 | 0.51 | 1.83 | 3.94 | . |
| Primal, Animist, or Folk Religion | 2.20 | 0.50 | 1.19 | 3.20 | . |
| Some Other Religion | 2.33 | 0.05 | 2.24 | 2.42 | . |
| No Religion/Atheist/Agnostic | 2.41 | 0.04 | 2.33 | 2.48 | . |
| Race/Ethnicity |  |  |  |  |  |
| No Data | . | . | . | . | . |

**Table 6a: Nationally Representative Descriptive Statistics of the Observed Sample (Hong_Kong)**

| Variable | Proportion | Frequency |
| --- | --- | --- |
| Age |  |  |
| 18-24 | 0.07 | 217 |
| 25-29 | 0.07 | 198 |
| 30-39 | 0.17 | 507 |
| 40-49 | 0.19 | 580 |
| 50-59 | 0.24 | 711 |
| 60-69 | 0.21 | 620 |
| 70-79 | 0.05 | 164 |
| 80 or Older | 0.00 | 15 |
| Missing | . | . |
| Gender |  |  |
| Male | 0.46 | 1390 |
| Female | 0.54 | 1620 |
| Other | 0.00 | 2 |
| Missing | . | . |
| Marital Status |  |  |
| Single/Never Been Married | 0.24 | 723 |
| Married | 0.69 | 2080 |
| Separated | 0.01 | 21 |
| Divorced | 0.03 | 105 |
| Widowed | 0.01 | 45 |
| Domestic Partner | 0.01 | 37 |
| Missing | 0.00 | 1 |
| Employment |  |  |
| Employed for an Employer | 0.68 | 2056 |
| Self-Employed | 0.08 | 245 |
| Retired | 0.14 | 423 |
| Student | 0.02 | 55 |
| Homemaker | 0.04 | 114 |
| Unemployed and Looking for a Job | 0.02 | 62 |
| None of These/Other | 0.01 | 39 |
| Missing | 0.01 | 18 |
| Education |  |  |
| Up to 8 Years | 0.14 | 433 |
| 9-15 Years | 0.67 | 2031 |
| 16+ Years | 0.18 | 547 |
| Missing | . | . |
| Service Attendance |  |  |
| >1/Week | 0.08 | 237 |
| 1/Week | 0.19 | 567 |
| 1-3/Month | 0.11 | 332 |
| A Few Times a Year | 0.18 | 543 |
| Never | 0.44 | 1332 |
| Missing | 0.00 | 1 |
| Immigration Status |  |  |
| Born in This Country | 0.88 | 2637 |
| Born in Another Country | 0.11 | 321 |
| Missing | 0.02 | 53 |
| Religion |  |  |
| Christianity | 0.25 | 757 |
| Islam | 0.03 | 86 |
| Hinduism | 0.01 | 20 |
| Buddhism | 0.12 | 349 |
| Judaism | 0.00 | 10 |
| Sikhism | 0.00 | 2 |
| Baha'i | 0.00 | 3 |
| Jainism | 0.00 | 1 |
| Shinto | 0.01 | 19 |
| Taoism | 0.03 | 97 |
| Confucianism | 0.00 | 11 |
| Primal, Animist, or Folk Religion | 0.01 | 27 |
| Spiritism | . | . |
| African-Derived | . | . |
| Chinese | 0.04 | 106 |
| Some Other Religion | 0.00 | 4 |
| No Religion/Atheist/Agnostic | 0.50 | 1518 |
| Missing | 0.00 | 5 |
| Race/Ethnicity |  |  |
| Chinese (Cantonese) | 0.64 | 1930 |
| Chinese (Chaoshan) | 0.07 | 201 |
| Chinese (Fujianese) | 0.04 | 117 |
| Chinese (Hakka) | 0.04 | 121 |
| Chinese (Shanghainese) | 0.03 | 89 |
| Chinese (Other Ethnicity) | 0.09 | 264 |
| East Asian (Korean, Japanese) | 0.00 | 10 |
| Southeast Asian (Filipino, Indonesian, Thailand) | 0.02 | 46 |
| South Asian (Indian, Nepalese, Pakistani) | 0.01 | 17 |
| Taiwanese | 0.00 | 14 |
| White | 0.00 | 15 |
| Other | 0.00 | 4 |
| Missing | 0.06 | 184 |

**Table 6b: Variations Across Demographic Characteristics (Hong_Kong)**

| Variable | Mean | SE | LCI | UCI | Global p-value |
| --- | --- | --- | --- | --- | --- |
| Age |  |  |  |  |  |
| 18-24 | 2.41 | 0.12 | 2.17 | 2.65 | 0.00 |
| 25-29 | 1.77 | 0.14 | 1.50 | 2.04 | . |
| 30-39 | 2.03 | 0.10 | 1.83 | 2.23 | . |
| 40-49 | 2.10 | 0.10 | 1.90 | 2.30 | . |
| 50-59 | 2.46 | 0.09 | 2.28 | 2.64 | . |
| 60-69 | 2.31 | 0.17 | 1.98 | 2.64 | . |
| 70-79 | 2.41 | 0.49 | 1.42 | 3.40 | . |
| 80 or Older | 2.30 | 0.31 | 0.80 | 3.79 | . |
| Gender |  |  |  |  |  |
| Male | 2.47 | 0.08 | 2.30 | 2.63 | 0.00 |
| Female | 2.04 | 0.08 | 1.89 | 2.19 | . |
| Other | 1.64 | . | . | . | . |
| Marital Status |  |  |  |  |  |
| Single/Never Been Married | 1.78 | 0.09 | 1.59 | 1.96 | 0.00 |
| Married | 2.43 | 0.07 | 2.30 | 2.57 | . |
| Separated | 2.72 | 1.15 | -0.06 | 5.50 | . |
| Divorced | 1.91 | 0.32 | 1.25 | 2.57 | . |
| Widowed | 1.33 | 0.69 | -0.12 | 2.77 | . |
| Domestic Partner | 1.95 | 0.43 | 1.08 | 2.81 | . |
| Employment |  |  |  |  |  |
| Employed for an Employer | 2.30 | 0.05 | 2.19 | 2.41 | 0.024 |
| Self-Employed | 2.60 | 0.16 | 2.28 | 2.91 | . |
| Retired | 2.07 | 0.25 | 1.57 | 2.57 | . |
| Student | 2.05 | 0.28 | 1.49 | 2.60 | . |
| Homemaker | 1.54 | 0.35 | 0.84 | 2.24 | . |
| Unemployed and Looking for a Job | 1.64 | 0.35 | 0.94 | 2.34 | . |
| None of These/Other | 1.82 | 0.76 | 0.25 | 3.39 | . |
| Education |  |  |  |  |  |
| Up to 8 Years | 2.15 | 0.20 | 1.75 | 2.54 | 0.664 |
| 9-15 Years | 2.23 | 0.06 | 2.11 | 2.35 | . |
| 16+ Years | 2.33 | 0.13 | 2.08 | 2.59 | . |
| Service Attendance |  |  |  |  |  |
| >1/Week | 2.84 | 0.17 | 2.50 | 3.19 | 0.00 |
| 1/Week | 2.49 | 0.13 | 2.22 | 2.75 | . |
| 1-3/Month | 2.53 | 0.16 | 2.21 | 2.85 | . |
| A Few Times a Year | 2.46 | 0.15 | 2.18 | 2.75 | . |
| Never | 1.86 | 0.08 | 1.70 | 2.02 | . |
| Immigration Status |  |  |  |  |  |
| Born in This Country | 2.25 | 0.06 | 2.14 | 2.36 | 0.538 |
| Born in Another Country | 2.10 | 0.25 | 1.61 | 2.60 | . |
| Religion |  |  |  |  |  |
| Christianity | 2.50 | 0.12 | 2.26 | 2.74 | 0.00 |
| Islam | 2.32 | 0.49 | 1.34 | 3.31 | . |
| Hinduism | 1.92 | 0.34 | 1.19 | 2.65 | . |
| Buddhism | 2.54 | 0.14 | 2.27 | 2.81 | . |
| Judaism | 2.65 | 0.25 | 2.09 | 3.21 | . |
| Sikhism | 5.26 | . | . | . | . |
| Baha'i | 1.27 | 0.97 | -1.57 | 4.10 | . |
| Jainism | 0.00 | . | . | . | . |
| Shinto | 1.76 | 0.43 | 0.77 | 2.76 | . |
| Taoism | 2.79 | 0.34 | 2.12 | 3.46 | . |
| Confucianism | 2.96 | 0.70 | 1.42 | 4.50 | . |
| Primal, Animist, or Folk Religion | 2.19 | 0.48 | 1.22 | 3.17 | . |
| Chinese folk/traditional religion | 1.75 | 0.24 | 1.26 | 2.23 | . |
| Some Other Religion | 3.09 | 2.73 | -9.98 | 16.15 | . |
| No Religion/Atheist/Agnostic | 2.03 | 0.08 | 1.88 | 2.18 | . |
| Race/Ethnicity |  |  |  |  |  |
| Chinese (Cantonese) | 2.33 | 0.07 | 2.19 | 2.46 | 0.00 |
| Chinese (Chaoshan) | 2.17 | 0.16 | 1.84 | 2.49 | . |
| Chinese (Fujianese) | 1.85 | 0.15 | 1.56 | 2.14 | . |
| Chinese (Hakka) | 1.65 | 0.18 | 1.30 | 2.00 | . |
| Chinese (Shanghainese) | 2.06 | 0.49 | 1.09 | 3.03 | . |
| Chinese (Other Ethnicity) | 2.22 | 0.17 | 1.89 | 2.55 | . |
| East Asian (Korean, Japanese) | 2.33 | 0.60 | 0.73 | 3.94 | . |
| Southeast Asian (Filipino, Indonesian, Thailand) | 2.01 | 0.71 | 0.51 | 3.51 | . |
| South Asian (Indian, Nepalese, Pakistani) | 1.07 | 0.39 | 0.23 | 1.92 | . |
| Taiwanese | 1.65 | 0.79 | -0.05 | 3.34 | . |
| White | 3.52 | 1.26 | 0.77 | 6.27 | . |
| Other | 2.59 | 0.68 | 0.54 | 4.63 | . |

**Table 7a: Nationally Representative Descriptive Statistics of the Observed Sample (India)**

| Variable | Proportion | Frequency |
| --- | --- | --- |
| Age |  |  |
| 18-24 | 0.20 | 2543 |
| 25-29 | 0.13 | 1640 |
| 30-39 | 0.24 | 3109 |
| 40-49 | 0.18 | 2275 |
| 50-59 | 0.12 | 1574 |
| 60-69 | 0.09 | 1188 |
| 70-79 | 0.03 | 370 |
| 80 or Older | 0.01 | 67 |
| Missing | . | . |
| Gender |  |  |
| Male | 0.51 | 6473 |
| Female | 0.49 | 6292 |
| Other | . | . |
| Missing | . | . |
| Marital Status |  |  |
| Single/Never Been Married | 0.16 | 2065 |
| Married | 0.77 | 9848 |
| Separated | 0.00 | 45 |
| Divorced | 0.00 | 25 |
| Widowed | 0.03 | 445 |
| Domestic Partner | 0.02 | 269 |
| Missing | 0.01 | 69 |
| Employment |  |  |
| Employed for an Employer | 0.21 | 2660 |
| Self-Employed | 0.27 | 3401 |
| Retired | 0.02 | 286 |
| Student | 0.04 | 532 |
| Homemaker | 0.33 | 4221 |
| Unemployed and Looking for a Job | 0.07 | 902 |
| None of These/Other | 0.06 | 715 |
| Missing | 0.00 | 48 |
| Education |  |  |
| Up to 8 Years | 0.89 | 11422 |
| 9-15 Years | 0.09 | 1194 |
| 16+ Years | 0.01 | 145 |
| Missing | 0.00 | 4 |
| Service Attendance |  |  |
| >1/Week | 0.23 | 2875 |
| 1/Week | 0.25 | 3166 |
| 1-3/Month | 0.21 | 2740 |
| A Few Times a Year | 0.16 | 2090 |
| Never | 0.14 | 1823 |
| Missing | 0.01 | 71 |
| Immigration Status |  |  |
| Born in This Country | 0.99 | 12629 |
| Born in Another Country | 0.01 | 110 |
| Missing | 0.00 | 26 |
| Religion |  |  |
| Christianity | 0.02 | 306 |
| Islam | 0.12 | 1555 |
| Hinduism | 0.81 | 10362 |
| Buddhism | 0.02 | 230 |
| Judaism | . | . |
| Sikhism | 0.01 | 127 |
| Baha'i | . | . |
| Jainism | 0.00 | 10 |
| Shinto | 0.00 | 1 |
| Taoism | . | . |
| Confucianism | . | . |
| Primal, Animist, or Folk Religion | 0.00 | 30 |
| Spiritism | . | . |
| African-Derived | . | . |
| Chinese | . | . |
| Some Other Religion | 0.01 | 67 |
| No Religion/Atheist/Agnostic | 0.00 | 13 |
| Missing | 0.00 | 62 |
| Race/Ethnicity |  |  |
| General | 0.28 | 3538 |
| Other Backward Caste | 0.33 | 4177 |
| Schedule Caste | 0.28 | 3599 |
| Schedule Tribe | 0.09 | 1185 |
| Other | . | . |
| Missing | 0.02 | 267 |

**Table 7b: Variations Across Demographic Characteristics (India)**

| Variable | Mean | SE | LCI | UCI | Global p-value |
| --- | --- | --- | --- | --- | --- |
| Age |  |  |  |  |  |
| 18-24 | 3.11 | 0.09 | 2.94 | 3.29 | 0.302 |
| 25-29 | 3.12 | 0.10 | 2.92 | 3.32 | . |
| 30-39 | 3.03 | 0.07 | 2.89 | 3.16 | . |
| 40-49 | 3.20 | 0.09 | 3.01 | 3.39 | . |
| 50-59 | 3.20 | 0.10 | 3.00 | 3.41 | . |
| 60-69 | 3.22 | 0.14 | 2.95 | 3.48 | . |
| 70-79 | 2.86 | 0.26 | 2.34 | 3.39 | . |
| 80 or Older | 3.71 | 0.45 | 2.80 | 4.62 | . |
| Gender |  |  |  |  |  |
| Male | 3.54 | 0.06 | 3.42 | 3.67 | 0.00 |
| Female | 2.70 | 0.06 | 2.58 | 2.81 | . |
| Other | . | . | . | . | . |
| Marital Status |  |  |  |  |  |
| Single/Never Been Married | 3.51 | 0.10 | (3.30 | 3.71 | 0.00 |
| Married | 3.05 | 0.04 | 2.96 | 3.14 | . |
| Separated | 3.17 | 0.57 | 2.00 | 4.34 | . |
| Divorced | 2.31 | 0.40 | 1.44 | 3.18 | . |
| Widowed | 2.54 | 0.22 | 2.09 | 2.99 | . |
| Domestic Partner | 3.96 | 0.29 | 3.38 | 4.55 | . |
| Employment |  |  |  |  |  |
| Employed for an Employer | 3.40 | 0.09 | 3.23 | 3.56 | 0.00 |
| Self-Employed | 3.20 | 0.09 | 3.02 | 3.38 | . |
| Retired | 3.12 | 0.27 | 2.56 | 3.67 | . |
| Student | 3.66 | 0.19 | 3.26 | 4.06 | . |
| Homemaker | 2.74 | 0.07 | 2.61 | 2.87 | . |
| Unemployed and Looking for a Job | 3.68 | 0.14 | 3.40 | 3.96 | . |
| None of These/Other | 2.92 | 0.17 | 2.58 | 3.27 | . |
| Education |  |  |  |  |  |
| Up to 8 Years | 3.07 | 0.04 | 2.98 | 3.16 | 0.00 |
| 9-15 Years | 3.53 | 0.11 | 3.31 | 3.74 | . |
| 16+ Years | 4.29 | 0.28 | 3.73 | 4.85 | . |
| Service Attendance |  |  |  |  |  |
| >1/Week | 3.68 | 0.08 | 3.52 | 3.84 | 0.00 |
| 1/Week | 3.17 | 0.07 | 3.02 | 3.31 | . |
| 1-3/Month | 3.25 | 0.09 | 3.07 | 3.43 | . |
| A Few Times a Year | 2.63 | 0.10 | 2.43 | 2.84 | . |
| Never | 2.55 | 0.11 | 2.33 | 2.76 | . |
| Immigration Status |  |  |  |  |  |
| Born in This Country | 3.12 | 0.04 | 3.04 | 3.21 | 0.648 |
| Born in Another Country | 3.19 | 0.47 | 2.25 | 4.13 | . |
| Religion |  |  |  |  |  |
| Christianity | 3.20 | 0.28 | 2.63 | 3.78 | 0.00 |
| Islam | 2.58 | 0.14 | 2.30 | 2.85 | . |
| Hinduism | 3.19 | 0.05 | 3.09 | 3.28 | . |
| Buddhism | 3.47 | 0.26 | 2.96 | 3.98 | . |
| Judaism | 4.09 | 0.44 | 3.15 | 5.02 | . |
| Sikhism | 3.52 | 0.55 | 2.27 | 4.76 | . |
| Jainism | 7.00 | . | . | . | . |
| Shinto | 2.50 | 0.58 | 1.31 | 3.68 | . |
| Primal, Animist, or Folk Religion | 3.10 | 0.32 | 2.41 | 3.78 | . |
| Some Other Religion | 2.84 | . | . | . | . |
| No Religion/Atheist/Agnostic | 2.58 | 0.14 | 2.30 | 2.85 | . |
| Race/Ethnicity |  |  |  |  |  |
| General | 2.95 | 0.08 | 2.78 | 3.12 | 0.046 |
| Other Backward Caste | 3.22 | 0.08 | 3.06 | 3.38 |  |
| Schedule Caste | 3.18 | 0.08 | 3.02 | 3.33 |  |
| Schedule Tribe | 3.15 | 0.12 | 2.92 | 3.39 |  |

**Table 8a: Nationally Representative Descriptive Statistics of the Observed Sample (Indonesia)**

| Variable | Proportion | Frequency |
| --- | --- | --- |
| Age |  |  |
| 18-24 | 0.17 | 1216 |
| 25-29 | 0.12 | 849 |
| 30-39 | 0.23 | 1591 |
| 40-49 | 0.23 | 1576 |
| 50-59 | 0.17 | 1169 |
| 60-69 | 0.07 | 490 |
| 70-79 | 0.01 | 83 |
| 80 or Older | 0.00 | 17 |
| Missing | . | . |
| Gender |  |  |
| Male | 0.50 | 3461 |
| Female | 0.50 | 3513 |
| Other | 0.00 | 7 |
| Missing | 0.00 | 11 |
| Marital Status |  |  |
| Single/Never Been Married | 0.20 | 1381 |
| Married | 0.69 | 4846 |
| Separated | 0.01 | 82 |
| Divorced | 0.03 | 196 |
| Widowed | 0.06 | 425 |
| Domestic Partner | 0.00 | 18 |
| Missing | 0.01 | 45 |
| Employment |  |  |
| Employed for an Employer | 0.19 | 1323 |
| Self-Employed | 0.31 | 2187 |
| Retired | 0.01 | 78 |
| Student | 0.04 | 272 |
| Homemaker | 0.31 | 2138 |
| Unemployed and Looking for a Job | 0.08 | 529 |
| None of These/Other | 0.06 | 448 |
| Missing | 0.00 | 18 |
| Education |  |  |
| Up to 8 Years | 0.44 | 3079 |
| 9-15 Years | 0.50 | 3491 |
| 16+ Years | 0.06 | 419 |
| Missing | 0.00 | 2 |
| Service Attendance |  |  |
| >1/Week | 0.38 | 2667 |
| 1/Week | 0.36 | 2529 |
| 1-3/Month | 0.11 | 786 |
| A Few Times a Year | 0.09 | 659 |
| Never | 0.05 | 332 |
| Missing | 0.00 | 18 |
| Immigration Status |  |  |
| Born in This Country | 1.00 | 6958 |
| Born in Another Country | 0.00 | 34 |
| Missing | . | . |
| Religion |  |  |
| Christianity | 0.07 | 504 |
| Islam | 0.92 | 6406 |
| Hinduism | 0.01 | 73 |
| Buddhism | 0.00 | 3 |
| Judaism | . | . |
| Sikhism | . | . |
| Baha'i | . | . |
| Jainism | . | . |
| Shinto | . | . |
| Taoism | 0.00 | 1 |
| Confucianism | . | . |
| Primal, Animist, or Folk Religion | . | . |
| Spiritism | . | . |
| African-Derived | . | . |
| Chinese | . | . |
| Some Other Religion | 0.00 | 1 |
| No Religion/Atheist/Agnostic | . | . |
| Missing | 0.00 | 4 |
| Race/Ethnicity |  |  |
| Banjar/Melayu Banjar | 0.05 | 320 |
| Betawi | 0.04 | 251 |
| Bugis | 0.03 | 243 |
| Jawa | 0.41 | 2846 |
| Madura | 0.04 | 262 |
| Minangkabau | 0.04 | 273 |
| Sunda/Parahyangan | 0.17 | 1172 |
| Bali | 0.01 | 69 |
| Batak | 0.02 | 165 |
| Makasar | 0.01 | 91 |
| Other | 0.18 | 1262 |
| Missing | 0.01 | 38 |

**Table 8b: Variations Across Demographic Characteristics (Indonesia)**

| Variable | Mean | SE | LCI | UCI | Global p-value |
| --- | --- | --- | --- | --- | --- |
| Age |  |  |  |  |  |
| 18-24 | 2.59 | 0.09 | 2.43 | 2.76 | 0.00 |
| 25-29 | 2.93 | 0.11 | 2.72 | 3.14 | . |
| 30-39 | 2.91 | 0.09 | 2.74 | 3.08 | . |
| 40-49 | 2.88 | 0.10 | 2.69 | 3.08 | . |
| 50-59 | 3.12 | 0.12 | 2.89 | 3.35 | . |
| 60-69 | 3.74 | 0.22 | 3.30 | 4.17 | . |
| 70-79 | 3.59 | 0.51 | 2.56 | 4.62 | . |
| 80 or Older | 4.12 | 1.08 | 1.42 | 6.83 | . |
| Gender |  |  |  |  |  |
| Male | 3.22 | 0.07 | 3.07 | 3.36 | 0.00 |
| Female | 2.70 | 0.06 | 2.58 | 2.83 | . |
| Other | 2.09 | . | . | . | . |
| Marital Status |  |  |  |  |  |
| Single/Never Been Married | 2.69 | 0.08 | 2.53 | 2.85 | 0.004 |
| Married | 2.98 | 0.06 | 2.86 | 3.10 | . |
| Separated | 2.70 | 0.32 | 2.06 | 3.34 | . |
| Divorced | 3.22 | 0.30 | 2.64 | 3.80 | . |
| Widowed | 3.40 | 0.20 | 3.01 | 3.78 | . |
| Domestic Partner | 3.53 | 0.87 | 1.67 | 5.38 | . |
| Employment |  |  |  |  |  |
| Employed for an Employer | 2.88 | 0.11 | 2.67 | 3.09 | 0.00 |
| Self-Employed | 3.15 | 0.09 | 2.97 | 3.32 | . |
| Retired | 4.22 | 0.42 | 3.37 | 5.06 | . |
| Student | 2.18 | 0.13 | 1.92 | 2.44 | . |
| Homemaker | 2.88 | 0.08 | 2.72 | 3.05 | . |
| Unemployed and Looking for a Job | 2.85 | 0.14 | 2.58 | 3.12 | . |
| None of These/Other | 2.96 | 0.21 | 2.55 | 3.37 | . |
| Education |  |  |  |  |  |
| Up to 8 Years | 3.21 | 0.09 | 3.03 | 3.39 | 0.00 |
| 9-15 Years | 2.76 | 0.05 | 2.66 | 2.86 | . |
| 16+ Years | 2.74 | 0.11 | 2.52 | 2.95 | . |
| Service Attendance | 3.14 | 0.07 | 2.99 | 3.28 |  |
| >1/Week | 2.99 | 0.07 | 2.84 | 3.13 | 0.00 |
| 1/Week | 2.68 | 0.12 | 2.44 | 2.92 | . |
| 1-3/Month | 2.52 | 0.14 | 2.24 | 2.80 | . |
| A Few Times a Year | 2.75 | 0.23 | 2.30 | 3.19 | . |
| Never | 3.14 | 0.07 | 2.99 | 3.28 | . |
| Immigration Status |  |  |  |  |  |
| Born in This Country | 2.95 | 0.05 | 2.85 | 3.05 | 0.119 |
| Born in Another Country | 3.80 | 0.22 | 3.33 | 4.27 | . |
| Religion |  |  |  |  |  |
| Christianity | 3.12 | 0.19 | 2.74 | 3.50 | 0.00 |
| Islam | 2.95 | 0.05 | 2.84 | 3.05 | . |
| Hinduism | 2.66 | 0.42 | 1.83 | 3.50 | . |
| Buddhism | 0.75 | . | . | . | . |
| Taoism | 7.00 | . | . | . | . |
| Some Other Religion | 0.00 | . | . | . | . |
| Race/Ethnicity |  |  |  |  |  |
| Banjar/Melayu Banjar | 2.61 | 0.21 | 2.20 | 3.02 | 0.061 |
| Betawi | 2.57 | 0.26 | 2.05 | 3.09 | . |
| Bugis | 2.49 | 0.20 | 2.10 | 2.88 | . |
| Jawa | 2.97 | 0.07 | 2.85 | 3.10 | . |
| Madura | 2.90 | 0.22 | 2.45 | 3.34 | . |
| Minangkabau | 2.88 | 0.20 | 2.49 | 3.28 | . |
| Sunda/Parahyangan | 3.05 | 0.15 | 2.76 | 3.33 | . |
| Bali | 2.65 | 0.45 | 1.74 | 3.55 | . |
| Batak | 2.66 | 0.24 | 2.19 | 3.12 | . |
| Makasar | 2.93 | 0.24 | 2.45 | 3.41 | . |
| Other | 3.17 | 0.12 | 2.93 | 3.41 | . |

**Table 9a: Nationally Representative Descriptive Statistics of the Observed Sample (Israel)**

| Variable | Proportion | Frequency |
| --- | --- | --- |
| Age |  |  |
| 18-24 | 0.15 | 553 |
| 25-29 | 0.11 | 407 |
| 30-39 | 0.18 | 666 |
| 40-49 | 0.17 | 616 |
| 50-59 | 0.15 | 542 |
| 60-69 | 0.13 | 469 |
| 70-79 | 0.09 | 336 |
| 80 or Older | 0.02 | 79 |
| Missing | . | . |
| Gender |  |  |
| Male | 0.49 | 1791 |
| Female | 0.51 | 1872 |
| Other | 0.00 | 0 |
| Missing | 0.00 | 6 |
| Marital Status |  |  |
| Single/Never Been Married | 0.23 | 834 |
| Married | 0.56 | 2056 |
| Separated | 0.01 | 48 |
| Divorced | 0.07 | 258 |
| Widowed | 0.06 | 212 |
| Domestic Partner | 0.05 | 193 |
| Missing | 0.02 | 69 |
| Employment |  |  |
| Employed for an Employer | 0.49 | 1793 |
| Self-Employed | 0.12 | 424 |
| Retired | 0.16 | 576 |
| Student | 0.11 | 388 |
| Homemaker | 0.06 | 211 |
| Unemployed and Looking for a Job | 0.04 | 148 |
| None of These/Other | 0.03 | 118 |
| Missing | 0.00 | 10 |
| Education |  |  |
| Up to 8 Years | 0.06 | 224 |
| 9-15 Years | 0.41 | 1517 |
| 16+ Years | 0.52 | 1926 |
| Missing | 0.00 | 2 |
| Service Attendance |  |  |
| >1/Week | 0.18 | 649 |
| 1/Week | 0.14 | 495 |
| 1-3/Month | 0.10 | 374 |
| A Few Times a Year | 0.28 | 1014 |
| Never | 0.31 | 1122 |
| Missing | 0.00 | 14 |
| Immigration Status |  |  |
| Born in This Country | 0.76 | 2796 |
| Born in Another Country | 0.24 | 868 |
| Missing | 0.00 | 5 |
| Religion |  |  |
| Christianity | 0.01 | 39 |
| Islam | 0.18 | 656 |
| Hinduism | . | . |
| Buddhism | . | . |
| Judaism | 0.79 | 2897 |
| Sikhism | . | . |
| Baha'i | 0.00 | 2 |
| Jainism | . | . |
| Shinto | . | . |
| Taoism | 0.00 | 1 |
| Confucianism | . | . |
| Primal, Animist, or Folk Religion | 0.00 | 1 |
| Spiritism | . | . |
| African-Derived | . | . |
| Chinese | . | . |
| Some Other Religion | 0.00 | 5 |
| No Religion/Atheist/Agnostic | 0.02 | 64 |
| Missing | 0.00 | 4 |
| Race/Ethnicity |  |  |
| Jewish | 0.80 | 2926 |
| Arab | 0.18 | 674 |
| Other | 0.01 | 39 |
| Missing | 0.01 | 30 |

**Table 9b: Variations Across Demographic Characteristics (Israel)**

| Variable | Mean | SE | LCI | UCI | Global p-value |
| --- | --- | --- | --- | --- | --- |
| Age |  |  |  |  |  |
| 18-24 | 1.55 | 0.09 | 1.38 | 1.73 | 0.00 |
| 25-29 | 1.53 | 0.08 | 1.37 | 1.69 | . |
| 30-39 | 1.46 | 0.09 | 1.29 | 1.63 | . |
| 40-49 | 1.22 | 0.10 | 1.02 | 1.41 | . |
| 50-59 | 1.14 | 0.08 | 0.98 | 1.29 | . |
| 60-69 | 0.98 | 0.12 | 0.74 | 1.22 | . |
| 70-79 | 1.06 | 0.14 | 0.79 | 1.34 | . |
| 80 or Older | 1.04 | 0.17 | 0.70 | 1.38 | . |
| Gender |  |  |  |  |  |
| Male | 1.37 | 0.07 | 1.23 | 1.51 | 0.00 |
| Female | 1.21 | 0.06 | 1.09 | 1.33 | . |
| Other | 0.00 | . | . | . | . |
| Marital Status |  |  |  |  |  |
| Single/Never Been Married | 1.60 | 0.07 | 1.47 | 1.74 | 0.00 |
| Married | 1.16 | 0.06 | 1.04 | 1.28 | . |
| Separated | 1.28 | 0.16 | 0.97 | 1.60 | . |
| Divorced | 1.30 | 0.12 | 1.06 | 1.55 | . |
| Widowed | 0.69 | 0.13 | 0.43 | 0.96 | . |
| Domestic Partner | 1.90 | 0.16 | 1.59 | 2.22 | . |
| Employment |  |  |  |  |  |
| Employed for an Employer | 1.34 | 0.07 | 1.20 | 1.47 | 0.069 |
| Self-Employed | 1.37 | 0.11 | 1.15 | 1.59 | . |
| Retired | 1.02 | 0.13 | 0.77 | 1.27 | . |
| Student | 1.42 | 0.12 | 1.19 | 1.65 | . |
| Homemaker | 1.13 | 0.12 | 0.88 | 1.37 | . |
| Unemployed and Looking for a Job | 1.25 | 0.09 | 1.07 | 1.43 | . |
| None of These/Other | 1.46 | 0.12 | 1.22 | 1.69 | . |
| Education |  |  |  |  |  |
| Up to 8 Years | 0.53 | 0.10 | 0.34 | 0.72 | 0.00 |
| 9-15 Years | 1.19 | 0.07 | 1.04 | 1.33 | . |
| 16+ Years | 1.46 | 0.06 | 1.34 | 1.58 | . |
| Service Attendance |  |  |  |  |  |
| >1/Week | 0.85 | 0.08 | 0.70 | 1.00 | 0.00 |
| 1/Week | 1.15 | 0.09 | 0.97 | 1.32 | . |
| 1-3/Month | 1.14 | 0.09 | 0.97 | 1.31 | . |
| A Few Times a Year | 1.47 | 0.09 | 1.29 | 1.64 | . |
| Never | 1.49 | 0.08 | 1.33 | 1.65 | . |
| Immigration Status |  |  |  |  |  |
| Born in This Country | 1.32 | 0.06 | 1.21 | 1.43 | 0.126 |
| Born in Another Country | 1.18 | 0.09 | 1.00 | 1.36 | . |
| Religion |  |  |  |  |  |
| Christianity | 1.41 | 0.29 | 0.82 | 2.00 | 0.00 |
| Islam | 1.38 | 0.10 | 1.19 | 1.57 | . |
| Judaism | 1.26 | 0.06 | 1.14 | 1.38 | . |
| Baha'i | 0.00 | . | . | . | . |
| Taoism | 1.00 | . | . | . | . |
| Primal, Animist, or Folk Religion | 0.00 | . | . | . | . |
| Some Other Religion | 0.97 | . | . | . | . |
| No Religion/Atheist/Agnostic | 1.55 | 0.33 | 0.89 | 2.22 | . |
| Race/Ethnicity |  |  |  |  |  |
| Jewish | 1.26 | 0.06 | 1.14 | 1.39 | 0.556 |
| Arab | 1.38 | 0.10 | 1.19 | 1.57 | . |
| Other | 1.36 | 0.20 | 0.96 | 1.77 | . |

**Table 10a: Nationally Representative Descriptive Statistics of the Observed Sample (Japan)**

| Variable | Proportion | Frequency |
| --- | --- | --- |
| Age |  |  |
| 18-24 | 0.08 | 1589 |
| 25-29 | 0.04 | 806 |
| 30-39 | 0.14 | 2851 |
| 40-49 | 0.16 | 3363 |
| 50-59 | 0.18 | 3770 |
| 60-69 | 0.20 | 4118 |
| 70-79 | 0.17 | 3554 |
| 80 or Older | 0.02 | 493 |
| Missing | . | . |
| Gender |  |  |
| Male | 0.48 | 9847 |
| Female | 0.52 | 10602 |
| Other | 0.00 | 28 |
| Missing | 0.00 | 66 |
| Marital Status |  |  |
| Single/Never Been Married | 0.24 | 5004 |
| Married | 0.58 | 11837 |
| Separated | 0.01 | 190 |
| Divorced | 0.10 | 2126 |
| Widowed | 0.06 | 1179 |
| Domestic Partner | 0.01 | 144 |
| Missing | 0.00 | 64 |
| Employment |  |  |
| Employed for an Employer | 0.53 | 10853 |
| Self-Employed | 0.09 | 1748 |
| Retired | 0.12 | 2535 |
| Student | 0.02 | 491 |
| Homemaker | 0.06 | 1276 |
| Unemployed and Looking for a Job | 0.03 | 622 |
| None of These/Other | 0.15 | 2983 |
| Missing | 0.00 | 36 |
| Education |  |  |
| Up to 8 Years | 0.03 | 567 |
| 9-15 Years | 0.72 | 14893 |
| 16+ Years | 0.25 | 5083 |
| Missing | . | . |
| Service Attendance |  |  |
| >1/Week | 0.02 | 316 |
| 1/Week | 0.02 | 348 |
| 1-3/Month | 0.04 | 862 |
| A Few Times a Year | 0.15 | 3112 |
| Never | 0.77 | 15788 |
| Missing | 0.01 | 117 |
| Immigration Status |  |  |
| Born in This Country | 0.95 | 19548 |
| Born in Another Country | 0.01 | 158 |
| Missing | 0.04 | 837 |
| Religion |  |  |
| Christianity | 0.02 | 381 |
| Islam | 0.00 | 10 |
| Hinduism | 0.00 | 5 |
| Buddhism | 0.33 | 6709 |
| Judaism | 0.00 | 10 |
| Sikhism | 0.00 | 6 |
| Baha'i | 0.00 | 2 |
| Jainism | 0.00 | 11 |
| Shinto | 0.02 | 469 |
| Taoism | 0.00 | 7 |
| Confucianism | 0.00 | 17 |
| Primal, Animist, or Folk Religion | 0.00 | 19 |
| Spiritism | . | . |
| African-Derived | . | . |
| Chinese | . | . |
| Some Other Religion | 0.00 | 46 |
| No Religion/Atheist/Agnostic | 0.61 | 12497 |
| Missing | 0.02 | 355 |
| Race/Ethnicity |  |  |
| No Data | . | . |

**Table 10b: Variations Across Demographic Characteristics (Japan)**

| Variable | Mean | SE | LCI | UCI | Global p-value |
| --- | --- | --- | --- | --- | --- |
| Age |  |  |  |  |  |
| 18-24 | 1.81 | 0.07 | 1.68 | 1.95 | 0.00 |
| 25-29 | 1.55 | 0.08 | 1.39 | 1.71 | . |
| 30-39 | 1.67 | 0.05 | 1.57 | 1.77 | . |
| 40-49 | 1.59 | 0.05 | 1.50 | 1.69 | . |
| 50-59 | 1.63 | 0.04 | 1.55 | 1.72 | . |
| 60-69 | 2.26 | 0.04 | 2.17 | 2.35 | . |
| 70-79 | 3.02 | 0.05 | 2.92 | 3.12 | . |
| 80 or Older | 3.12 | 0.14 | 2.84 | 3.40 | . |
| Gender |  |  |  |  |  |
| Male | 2.18 | 0.03 | 2.12 | 2.24 | 0.00 |
| Female | 1.91 | 0.03 | 1.86 | 1.97 | . |
| Other | 1.89 | 0.38 | 1.11 | 2.67 | . |
| Marital Status |  |  |  |  |  |
| Single/Never Been Married | 1.76 | 0.03 | 1.70 | 1.83 | 0.00 |
| Married | 2.07 | 0.03 | 2.01 | 2.12 | . |
| Separated | 2.32 | 0.23 | 1.87 | 2.76 | . |
| Divorced | 2.08 | 0.07 | 1.94 | 2.23 | . |
| Widowed | 2.91 | 0.10 | 2.71 | 3.11 | . |
| Domestic Partner | 1.70 | 0.25 | 1.21 | 2.20 | . |
| Employment |  |  |  |  |  |
| Employed for an Employer | 1.76 | 0.03 | 1.71 | 1.82 | 0.00 |
| Self-Employed | 2.27 | 0.07 | 2.12 | 2.42 | . |
| Retired | 2.87 | 0.06 | 2.76 | 2.99 | . |
| Student | 1.78 | 0.09 | 1.60 | 1.95 | . |
| Homemaker | 2.23 | 0.07 | 2.09 | 2.37 | . |
| Unemployed and Looking for a Job | 1.58 | 0.09 | 1.41 | 1.76 | . |
| None of These/Other | 2.28 | 0.05 | 2.19 | 2.38 | . |
| Education |  |  |  |  |  |
| Up to 8 Years | 2.01 | 0.13 | 1.75 | 2.26 | 0.06 |
| 9-15 Years | 1.99 | 0.02 | 1.94 | 2.04 | . |
| 16+ Years | 2.20 | 0.04 | 2.12 | 2.28 | . |
| Service Attendance |  |  |  |  |  |
| >1/Week | 3.14 | 0.17 | 2.81 | 3.46 | 0.00 |
| 1/Week | 2.55 | 0.15 | 2.26 | 2.84 | . |
| 1-3/Month | 2.45 | 0.09 | 2.26 | 2.63 | . |
| A Few Times a Year | 2.27 | 0.05 | 2.17 | 2.37 | . |
| Never | 1.94 | 0.02 | 1.90 | 1.99 | . |
| Immigration Status |  |  |  |  |  |
| Born in This Country | 2.04 | 0.02 | 2.00 | 2.08 | 0.065 |
| Born in Another Country | 2.46 | 0.23 | 2.01 | 2.91 | . |
| Religion |  |  |  |  |  |
| Christianity | 2.61 | 0.15 | 2.32 | 2.89 | 0.00 |
| Islam | 2.68 | 0.73 | 0.91 | 4.45 | . |
| Hinduism | 2.21 | 0.87 | 0.00 | 4.42 | . |
| Buddhism | 2.30 | 0.04 | 2.23 | 2.37 | . |
| Judaism | 2.34 | 0.49 | 0.98 | 3.70 | . |
| Sikhism | 2.15 | 0.46 | 1.16 | 3.14 | . |
| Baha'i | 4.50 | 1.45 | -0.47 | 9.48 | . |
| Jainism | 1.90 | 0.57 | 0.43 | 3.37 | . |
| Shinto | 2.37 | 0.14 | 2.10 | 2.65 | . |
| Taoism | 3.34 | 0.71 | 1.71 | 4.97 | . |
| Confucianism | 3.35 | 0.64 | 2.02 | 4.69 | . |
| Primal, Animist, or Folk Religion | 1.86 | 0.57 | 0.64 | 3.09 | . |
| Some Other Religion | 2.66 | 0.56 | 1.52 | 3.80 | . |
| No Religion/Atheist/Agnostic | 1.87 | 0.02 | 1.82 | 1.92 | . |
| Race/Ethnicity |  |  |  |  |  |
| No Data | . | . | . |  | . |

**Table 11a: Nationally Representative Descriptive Statistics of the Observed Sample (Kenya)**

| Variable | Proportion | Frequency |
| --- | --- | --- |
| Age |  |  |
| 18-24 | 0.25 | 2868 |
| 25-29 | 0.18 | 2035 |
| 30-39 | 0.23 | 2564 |
| 40-49 | 0.15 | 1708 |
| 50-59 | 0.09 | 1072 |
| 60-69 | 0.06 | 710 |
| 70-79 | 0.03 | 360 |
| 80 or Older | 0.01 | 67 |
| Missing | 0.00 | 5 |
| Gender |  |  |
| Male | 0.49 | 5567 |
| Female | 0.51 | 5813 |
| Other | 0.00 | 2 |
| Missing | 0.00 | 7 |
| Marital Status |  |  |
| Single/Never Been Married | 0.31 | 3531 |
| Married | 0.58 | 6626 |
| Separated | 0.04 | 467 |
| Divorced | 0.01 | 111 |
| Widowed | 0.04 | 464 |
| Domestic Partner | 0.01 | 146 |
| Missing | 0.00 | 43 |
| Employment |  |  |
| Employed for an Employer | 0.13 | 1467 |
| Self-Employed | 0.32 | 3630 |
| Retired | 0.03 | 319 |
| Student | 0.10 | 1136 |
| Homemaker | 0.13 | 1537 |
| Unemployed and Looking for a Job | 0.28 | 3153 |
| None of These/Other | 0.01 | 138 |
| Missing | 0.00 | 9 |
| Education |  |  |
| Up to 8 Years | 0.39 | 4485 |
| 9-15 Years | 0.54 | 6115 |
| 16+ Years | 0.07 | 783 |
| Missing | 0.00 | 6 |
| Service Attendance |  |  |
| >1/Week | 0.24 | 2774 |
| 1/Week | 0.53 | 6063 |
| 1-3/Month | 0.11 | 1219 |
| A Few Times a Year | 0.08 | 855 |
| Never | 0.04 | 465 |
| Missing | 0.00 | 13 |
| Immigration Status |  |  |
| Born in This Country | 0.99 | 11270 |
| Born in Another Country | 0.01 | 117 |
| Missing | 0.00 | 2 |
| Religion |  |  |
| Christianity | 0.91 | 10334 |
| Islam | 0.08 | 918 |
| Hinduism | . | . |
| Buddhism | 0.00 | 1 |
| Judaism | 0.00 | 3 |
| Sikhism | . | . |
| Baha'i | 0.00 | 1 |
| Jainism | 0.00 | 1 |
| Shinto | . | . |
| Taoism | . | . |
| Confucianism | 0.00 | 3 |
| Primal, Animist, or Folk Religion | 0.00 | 7 |
| Spiritism | . | . |
| African-Derived | . | . |
| Chinese | . | . |
| Some Other Religion | 0.00 | 5 |
| No Religion/Atheist/Agnostic | 0.01 | 108 |
| Missing | 0.00 | 9 |
| Race/Ethnicity |  |  |
| Luhya | 0.17 | 1943 |
| Luo | 0.10 | 1120 |
| Kalenjin | 0.12 | 1377 |
| Kamba | 0.11 | 1299 |
| Kikuyu | 0.19 | 2118 |
| Kisii | 0.07 | 789 |
| Maasai | 0.02 | 237 |
| Meru | 0.06 | 630 |
| Kenan Somali/Somali | 0.03 | 396 |
| Miji Kenda Tribes | 0.06 | 708 |
| Embu | 0.02 | 197 |
| Other | 0.05 | 548 |
| Missing | 0.00 | 27 |

**Table 11b: Variations Across Demographic Characteristics (Kenya)**

| Variable | Mean | SE | LCI | UCI | Global p-value |
| --- | --- | --- | --- | --- | --- |
| Age |  |  |  |  |  |
| 18-24 | 2.52 | 0.06 | 2.41 | 2.63 | 0.00 |
| 25-29 | 2.43 | 0.07 | 2.30 | 2.56 | . |
| 30-39 | 2.52 | 0.07 | 2.39 | 2.66 | . |
| 40-49 | 2.80 | 0.10 | 2.60 | 3.00 | . |
| 50-59 | 2.87 | 0.13 | 2.60 | 3.13 | . |
| 60-69 | 3.25 | 0.19 | 2.88 | 3.62 | . |
| 70-79 | 2.90 | 0.28 | 2.34 | 3.46 | . |
| 80 or Older | 2.86 | 0.41 | 2.02 | 3.70 | . |
| Gender |  |  |  |  |  |
| Male | 3.22 | 0.05 | 3.12 | 3.33 | 0.00 |
| Female | 2.08 | 0.07 | 1.95 | 2.21 | . |
| Other | 1.60 | . | . | . | . |
| Marital Status |  |  |  |  |  |
| Single/Never Been Married | 2.65 | 0.06 | 2.54 | 2.76 | 0.967 |
| Married | 2.63 | 0.07 | 2.50 | 2.75 | . |
| Separated | 2.59 | 0.15 | 2.28 | 2.89 | . |
| Divorced | 2.74 | 0.27 | 2.21 | 3.27 | . |
| Widowed | 2.77 | 0.21 | 2.37 | 3.18 | . |
| Domestic Partner | 2.67 | 0.27 | 2.14 | 3.20 | . |
| Employment |  |  |  |  |  |
| Employed for an Employer | 2.82 | 0.10 | 2.62 | 3.01 | 0.00 |
| Self-Employed | 2.76 | 0.07 | 2.62 | 2.89 | . |
| Retired | 3.36 | 0.26 | 2.85 | 3.86 | . |
| Student | 2.68 | 0.08 | 2.52 | 2.84 | . |
| Homemaker | 2.31 | 0.13 | 2.06 | ,2.56 | . |
| Unemployed and Looking for a Job | 2.47 | 0.06 | 2.34 | 2.59 | . |
| None of These/Other | 3.23 | 0.33 | 2.57 | 3.90 | . |
| Education |  |  |  |  |  |
| Up to 8 Years | 2.54 | 0.08 | 2.38 | 2.69 | 0.055 |
| 9-15 Years | 2.69 | 0.05 | 2.59 | 2.79 | . |
| 16+ Years | 2.83 | 0.11 | 2.61 | 3.05 | . |
| Service Attendance |  |  |  |  |  |
| >1/Week | 2.75 | 0.08 | 2.60 | 2.90 | 0.152 |
| 1/Week | 2.58 | 0.06 | 2.47 | 2.69 | . |
| 1-3/Month | 2.63 | 0.10 | 2.44 | 2.82 | . |
| A Few Times a Year | 2.59 | 0.13 | 2.34 | 2.85 | . |
| Never | 2.85 | 0.16 | 2.53 | 3.17 | . |
| Immigration Status |  |  |  |  |  |
| Born in This Country | 2.64 | 0.05 | 2.54 | 2.74 | 0.671 |
| Born in Another Country | 2.49 | 0.35 | 1.79 | 3.19 | . |
| Religion |  |  |  |  |  |
| Christianity | 2.61 | 0.05 | 2.50 | 2.71 | 0.00 |
| Islam | 3.02 | 0.16 | 2.71 | 3.33 | . |
| Buddhism | 3.00 | . | . | . | . |
| Judaism | 0.17 | . | . | . | . |
| Baha'i | 7.00 | . | . | . | . |
| Jainism | 2.00 | . | . | . | . |
| Confucianism | 0.00 | . | . | . | . |
| Primal, Animist, or Folk Religion | 2.08 | . | . | . | . |
| Some Other Religion | 2.75 | 0.98 | 0.13 | 5.38 | . |
| No Religion/Atheist/Agnostic | 2.84 | 0.33 | 2.18 | 3.49 | . |
| Race/Ethnicity |  |  |  |  |  |
| Luhya | 2.35 | 0.06 | 2.23 | 2.48 | 0.00 |
| Luo | 2.61 | 0.10 | 2.42 | 2.80 | . |
| Kalenjin | 2.82 | 0.11 | 2.59 | 3.04 | . |
| Kamba | 2.42 | 0.13 | 2.17 | 2.67 | . |
| Kikuyu | 2.86 | 0.13 | 2.60 | 3.13 | . |
| Kisii | 2.21 | 0.24 | 1.74 | 2.69 | . |
| Maasai | 3.32 | 0.24 | 2.84 | 3.79 | . |
| Meru | 2.93 | 0.16 | 2.61 | 3.24 | . |
| Kenan Somali/Somali | 3.26 | 0.27 | 2.73 | 3.79 | . |
| Miji Kenda Tribes | 2.72 | 0.24 | 2.26 | 3.18 | . |
| Embu | 2.14 | 0.13 | 1.88 | 2.40 | . |
| Other | 2.54 | 0.16 | 2.21 | 2.86 | . |

**Table 12a: Nationally Representative Descriptive Statistics of the Observed Sample (Mexico)**

| Variable | Proportion | Frequency |
| --- | --- | --- |
| Age |  |  |
| 18-24 | 0.17 | 986 |
| 25-29 | 0.11 | 623 |
| 30-39 | 0.23 | 1312 |
| 40-49 | 0.18 | 1027 |
| 50-59 | 0.15 | 873 |
| 60-69 | 0.11 | 611 |
| 70-79 | 0.05 | 277 |
| 80 or Older | 0.01 | 68 |
| Missing | . | . |
| Gender |  |  |
| Male | 0.48 | 2755 |
| Female | 0.52 | 2997 |
| Other | 0.00 | 3 |
| Missing | 0.00 | 21 |
| Marital Status |  |  |
| Single/Never Been Married | 0.25 | 1432 |
| Married | 0.36 | 2089 |
| Separated | 0.07 | 403 |
| Divorced | 0.04 | 230 |
| Widowed | 0.06 | 347 |
| Domestic Partner | 0.19 | 1109 |
| Missing | 0.03 | 166 |
| Employment |  |  |
| Employed for an Employer | 0.33 | 1921 |
| Self-Employed | 0.19 | 1091 |
| Retired | 0.07 | 386 |
| Student | 0.04 | 247 |
| Homemaker | 0.22 | 1257 |
| Unemployed and Looking for a Job | 0.10 | 564 |
| None of These/Other | 0.03 | 169 |
| Missing | 0.02 | 141 |
| Education |  |  |
| Up to 8 Years | 0.22 | 1291 |
| 9-15 Years | 0.55 | 3180 |
| 16+ Years | 0.23 | 1304 |
| Missing | 0.00 | 1 |
| Service Attendance |  |  |
| >1/Week | 0.11 | 609 |
| 1/Week | 0.22 | 1260 |
| 1-3/Month | 0.12 | 676 |
| A Few Times a Year | 0.36 | 2054 |
| Never | 0.20 | 1134 |
| Missing | 0.01 | 43 |
| Immigration Status |  |  |
| Born in This Country | 0.96 | 5517 |
| Born in Another Country | 0.02 | 108 |
| Missing | 0.03 | 151 |
| Religion |  |  |
| Christianity | 0.84 | 4844 |
| Islam | 0.00 | 2 |
| Hinduism | 0.00 | 3 |
| Buddhism | 0.00 | 6 |
| Judaism | 0.00 | 7 |
| Sikhism | . | . |
| Baha'i | 0.00 | 1 |
| Jainism | 0.00 | 1 |
| Shinto | 0.00 | 2 |
| Taoism | 0.00 | 4 |
| Confucianism | 0.00 | 1 |
| Primal, Animist, or Folk Religion | 0.00 | 20 |
| Spiritism | . | . |
| African-Derived | . | . |
| Chinese | . | . |
| Some Other Religion | 0.01 | 41 |
| No Religion/Atheist/Agnostic | 0.13 | 770 |
| Missing | 0.01 | 75 |
| Race/Ethnicity |  |  |
| White | 0.19 | 1116 |
| Mestizo | 0.48 | 2762 |
| Indigenous | 0.10 | 594 |
| Black | 0.02 | 108 |
| Mulatto | 0.01 | 63 |
| Other | 0.06 | 339 |
| Missing | 0.14 | 794 |

**Table 12b: Variations Across Demographic Characteristics (Mexico)**

| Variable | Mean | SE | LCI | UCI | Global p-value |
| --- | --- | --- | --- | --- | --- |
| Age |  |  |  |  |  |
| 18-24 | 2.61 | 0.10 | 2.41 | 2.81 | 0.298 |
| 25-29 | 2.33 | 0.11 | 2.12 | 2.54 | . |
| 30-39 | 2.35 | 0.09 | 2.18 | 2.52 | . |
| 40-49 | 2.35 | 0.10 | 2.15 | 2.55 | . |
| 50-59 | 2.46 | 0.12 | 2.22 | 2.70 | . |
| 60-69 | 2.54 | 0.17 | 2.21 | 2.87 | . |
| 70-79 | 2.85 | 0.28 | 2.29 | 3.41 | . |
| 80 or Older | 2.16 | 0.39 | 1.37 | 2.96 | . |
| Gender |  |  |  |  |  |
| Male | 2.77 | 0.07 | 2.64 | 2.90 | 0.00 |
| Female | 2.16 | 0.06 | 2.04 | 2.27 | . |
| Other | 0.74 | 0.16 | 0.35 | 1.13 | . |
| Marital Status |  |  |  |  |  |
| Single/Never Been Married | 2.58 | 0.09 | 2.40 | 2.75 | 0.119 |
| Married | 2.46 | 0.07 | 2.32 | 2.60 | . |
| Separated | 2.35 | 0.16 | 2.03 | 2.68 | . |
| Divorced | 2.50 | 0.21 | 2.08 | 2.92 | . |
| Widowed | 2.66 | 0.23 | 2.21 | 3.11 | . |
| Domestic Partner | 2.23 | 0.10 | 2.05 | 2.42 | . |
| Employment |  |  |  |  |  |
| Employed for an Employer | 2.65 | 0.07 | 2.51 | 2.80 | 0.00 |
| Self-Employed | 2.62 | 0.10 | 2.42 | 2.82 | . |
| Retired | 2.56 | 0.20 | 2.17 | 2.95 | . |
| Student | 2.84 | 0.20 | 2.44 | 3.24 | . |
| Homemaker | 1.95 | 0.10 | 1.76 | 2.13 | . |
| Unemployed and Looking for a Job | 2.26 | 0.13 | 2.00 | 2.51 | . |
| None of These/Other | 2.63 | 0.27 | 2.09 | 3.16 | . |
| Education |  |  |  |  |  |
| Up to 8 Years | 2.41 | 0.11 | 2.20 | 2.62 | 0.00 |
| 9-15 Years | 2.34 | 0.06 | 2.23 | 2.46 | . |
| 16+ Years | 2.75 | 0.09 | 2.58 | 2.92 | . |
| Service Attendance |  |  |  |  |  |
| >1/Week | 2.98 | 0.14 | 2.72 | 3.25 | 0.00 |
| 1/Week | 2.49 | 0.09 | 2.30 | 2.67 | . |
| 1-3/Month | 2.45 | 0.13 | 2.20 | 2.71 | . |
| A Few Times a Year | 2.36 | 0.07 | 2.22 | 2.51 | . |
| Never | 2.28 | 0.10 | 2.08 | 2.48 | . |
| Immigration Status |  |  |  |  |  |
| Born in This Country | 2.46 | 0.05 | 2.37 | 2.55 | 0.101 |
| Born in Another Country | 2.06 | 0.24 | 1.58 | 2.54 | . |
| Religion |  |  |  |  |  |
| Christianity | 2.44 | 0.05 | 2.34 | 2.53 | 0.00 |
| Islam | 0.60 | . | . | . | . |
| Hinduism | 3.37 | 1.12 | -0.47 | 7.21 | . |
| Buddhism | 1.31 | 0.78 | -0.57 | 3.18 | . |
| Judaism | 4.79 | 1.08 | 2.21 | 7.37 | . |
| Baha'i | 2.00 | . | . | . | . |
| Jainism | 2.00 | . | . | . | . |
| Shinto | 3.67 | . | . | . | . |
| Taoism | 4.69 | . | . | . | . |
| Confucianism | 4.00 | . | . | . | . |
| Primal, Animist, or Folk Religion | 4.00 | 1.05 | 1.76 | 6.24 | . |
| Some Other Religion | 2.15 | 0.39 | 1.36 | 2.94 | . |
| No Religion/Atheist/Agnostic | 2.49 | 0.12 | 2.26 | 2.72 | . |
| Race/Ethnicity |  |  |  |  |  |
| White | 2.53 | 0.10 | 2.33 | 2.73 | 0.407 |
| Mestizo | 2.46 | 0.06 | 2.34 | 2.58 | . |
| Indigenous | 2.22 | 0.13 | 1.96 | 2.48 | . |
| Black | 2.46 | 0.34 | 1.77 | 3.15 | . |
| Mulatto | 2.48 | 0.37 | 1.75 | 3.21 | . |
| Other | 2.55 | 0.19 | 2.17 | 2.93 | . |

**Table 13a: Nationally Representative Descriptive Statistics of the Observed Sample (Nigeria)**

| Variable | Proportion | Frequency |
| --- | --- | --- |
| Age |  |  |
| 18-24 | 0.22 | 1533 |
| 25-29 | 0.17 | 1193 |
| 30-39 | 0.28 | 1943 |
| 40-49 | 0.16 | 1059 |
| 50-59 | 0.09 | 619 |
| 60-69 | 0.04 | 296 |
| 70-79 | 0.02 | 133 |
| 80 or Older | 0.01 | 50 |
| Missing | . | . |
| Gender |  |  |
| Male | 0.49 | 3371 |
| Female | 0.51 | 3456 |
| Other | 0.00 | 0 |
| Missing | . | . |
| Marital Status |  |  |
| Single/Never Been Married | 0.34 | 2289 |
| Married | 0.60 | 4065 |
| Separated | 0.02 | 117 |
| Divorced | 0.01 | 71 |
| Widowed | 0.03 | 231 |
| Domestic Partner | 0.00 | 12 |
| Missing | 0.01 | 42 |
| Employment |  |  |
| Employed for an Employer | 0.10 | 699 |
| Self-Employed | 0.57 | 3898 |
| Retired | 0.03 | 178 |
| Student | 0.10 | 650 |
| Homemaker | 0.07 | 499 |
| Unemployed and Looking for a Job | 0.10 | 684 |
| None of These/Other | 0.03 | 211 |
| Missing | 0.00 | 8 |
| Education |  |  |
| Up to 8 Years | 0.38 | 2575 |
| 9-15 Years | 0.60 | 4120 |
| 16+ Years | 0.02 | 130 |
| Missing | 0.00 | 2 |
| Service Attendance |  |  |
| >1/Week | 0.59 | 4049 |
| 1/Week | 0.28 | 1895 |
| 1-3/Month | 0.08 | 531 |
| A Few Times a Year | 0.04 | 254 |
| Never | 0.01 | 77 |
| Missing | 0.00 | 20 |
| Immigration Status |  |  |
| Born in This Country | 0.99 | 6779 |
| Born in Another Country | 0.01 | 47 |
| Missing | 0.00 | 1 |
| Religion |  |  |
| Christianity | 0.51 | 3476 |
| Islam | 0.48 | 3302 |
| Hinduism | . | . |
| Buddhism | . | . |
| Judaism | . | . |
| Sikhism | . | . |
| Baha'i | . | . |
| Jainism | . | . |
| Shinto | 0.00 | 1 |
| Taoism | . | . |
| Confucianism | 0.00 | 0 |
| Primal, Animist, or Folk Religion | 0.00 | 24 |
| Spiritism | . | . |
| African-Derived | . | . |
| Chinese | . | . |
| Some Other Religion | 0.00 | 1 |
| No Religion/Atheist/Agnostic | 0.00 | 15 |
| Missing | 0.00 | 9 |
| Race/Ethnicity |  |  |
| Hausa | 0.34 | 2342 |
| Yoruba | 0.18 | 1230 |
| Igbo (Ibo) | 0.16 | 1112 |
| Edo | 0.02 | 116 |
| Urhobo | 0.01 | 38 |
| Fulani | 0.04 | 266 |
| Kanuri | 0.00 | 31 |
| Tiv | 0.03 | 198 |
| Efik | 0.01 | 48 |
| Ijaw | 0.02 | 110 |
| Igala | 0.01 | 77 |
| Ibibio | 0.03 | 180 |
| Idoma | 0.01 | 61 |
| Other | 0.15 | 1014 |
| Missing | 0.00 | 4 |

**Table 13b: Variations Across Demographic Characteristics (Nigeria)**

| Variable | Mean | SE | LCI | UCI | Global p-value |
| --- | --- | --- | --- | --- | --- |
| Age |  |  |  |  |  |
| 18-24 | 2.97 | 0.09 | 2.78 | 3.15 | 0.009 |
| 25-29 | 2.96 | 0.09 | 2.77 | 3.15 | . |
| 30-39 | 3.02 | 0.09 | 2.85 | 3.19 | . |
| 40-49 | 3.11 | 0.14 | 2.83 | 3.38 | . |
| 50-59 | 3.29 | 0.20 | 2.88 | 3.69 | . |
| 60-69 | 3.03 | 0.32 | 2.38 | 3.67 | . |
| 70-79 | 1.76 | 0.44 | 0.87 | 2.66 | . |
| 80 or Older | 1.36 | 0.36 | 0.55 | 2.17 | . |
| Gender |  |  |  |  |  |
| Male | 3.42 | 0.07 | 3.28 | 3.56 | 0.00 |
| Female | 2.59 | 0.08 | 2.44 | 2.74 | . |
| Other | 3.00 | . | . |  | . |
| Marital Status |  |  |  |  |  |
| Single/Never Been Married | 3.17 | 0.07 | 3.03 | 3.32 | 0.017 |
| Married | 2.94 | 0.08 | 2.78 | 3.09 | . |
| Separated | 2.82 | 0.32 | 2.18 | 3.46 | . |
| Divorced | 2.43 | 0.36 | 1.71 | 3.14 | . |
| Widowed | 2.53 | 0.31 | 1.92 | 3.14 | . |
| Domestic Partner | 4.79 | 0.32 | 4.06 | 5.53 | . |
| Employment |  |  |  |  |  |
| Employed for an Employer | 3.09 | 0.10 | 2.89 | 3.29 | 0.00 |
| Self-Employed | 3.14 | 0.08 | 2.98 | 3.30 | . |
| Retired | 2.19 | 0.34 | 1.52 | 2.87 | . |
| Student | 2.91 | 0.11 | 2.69 | 3.12 | . |
| Homemaker | 2.20 | 0.18 | 1.84 | 2.56 | . |
| Unemployed and Looking for a Job | 3.08 | 0.13 | 2.82 | 3.34 | . |
| None of These/Other | 2.68 | 0.36 | 1.97 | 3.38 | . |
| Education |  |  |  |  |  |
| Up to 8 Years | 2.94 | 0.12 | 2.71 | 3.17 | 0.688 |
| 9-15 Years | 3.04 | 0.05 | 2.93 | 3.14 | . |
| 16+ Years | 3.04 | 0.19 | 2.67 | 3.42 | . |
| Service Attendance |  |  |  |  |  |
| >1/Week | 3.08 | 0.08 | 2.93 | 3.22 | 0.008 |
| 1/Week | 2.86 | 0.10 | 2.67 | 3.05 | . |
| 1-3/Month | 2.69 | 0.16 | 2.38 | 3.01 | . |
| A Few Times a Year | 3.51 | 0.25 | 3.02 | 4.00 | . |
| Never | 2.85 | 0.43 | 1.98 | 3.72 | . |
| Immigration Status |  |  |  |  |  |
| Born in This Country | 3.00 | 0.06 | 2.88 | 3.12 | 0.830 |
| Born in Another Country | 2.99 | 0.52 | 1.94 | 4.03 | . |
| Religion |  |  |  |  |  |
| Christianity | 3.09 | 0.07 | 2.94 | 3.23 | 0.00 |
| Islam | 2.90 | 0.10 | 2.70 | 3.09 | . |
| Shinto | 1.00 | . | . |  | . |
| Confucianism | 3.00 | . | . |  | . |
| Primal, Animist, or Folk Religion | 3.40 | 0.91 | 1.43 | 5.38 | . |
| Some Other Religion | 0.17 | . | . |  | . |
| No Religion/Atheist/Agnostic | 5.25 | 0.31 | 4.48 | 6.03 | . |
| Race/Ethnicity |  |  |  |  |  |
| Hausa | 2.95 | 0.12 | 2.72 | 3.17 | 0.597 |
| Yoruba | 3.04 | 0.12 | 2.80 | 3.28 | . |
| Igbo (Ibo) | 3.11 | 0.11 | 2.89 | 3.33 | . |
| Edo | 3.11 | 0.41 | 2.30 | 3.91 | . |
| Urhobo | 3.39 | 0.58 | 2.23 | 4.56 | . |
| Fulani | 2.47 | 0.27 | 1.93 | 3.01 | . |
| Kanuri | 3.26 | 0.59 | 2.06 | 4.46 | . |
| Tiv | 3.31 | 0.27 | 2.78 | 3.85 | . |
| Efik | 3.27 | 0.49 | 2.29 | 4.26 | . |
| Ijaw | 2.28 | 0.63 | 1.03 | 3.52 | . |
| Igala | 2.91 | 0.45 | 2.01 | 3.81 | . |
| Ibibio | 3.01 | 0.33 | 2.37 | 3.66 | . |
| Idoma | 3.47 | 0.35 | 2.77 | 4.17 | . |
| Other | 3.03 | 0.13 | 2.77 | 3.29 | . |

**Table 14a: Nationally Representative Descriptive Statistics of the Observed Sample (Philippines)**

| Variable | Proportion | Frequency |
| --- | --- | --- |
| Age |  |  |
| 18-24 | 0.20 | 1073 |
| 25-29 | 0.13 | 695 |
| 30-39 | 0.22 | 1160 |
| 40-49 | 0.18 | 972 |
| 50-59 | 0.14 | 732 |
| 60-69 | 0.09 | 495 |
| 70-79 | 0.03 | 143 |
| 80 or Older | 0.00 | 23 |
| Missing | . | . |
| Gender |  |  |
| Male | 0.50 | 2625 |
| Female | 0.50 | 2643 |
| Other | 0.00 | 13 |
| Missing | 0.00 | 11 |
| Marital Status |  |  |
| Single/Never Been Married | 0.23 | 1206 |
| Married | 0.45 | 2385 |
| Separated | 0.05 | 249 |
| Divorced | 0.00 | 9 |
| Widowed | 0.05 | 274 |
| Domestic Partner | 0.22 | 1152 |
| Missing | 0.00 | 16 |
| Employment |  |  |
| Employed for an Employer | 0.26 | 1350 |
| Self-Employed | 0.26 | 1379 |
| Retired | 0.03 | 158 |
| Student | 0.11 | 585 |
| Homemaker | 0.20 | 1049 |
| Unemployed and Looking for a Job | 0.12 | 658 |
| None of These/Other | 0.02 | 113 |
| Missing | . | . |
| Education |  |  |
| Up to 8 Years | 0.22 | 1188 |
| 9-15 Years | 0.70 | 3722 |
| 16+ Years | 0.07 | 381 |
| Missing | 0.00 | 1 |
| Service Attendance |  |  |
| >1/Week | 0.16 | 844 |
| 1/Week | 0.36 | 1929 |
| 1-3/Month | 0.26 | 1374 |
| A Few Times a Year | 0.18 | 929 |
| Never | 0.04 | 210 |
| Missing | 0.00 | 6 |
| Immigration Status |  |  |
| Born in This Country | 1.00 | 5284 |
| Born in Another Country | 0.00 | 8 |
| Missing | . | . |
| Religion |  |  |
| Christianity | 0.93 | 4914 |
| Islam | 0.06 | 297 |
| Hinduism | . | . |
| Buddhism | 0.00 | 4 |
| Judaism | 0.00 | 4 |
| Sikhism | . | . |
| Baha'i | 0.00 | 1 |
| Jainism | . | . |
| Shinto | . | . |
| Taoism | . | . |
| Confucianism | . | . |
| Primal, Animist, or Folk Religion | 0.00 | 5 |
| Spiritism | . | . |
| African-Derived | . | . |
| Chinese | . | . |
| Some Other Religion | 0.01 | 35 |
| No Religion/Atheist/Agnostic | 0.00 | 23 |
| Missing | 0.00 | 9 |
| Race/Ethnicity |  |  |
| Tagalog | 0.32 | 1691 |
| Cebuana | 0.12 | 656 |
| Ilocano/Ilokano | 0.08 | 429 |
| Visayan/Bisaya | 0.14 | 739 |
| Ilonggo/Hiligaynon | 0.08 | 428 |
| Bicolano/Bikolano | 0.06 | 300 |
| Waray | 0.04 | 216 |
| Tausug | 0.02 | 94 |
| Maranao | 0.01 | 39 |
| Maguindanaoan | 0.02 | 84 |
| Chinese-Filipino | 0.00 | 3 |
| Kapampangan | 0.02 | 107 |
| Pangasinese | 0.02 | 107 |
| Zamboangueno | 0.01 | 51 |
| Malay | . | . |
| Masbateno | 0.01 | 54 |
| Aeta | 0.00 | 1 |
| Igorot | 0.01 | 42 |
| Mangyan | 0.00 | 2 |
| Badjao | 0.00 | 2 |
| Other | 0.05 | 244 |
| Missing | 0.00 | 3 |

**Table 14b: Variations Across Demographic Characteristics (Philippines)**

| Variable | Mean | SE | LCI | UCI | Global p-value |
| --- | --- | --- | --- | --- | --- |
| Age |  |  |  |  |  |
| 18-24 | 2.98 | 0.11 | 2.78 | 3.19 | 0.00 |
| 25-29 | 3.57 | 0.14 | 3.29 | 3.86 | . |
| 30-39 | 3.80 | 0.09 | 3.62 | 3.99 | . |
| 40-49 | 3.99 | 0.10 | 3.79 | 4.18 | . |
| 50-59 | 4.42 | 0.12 | 4.18 | 4.67 | . |
| 60-69 | 4.62 | 0.17 | 4.29 | 4.95 | . |
| 70-79 | 4.68 | 0.27 | 4.14 | 5.22 | . |
| 80 or Older | 3.21 | 0.31 | 2.57 | 3.86 | . |
| Gender |  |  |  |  |  |
| Male | 4.07 | 0.08 | 3.92 | 4.22 | 0.00 |
| Female | 3.59 | 0.05 | 3.48 | 3.70 | . |
| Other | 1.76 | 0.61 | 0.47 | 3.06 | . |
| Marital Status |  |  |  |  |  |
| Single/Never Been Married | 3.06 | 0.11 | 2.85 | 3.27 | 0.00 |
| Married | 4.05 | 0.07 | 3.92 | 4.19 | . |
| Separated | 4.09 | 0.23 | 3.63 | 4.55 | . |
| Divorced | 4.49 | 0.28 | 3.85 | 5.13 | . |
| Widowed | 4.45 | 0.19 | 4.08 | 4.82 | . |
| Domestic Partner | 3.94 | 0.09 | 3.76 | 4.13 | . |
| Employment |  |  |  |  |  |
| Employed for an Employer | 3.87 | 0.10 | 3.68 | 4.06 | 0.00 |
| Self-Employed | 4.23 | 0.10 | 4.03 | 4.44 | . |
| Retired | 4.51 | 0.27 | 3.97 | 5.05 | . |
| Student | 2.76 | 0.14 | 2.49 | 3.04 | . |
| Homemaker | 3.83 | 0.09 | 3.65 | 4.01 | . |
| Unemployed and Looking for a Job | 3.66 | 0.15 | 3.37 | 3.94 | . |
| None of These/Other | 3.67 | 0.30 | 3.08 | 4.27 | . |
| Education |  |  |  |  |  |
| Up to 8 Years | 4.38 | 0.11 | 4.17 | 4.60 | 0.00 |
| 9-15 Years | 3.71 | 0.05 | 3.61 | 3.81 | . |
| 16+ Years | 3.20 | 0.15 | 2.90 | 3.50 | . |
| Service Attendance |  |  |  |  |  |
| >1/Week | 3.76 | 0.12 | 3.52 | 4.00 | 0.00 |
| 1/Week | 4.07 | 0.08 | 3.91 | 4.23 | . |
| 1-3/Month | 3.83 | 0.09 | 3.64 | 4.01 | . |
| A Few Times a Year | 3.42 | 0.11 | 3.20 | 3.65 | . |
| Never | 3.58 | 0.23 | 3.14 | 4.03 | . |
| Immigration Status |  |  |  |  |  |
| Born in This Country | 3.83 | 0.05 | 3.73 | 3.92 | 0.003 |
| Born in Another Country | 2.27 | 0.42 | 1.20 | 3.33 | . |
| Religion |  |  |  |  |  |
| Christianity | 3.81 | 0.05 | 3.71 | 3.91 | 0.00 |
| Islam | 3.89 | 0.19 | 3.51 | 4.27 | . |
| Buddhism | 5.20 | 0.65 | 2.98 | 7.42 | . |
| Judaism | 5.96 | . | . | . | . |
| Baha'i | 3.00 | . | . | . | . |
| Primal, Animist, or Folk Religion | 1.76 | 1.24 | -1.86 | 5.38 | . |
| Some Other Religion | 4.20 | 0.46 | 3.27 | 5.14 | . |
| No Religion/Atheist/Agnostic | 5.12 | 0.29 | 4.52 | 5.73 | . |
| Race/Ethnicity |  |  |  |  |  |
| Tagalog | 3.58 | 0.08 | 3.42 | 3.74 | 0.00 |
| Cebuana | 3.73 | 0.15 | 3.44 | 4.02 | . |
| Ilocano/Ilokano | 4.23 | 0.19 | 3.85 | 4.60 | . |
| Visayan/Bisaya | 3.61 | 0.14 | 3.35 | 3.88 | . |
| Ilonggo/Hiligaynon | 4.35 | 0.11 | 4.13 | 4.58 | . |
| Bicolano/Bikolano | 3.94 | 0.21 | 3.54 | 4.35 | . |
| Waray | 3.73 | 0.29 | 3.15 | 4.31 | . |
| Tausug | 3.81 | 0.35 | 3.12 | 4.50 | . |
| Maranao | 4.50 | 0.25 | 4.00 | 5.00 | . |
| Maguindanaoan | 3.65 | 0.25 | 3.15 | 4.14 | . |
| Chinese-Filipino | 5.67 | 0.60 | 3.93 | 7.41 | . |
| Kapampangan | 4.09 | 0.39 | 3.31 | 4.86 | . |
| Pangasinese | 4.36 | 0.28 | 3.81 | 4.91 | . |
| Zamboangueno | 4.52 | 0.26 | 4.00 | 5.04 | . |
| Malay | 3.81 | 0.65 | 2.50 | 5.11 | . |
| Masbateno | 5.14 | . | . | . | . |
| Aeta | 4.05 | 0.36 | 3.33 | 4.78 | . |
| Igorot | 4.95 | . | . | . | . |
| Mangyan | 0.64 | 0.46 | -0.92 | 2.20 | . |
| Badjao | 4.11 | 0.24 | 3.63 | 4.59 | . |
| Other | 3.58 | 0.08 | 3.42 | 3.74 | . |

**Table 15a: Nationally Representative Descriptive Statistics of the Observed Sample (Poland)**

| Variable | Proportion | Frequency |
| --- | --- | --- |
| Age |  |  |
| 18-24 | 0.09 | 955 |
| 25-29 | 0.07 | 761 |
| 30-39 | 0.21 | 2159 |
| 40-49 | 0.19 | 1956 |
| 50-59 | 0.16 | 1670 |
| 60-69 | 0.18 | 1909 |
| 70-79 | 0.08 | 833 |
| 80 or Older | 0.01 | 145 |
| Missing | 0.00 | 1 |
| Gender |  |  |
| Male | 0.48 | 4974 |
| Female | 0.52 | 5387 |
| Other | 0.00 | 3 |
| Missing | 0.00 | 26 |
| Marital Status |  |  |
| Single/Never Been Married | 0.17 | 1811 |
| Married | 0.58 | 6065 |
| Separated | 0.01 | 111 |
| Divorced | 0.05 | 529 |
| Widowed | 0.10 | 990 |
| Domestic Partner | 0.05 | 504 |
| Missing | 0.04 | 379 |
| Employment |  |  |
| Employed for an Employer | 0.56 | 5837 |
| Self-Employed | 0.07 | 686 |
| Retired | 0.23 | 2434 |
| Student | 0.05 | 515 |
| Homemaker | 0.03 | 338 |
| Unemployed and Looking for a Job | 0.03 | 284 |
| None of These/Other | 0.02 | 169 |
| Missing | 0.01 | 126 |
| Education |  |  |
| Up to 8 Years | 0.12 | 1238 |
| 9-15 Years | 0.59 | 6130 |
| 16+ Years | 0.29 | 3020 |
| Missing | 0.00 | 1 |
| Service Attendance |  |  |
| >1/Week | 0.03 | 305 |
| 1/Week | 0.31 | 3263 |
| 1-3/Month | 0.20 | 2081 |
| A Few Times a Year | 0.29 | 3064 |
| Never | 0.15 | 1597 |
| Missing | 0.01 | 78 |
| Immigration Status |  |  |
| Born in This Country | 0.99 | 10258 |
| Born in Another Country | 0.01 | 108 |
| Missing | 0.00 | 23 |
| Religion |  |  |
| Christianity | 0.90 | 9378 |
| Islam | 0.00 | 2 |
| Hinduism | . | . |
| Buddhism | 0.00 | 2 |
| Judaism | . | . |
| Sikhism | 0.00 | 1 |
| Baha'i | . | . |
| Jainism | 0.00 | 3 |
| Shinto | 0.00 | 1 |
| Taoism | . | . |
| Confucianism | . | . |
| Primal, Animist, or Folk Religion | 0.00 | 11 |
| Spiritism | . | . |
| African-Derived | . | . |
| Chinese | . | . |
| Some Other Religion | . | . |
| No Religion/Atheist/Agnostic | 0.09 | 942 |
| Missing | 0.00 | 50 |
| Race/Ethnicity |  |  |
| Polish | 0.99 | 10309 |
| German | 0.00 | 4 |
| Belarussian | 0.00 | 2 |
| Ukranian | 0.00 | 38 |
| Roma | . | . |
| Russian | . | . |
| Ethnic Jewish | . | . |
| Lemko | . | . |
| Silesia | 0.00 | 14 |
| Kashubians | 0.00 | 3 |
| Other | 0.00 | 4 |
| Missing | 0.00 | 14 |

**Table 15b: Variations Across Demographic Characteristics (Poland)**

| Variable | Mean | SE | LCI | UCI | Global p-value |
| --- | --- | --- | --- | --- | --- |
| Age |  |  |  |  |  |
| 18-24 | 2.41 | 0.13 | 2.15 | 2.66 | 0.00 |
| 25-29 | 1.94 | 0.08 | 1.78 | 2.10 | . |
| 30-39 | 1.55 | 0.06 | 1.44 | 1.67 | . |
| 40-49 | 1.34 | 0.07 | 1.20 | 1.48 | . |
| 50-59 | 1.11 | 0.07 | 0.96 | 1.25 | . |
| 60-69 | 0.89 | 0.07 | 0.75 | 1.03 | . |
| 70-79 | 1.21 | 0.13 | 0.96 | 1.45 | . |
| 80 or Older | 1.04 | 0.22 | 0.60 | 1.49 | . |
| Gender |  |  |  |  |  |
| Male | 1.50 | 0.06 | 1.38 | 1.62 | 0.00 |
| Female | 1.29 | 0.05 | 1.20 | 1.38 | . |
| Other | 1.18 | . | . |  | . |
| Marital Status |  |  |  |  |  |
| Single/Never Been Married | 2.04 | 0.09 | 1.87 | 2.21 | 0.00 |
| Married | 1.24 | 0.05 | 1.14 | 1.35 | . |
| Separated | 1.34 | 0.24 | 0.87 | 1.82 | . |
| Divorced | 1.34 | 0.12 | 1.10 | 1.57 | . |
| Widowed | 0.83 | 0.09 | 0.65 | 1.01 | . |
| Domestic Partner | 2.11 | 0.09 | 1.93 | 2.29 | . |
| Employment |  |  |  |  |  |
| Employed for an Employer | 1.44 | 0.05 | 1.34 | 1.55 | 0.00 |
| Self-Employed | 1.76 | 0.13 | 1.51 | 2.02 | . |
| Retired | 1.01 | 0.07 | 0.87 | 1.15 | . |
| Student | 2.64 | 0.16 | 2.33 | 2.95 | . |
| Homemaker | 1.05 | 0.15 | 0.77 | 1.34 | . |
| Unemployed and Looking for a Job | 0.98 | 0.14 | 0.71 | 1.26 | . |
| None of These/Other | 1.27 | 0.24 | 0.79 | 1.76 | . |
| Education |  |  |  |  |  |
| Up to 8 Years | 1.04 | 0.12 | 0.80 | 1.29 | 0.00 |
| 9-15 Years | 1.33 | 0.06 | 1.21 | 1.44 | . |
| 16+ Years | 1.67 | 0.06 | 1.55 | 1.79 | . |
| Service Attendance |  |  |  |  |  |
| >1/Week | 0.90 | 0.19 | 0.54 | 1.27 | 0.00 |
| 1/Week | 0.91 | 0.05 | 0.80 | 1.01 | . |
| 1-3/Month | 1.24 | 0.08 | 1.09 | 1.40 | . |
| A Few Times a Year | 1.74 | 0.06 | 1.62 | 1.87 | . |
| Never | 1.99 | 0.09 | 1.82 | 2.16 | . |
| Immigration Status |  |  |  |  |  |
| Born in This Country | 1.39 | 0.05 | 1.30 | 1.49 | 0.463 |
| Born in Another Country | 1.21 | 0.26 | 0.70 | 1.73 | . |
| Religion |  |  |  |  |  |
| Christianity | 1.34 | 0.05 | 1.24 | 1.44 | 0.00 |
| Islam | 1.01 | . | . |  | . |
| Buddhism | 0.42 | 0.37 | -1.33 | 2.17 | . |
| Sikhism | 0.00 | . | . |  | . |
| Jainism | 0.26 | . | . | . | . |
| Shinto | 0.00 | . | . | . | . |
| Primal, Animist, or Folk Religion | 2.71 | 0.20 | 2.24 | 3.18 | . |
| No Religion/Atheist/Agnostic | 1.88 | 0.09 | 1.69 | 2.07 | . |
| Race/Ethnicity |  |  |  |  |  |
| Polish | 1.39 | 0.05 | 1.29 | 1.48 | 0.868 |
| German | 1.33 | . | . |  | . |
| Belarussian | 1.43 | . | . |  | . |
| Ukranian | 2.25 | 1.07 | 0.08 | 4.43 | . |
| Silesia | 1.60 | 0.44 | 0.63 | 2.57 | . |
| Kashubians | 2.77 | . | . | . | . |
| Other | 1.61 | 0.85 | -1.30 | 4.53 | . |

**Table 16a: Nationally Representative Descriptive Statistics of the Observed Sample (South_Africa)**

| Variable | Proportion | Frequency |
| --- | --- | --- |
| Age |  |  |
| 18-24 | 0.17 | 461 |
| 25-29 | 0.14 | 364 |
| 30-39 | 0.25 | 655 |
| 40-49 | 0.20 | 522 |
| 50-59 | 0.12 | 309 |
| 60-69 | 0.07 | 195 |
| 70-79 | 0.05 | 120 |
| 80 or Older | 0.01 | 17 |
| Missing | 0.00 | 9 |
| Gender |  |  |
| Male | 0.49 | 1288 |
| Female | 0.51 | 1356 |
| Other | 0.00 | 2 |
| Missing | 0.00 | 4 |
| Marital Status |  |  |
| Single/Never Been Married | 0.59 | 1561 |
| Married | 0.20 | 539 |
| Separated | 0.03 | 76 |
| Divorced | 0.02 | 51 |
| Widowed | 0.05 | 133 |
| Domestic Partner | 0.10 | 264 |
| Missing | 0.01 | 28 |
| Employment |  |  |
| Employed for an Employer | 0.21 | 569 |
| Self-Employed | 0.16 | 412 |
| Retired | 0.09 | 243 |
| Student | 0.08 | 204 |
| Homemaker | 0.05 | 137 |
| Unemployed and Looking for a Job | 0.38 | 1008 |
| None of These/Other | 0.03 | 74 |
| Missing | 0.00 | 3 |
| Education |  |  |
| Up to 8 Years | 0.25 | 668 |
| 9-15 Years | 0.68 | 1796 |
| 16+ Years | 0.07 | 183 |
| Missing | 0.00 | 4 |
| Service Attendance |  |  |
| >1/Week | 0.16 | 414 |
| 1/Week | 0.34 | 891 |
| 1-3/Month | 0.22 | 574 |
| A Few Times a Year | 0.16 | 431 |
| Never | 0.13 | 334 |
| Missing | 0.00 | 7 |
| Immigration Status |  |  |
| Born in This Country | 0.95 | 2511 |
| Born in Another Country | 0.05 | 139 |
| Missing | 0.00 | 1 |
| Religion |  |  |
| Christianity | 0.82 | 2163 |
| Islam | 0.02 | 62 |
| Hinduism | 0.00 | 1 |
| Buddhism | 0.00 | 12 |
| Judaism | . | . |
| Sikhism | . | . |
| Baha'i | . | . |
| Jainism | 0.00 | 2 |
| Shinto | 0.00 | 2 |
| Taoism | 0.00 | 1 |
| Confucianism | . | . |
| Primal, Animist, or Folk Religion | 0.05 | 127 |
| Spiritism | . | . |
| African-Derived | . | . |
| Chinese | . | . |
| Some Other Religion | 0.00 | 5 |
| No Religion/Atheist/Agnostic | 0.10 | 253 |
| Missing | 0.01 | 23 |
| Race/Ethnicity |  |  |
| Black | 0.90 | 2381 |
| Asian/Indian | 0.00 | 6 |
| Colored | 0.10 | 252 |
| White | 0.00 | 8 |
| Other | 0.00 | 1 |
| Missing | 0.00 | 3 |

**Table 16b: Variations Across Demographic Characteristics (South_Africa)**

| Variable | Mean | SE | LCI | UCI | Global p-value |
| --- | --- | --- | --- | --- | --- |
| Age |  |  |  |  |  |
| 18-24 | 2.75 | 0.13 | 2.49 | 3.00 | 0.010 |
| 25-29 | 2.26 | 0.13 | 2.00 | 2.52 | . |
| 30-39 | 2.32 | 0.11 | 2.11 | 2.54 | . |
| 40-49 | 2.06 | 0.15 | 1.77 | 2.36 | . |
| 50-59 | 2.28 | 0.18 | 1.93 | 2.63 | . |
| 60-69 | 2.53 | 0.35 | 1.83 | 3.22 | . |
| 70-79 | 2.14 | 0.40 | 1.33 | 2.95 | . |
| 80 or Older | 0.99 | 0.86 | -1.08 | 3.06 | . |
| Gender |  |  |  |  |  |
| Male | 2.63 | 0.10 | 2.43 | 2.83 | 0.00 |
| Female | 2.04 | 0.09 | 1.87 | 2.21 | . |
| Other | 3.40 | . | . |  | . |
| Marital Status |  |  |  |  |  |
| Single/Never Been Married | 2.37 | 0.07 | 2.23 | 2.52 | 0.608 |
| Married | 2.31 | 0.20 | 1.93 | 2.70 | . |
| Separated | 2.72 | 0.34 | 2.04 | 3.41 | . |
| Divorced | 2.48 | 0.58 | 1.32 | 3.63 | . |
| Widowed | 2.10 | 0.31 | 1.48 | 2.73 | . |
| Domestic Partner | 2.08 | 0.20 | 1.69 | 2.47 | . |
| Employment |  |  |  |  |  |
| Employed for an Employer | 2.14 | 0.13 | 1.89 | 2.39 | 0.083 |
| Self-Employed | 2.45 | 0.16 | 2.13 | 2.77 | . |
| Retired | 2.44 | 0.28 | 1.88 | 3.01 | . |
| Student | 2.56 | 0.18 | 2.20 | 2.91 | . |
| Homemaker | 1.79 | 0.27 | 1.26 | 2.33 | . |
| Unemployed and Looking for a Job | 2.38 | 0.10 | 2.19 | 2.57 | . |
| None of These/Other | 2.44 | 0.45 | 1.55 | 3.33 | . |
| Education |  |  |  |  |  |
| Up to 8 Years | 2.20 | 0.17 | 1.85 | 2.54 | 0.197 |
| 9-15 Years | 2.35 | 0.07 | 2.21 | 2.48 | . |
| 16+ Years | 2.65 | 0.19 | 2.28 | 3.02 | . |
| Service Attendance |  |  |  |  |  |
| >1/Week | 2.52 | 0.17 | 2.18 | 2.85 | 0.067 |
| 1/Week | 2.51 | 0.11 | 2.30 | 2.73 | . |
| 1-3/Month | 2.23 | 0.14 | 1.96 | 2.49 | . |
| A Few Times a Year | 2.12 | 0.13 | 1.86 | 2.38 | . |
| Never | 2.05 | 0.18 | 1.68 | 2.41 | . |
| Immigration Status |  |  |  |  |  |
| Born in This Country | 2.34 | 0.07 | 2.21 | 2.47 | 0.520 |
| Born in Another Country | 2.10 | 0.38 | 1.35 | 2.85 | . |
| Religion |  |  |  |  |  |
| Christianity | 2.34 | 0.07 | 2.20 | 2.48 | 0.00 |
| Islam | 1.61 | 0.33 | 0.93 | 2.28 | . |
| Hinduism | 3.00 | . | . |  | . |
| Buddhism | 1.65 | 0.57 | 0.32 | 2.98 | . |
| Jainism | 5.00 | . | . |  | . |
| Shinto | 3.51 | . | . |  | . |
| Taoism | 3.00 | . | . |  | . |
| Primal, Animist, or Folk Religion | 2.41 | 0.34 | 1.73 | 3.10 | . |
| Some Other Religion | 3.87 | 1.08 | 0.86 | 6.89 | . |
| No Religion/Atheist/Agnostic | 2.35 | 0.18 | 1.99 | 2.71 | . |
| Race/Ethnicity |  |  |  |  |  |
| Black | 2.33 | 0.07 | 2.20 | 2.46 | 0.00 |
| Asian/Indian | 6.42 | . | . |  | . |
| Colored | 2.24 | 0.27 | 1.72 | 2.77 | . |
| White | 2.12 | 0.38 | 1.21 | 3.03 | . |
| Other | 7.00 | . | . |  | . |

**Table 17a: Nationally Representative Descriptive Statistics of the Observed Sample (Spain)**

| Variable | Proportion | Frequency |
| --- | --- | --- |
| Age |  |  |
| 18-24 | 0.09 | 594 |
| 25-29 | 0.07 | 450 |
| 30-39 | 0.18 | 1111 |
| 40-49 | 0.22 | 1396 |
| 50-59 | 0.20 | 1252 |
| 60-69 | 0.16 | 977 |
| 70-79 | 0.07 | 467 |
| 80 or Older | 0.01 | 43 |
| Missing | . | . |
| Gender |  |  |
| Male | 0.50 | 3142 |
| Female | 0.50 | 3119 |
| Other | 0.00 | 6 |
| Missing | 0.00 | 22 |
| Marital Status |  |  |
| Single/Never Been Married | 0.28 | 1742 |
| Married | 0.47 | 2947 |
| Separated | 0.04 | 237 |
| Divorced | 0.08 | 518 |
| Widowed | 0.03 | 189 |
| Domestic Partner | 0.09 | 589 |
| Missing | 0.01 | 67 |
| Employment |  |  |
| Employed for an Employer | 0.45 | 2862 |
| Self-Employed | 0.09 | 576 |
| Retired | 0.20 | 1278 |
| Student | 0.07 | 448 |
| Homemaker | 0.05 | 345 |
| Unemployed and Looking for a Job | 0.10 | 646 |
| None of These/Other | 0.02 | 123 |
| Missing | 0.00 | 11 |
| Education |  |  |
| Up to 8 Years | 0.13 | 802 |
| 9-15 Years | 0.66 | 4145 |
| 16+ Years | 0.21 | 1341 |
| Missing | 0.00 | 2 |
| Service Attendance |  |  |
| >1/Week | 0.05 | 317 |
| 1/Week | 0.11 | 662 |
| 1-3/Month | 0.07 | 437 |
| A Few Times a Year | 0.31 | 1972 |
| Never | 0.46 | 2875 |
| Missing | 0.00 | 27 |
| Immigration Status |  |  |
| Born in This Country | 0.87 | 5479 |
| Born in Another Country | 0.13 | 788 |
| Missing | 0.00 | 23 |
| Religion |  |  |
| Christianity | 0.65 | 4074 |
| Islam | 0.02 | 135 |
| Hinduism | 0.00 | 7 |
| Buddhism | 0.01 | 36 |
| Judaism | 0.00 | 4 |
| Sikhism | 0.00 | 3 |
| Baha'i | 0.00 | 2 |
| Jainism | 0.00 | 1 |
| Shinto | . | . |
| Taoism | 0.00 | 5 |
| Confucianism | 0.00 | 3 |
| Primal, Animist, or Folk Religion | 0.00 | 7 |
| Spiritism | . | . |
| African-Derived | . | . |
| Chinese | . | . |
| Some Other Religion | 0.00 | 27 |
| No Religion/Atheist/Agnostic | 0.31 | 1932 |
| Missing | 0.01 | 55 |
| Race/Ethnicity |  |  |
| No Data | . | . |

**Table 17b: Variations Across Demographic Characteristics (Spain)**

| Variable | Mean | SE | LCI | UCI | Global p-value |
| --- | --- | --- | --- | --- | --- |
| Age |  |  |  |  |  |
| 18-24 | 2.93 | 0.12 | 2.69 | 3.16 | 0.155 |
| 25-29 | 2.52 | 0.12 | 2.28 | 2.75 | . |
| 30-39 | 2.63 | 0.07 | 2.48 | 2.77 | . |
| 40-49 | 2.67 | 0.07 | 2.54 | 2.80 | . |
| 50-59 | 2.54 | 0.09 | 2.37 | 2.71 | . |
| 60-69 | 2.85 | 0.14 | 2.59 | 3.12 | . |
| 70-79 | 2.72 | 0.24 | 2.25 | 3.19 | . |
| 80 or Older | 2.60 | 0.64 | 1.29 | 3.91 | . |
| Gender |  |  |  |  |  |
| Male | 2.87 | 0.06 | 2.75 | 2.98 | 0.00 |
| Female | 2.50 | 0.06 | 2.38 | 2.61 | . |
| Other | 3.90 | 0.52 | 2.76 | 5.05 | . |
| Marital Status |  |  |  |  |  |
| Single/Never Been Married | 2.70 | 0.07 | 2.56 | 2.83 | 0.040 |
| Married | 2.65 | 0.06 | 2.53 | 2.77 | . |
| Separated | 2.87 | 0.20 | 2.48 | 3.27 | . |
| Divorced | 2.94 | 0.16 | 2.62 | 3.26 | . |
| Widowed | 1.89 | 0.29 | 1.31 | 2.46 | . |
| Domestic Partner | 2.73 | 0.12 | 2.50 | 2.96 | . |
| Employment |  |  |  |  |  |
| Employed for an Employer | 2.74 | 0.05 | 2.64 | 2.84 | 0.189 |
| Self-Employed | 2.66 | 0.11 | 2.45 | 2.88 | . |
| Retired | 2.69 | 0.13 | 2.44 | 2.94 | . |
| Student | 2.83 | 0.14 | 2.56 | 3.11 | . |
| Homemaker | 2.41 | 0.19 | 2.03 | 2.78 | . |
| Unemployed and Looking for a Job | 2.55 | 0.11 | 2.34 | 2.76 | . |
| None of These/Other | 2.29 | 0.24 | 1.81 | 2.76 | . |
| Education |  |  |  |  |  |
| Up to 8 Years | 2.41 | 0.14 | 2.13 | 2.68 | 0.071 |
| 9-15 Years | 2.74 | 0.05 | 2.64 | 2.84 | . |
| 16+ Years | 2.67 | 0.08 | 2.52 | 2.82 | . |
| Service Attendance |  |  |  |  |  |
| >1/Week | 2.98 | 0.18 | 2.62 | 3.33 | 0.001 |
| 1/Week | 2.90 | 0.13 | 2.64 | 3.16 | . |
| 1-3/Month | 3.13 | 0.15 | 2.83 | 3.43 | . |
| A Few Times a Year | 2.58 | 0.07 | 2.44 | 2.72 | . |
| Never | 2.60 | 0.06 | 2.49 | 2.72 | . |
| Immigration Status |  |  |  |  |  |
| Born in This Country | 2.72 | 0.04 | 2.63 | 2.81 | 0.006 |
| Born in Another Country | 2.43 | 0.09 | 2.24 | 2.62 | . |
| Religion |  |  |  |  |  |
| Christianity | 2.72 | 0.05 | 2.62 | 2.83 | 0.00 |
| Islam | 2.50 | 0.22 | 2.07 | 2.94 | . |
| Hinduism | 2.82 | 0.52 | 1.60 | 4.04 | . |
| Buddhism | 3.10 | 0.43 | 2.24 | 3.97 | . |
| Judaism | 3.55 | 0.43 | 2.31 | 4.79 | . |
| Sikhism | 1.93 | 0.90 | -1.36 | 5.22 | . |
| Baha'i | 2.40 | . | . | . | . |
| Jainism | 0.00 | . | . | . | . |
| Taoism | 4.23 | 1.41 | -2.53 | 10.98 | . |
| Confucianism | 0.97 | 0.82 | -2.95 | 4.90 | . |
| Primal, Animist, or Folk Religion | 2.33 | 0.57 | 1.10 | 3.56 | . |
| Some Other Religion | 3.38 | 0.90 | 1.55 | 5.21 | . |
| No Religion/Atheist/Agnostic | 2.59 | 0.07 | 2.46 | 2.73 | . |
| Race/Ethnicity |  |  |  |  |  |
| No Data | . | . | . |  | . |

**Table 18a: Nationally Representative Descriptive Statistics of the Observed Sample (Sweden)**

| Variable | Proportion | Frequency |
| --- | --- | --- |
| Age |  |  |
| 18-24 | 0.10 | 1515 |
| 25-29 | 0.09 | 1399 |
| 30-39 | 0.16 | 2398 |
| 40-49 | 0.15 | 2221 |
| 50-59 | 0.17 | 2493 |
| 60-69 | 0.14 | 2168 |
| 70-79 | 0.15 | 2253 |
| 80 or Older | 0.04 | 621 |
| Missing | . | . |
| Gender |  |  |
| Male | 0.50 | 7536 |
| Female | 0.50 | 7493 |
| Other | 0.00 | 27 |
| Missing | 0.00 | 12 |
| Marital Status |  |  |
| Single/Never Been Married | 0.26 | 3854 |
| Married | 0.43 | 6408 |
| Separated | 0.03 | 426 |
| Divorced | 0.05 | 801 |
| Widowed | 0.03 | 433 |
| Domestic Partner | 0.20 | 3073 |
| Missing | 0.00 | 72 |
| Employment |  |  |
| Employed for an Employer | 0.52 | 7907 |
| Self-Employed | 0.08 | 1243 |
| Retired | 0.25 | 3832 |
| Student | 0.09 | 1332 |
| Homemaker | 0.00 | 75 |
| Unemployed and Looking for a Job | 0.02 | 324 |
| None of These/Other | 0.02 | 337 |
| Missing | 0.00 | 18 |
| Education |  |  |
| Up to 8 Years | 0.02 | 252 |
| 9-15 Years | 0.72 | 10790 |
| 16+ Years | 0.27 | 4026 |
| Missing | . | . |
| Service Attendance |  |  |
| >1/Week | 0.02 | 236 |
| 1/Week | 0.03 | 434 |
| 1-3/Month | 0.03 | 486 |
| A Few Times a Year | 0.26 | 3950 |
| Never | 0.66 | 9918 |
| Missing | 0.00 | 45 |
| Immigration Status |  |  |
| Born in This Country | 0.92 | 13922 |
| Born in Another Country | 0.07 | 1052 |
| Missing | 0.01 | 94 |
| Religion |  |  |
| Christianity | 0.55 | 8346 |
| Islam | 0.03 | 470 |
| Hinduism | 0.00 | 22 |
| Buddhism | 0.01 | 110 |
| Judaism | 0.00 | 54 |
| Sikhism | 0.00 | 4 |
| Baha'i | 0.00 | 6 |
| Jainism | . | . |
| Shinto | 0.00 | 0 |
| Taoism | 0.00 | 4 |
| Confucianism | . | . |
| Primal, Animist, or Folk Religion | 0.01 | 83 |
| Spiritism | . | . |
| African-Derived | . | . |
| Chinese | . | . |
| Some Other Religion | 0.01 | 198 |
| No Religion/Atheist/Agnostic | 0.38 | 5697 |
| Missing | 0.00 | 74 |
| Race/Ethnicity |  |  |
| No Data | . | . |

**Table 18b: Variations Across Demographic Characteristics (Sweden)**

| Variable | Mean | SE | LCI | UCI | Global p-value |
| --- | --- | --- | --- | --- | --- |
| Age |  |  |  |  |  |
| 18-24 | 3.25 | 0.06 | 3.13 | 3.37 | 0.00 |
| 25-29 | 2.94 | 0.07 | 2.81 | 3.08 | . |
| 30-39 | 2.65 | 0.05 | 2.54 | 2.75 | . |
| 40-49 | 2.47 | 0.06 | 2.35 | 2.59 | . |
| 50-59 | 2.49 | 0.05 | 2.38 | 2.59 | . |
| 60-69 | 2.83 | 0.06 | 2.71 | 2.94 | . |
| 70-79 | 3.02 | 0.06 | 2.90 | 3.15 | . |
| 80 or Older | 2.76 | 0.11 | 2.54 | 2.99 | . |
| Gender |  |  |  |  |  |
| Male | 2.78 | 0.03 | 2.71 | 2.84 | 0.624 |
| Female | 2.77 | 0.03 | 2.70 | 2.83 | . |
| Other | 2.29 | 0.51 | 1.26 | 3.33 | . |
| Marital Status |  |  |  |  |  |
| Single/Never Been Married | 2.89 | 0.05 | 2.80 | 2.98 | 0.002 |
| Married | 2.75 | 0.04 | 2.68 | 2.82 | . |
| Separated | 2.86 | 0.12 | 2.62 | 3.09 | . |
| Divorced | 2.65 | 0.10 | 2.46 | 2.84 | . |
| Widowed | 2.94 | 0.14 | 2.67 | 3.21 | . |
| Domestic Partner | 2.65 | 0.04 | 2.56 | 2.73 | . |
| Employment |  |  |  |  |  |
| Employed for an Employer | 2.66 | 0.03 | 2.60 | 2.71 | 0.00 |
| Self-Employed | 2.82 | 0.09 | 2.63 | 3.00 | . |
| Retired | 2.93 | 0.05 | 2.84 | 3.02 | . |
| Student | 3.06 | 0.07 | 2.93 | 3.20 | . |
| Homemaker | 2.43 | 0.33 | 1.78 | 3.08 | . |
| Unemployed and Looking for a Job | 2.60 | 0.13 | 2.34 | 2.86 | . |
| None of These/Other | 2.51 | 0.15 | 2.21 | 2.80 | . |
| Education |  |  |  |  |  |
| Up to 8 Years | 2.77 | 0.19 | 2.39 | 3.16 | 0.007 |
| 9-15 Years | 2.73 | 0.03 | 2.68 | 2.78 | . |
| 16+ Years | 2.89 | 0.04 | 2.80 | 2.97 | . |
| Service Attendance |  |  |  |  |  |
| >1/Week | 2.83 | 0.19 | 2.45 | 3.20 | 0.00 |
| 1/Week | 3.02 | 0.14 | 2.73 | 3.30 | . |
| 1-3/Month | 2.97 | 0.12 | 2.73 | 3.22 | . |
| A Few Times a Year | 2.92 | 0.04 | 2.83 | 3.01 | . |
| Never | 2.69 | 0.03 | 2.64 | 2.74 | . |
| Immigration Status |  |  |  |  |  |
| Born in This Country | 2.77 | 0.02 | 2.73 | 2.82 | 0.647 |
| Born in Another Country | 2.73 | 0.09 | 2.56 | 2.90 | . |
| Religion |  |  |  |  |  |
| Christianity | 2.84 | 0.03 | 2.78 | 2.90 | 0.00 |
| Islam | 2.85 | 0.15 | 2.56 | 3.14 | . |
| Hinduism | 3.55 | 0.69 | 1.96 | 5.14 | . |
| Buddhism | 2.93 | 0.34 | 2.24 | 3.61 | . |
| Judaism | 2.36 | 0.49 | 1.36 | 3.37 | . |
| Sikhism | 3.88 | . | . |  | . |
| Baha'i | 2.10 | 1.62 | -5.69 | 9.89 | . |
| Shinto | 1.00 | . | . |  | . |
| Taoism | 0.60 | . | . |  | . |
| Primal, Animist, or Folk Religion | 2.06 | 0.38 | 1.30 | 2.83 | . |
| Some Other Religion | 2.51 | 0.24 | 2.04 | 2.97 | . |
| No Religion/Atheist/Agnostic | 2.68 | 0.03 | 2.62 | 2.74 | . |
| Race/Ethnicity |  |  |  |  |  |
| No Data | . | . | . | . | . |

**Table 19a: Nationally Representative Descriptive Statistics of the Observed Sample (Tanzania)**

| Variable | Proportion | Frequency |
| --- | --- | --- |
| Age |  |  |
| 18-24 | 0.25 | 2284 |
| 25-29 | 0.15 | 1349 |
| 30-39 | 0.23 | 2060 |
| 40-49 | 0.17 | 1503 |
| 50-59 | 0.10 | 912 |
| 60-69 | 0.06 | 575 |
| 70-79 | 0.03 | 297 |
| 80 or Older | 0.01 | 93 |
| Missing | 0.00 | 2 |
| Gender |  |  |
| Male | 0.47 | 4299 |
| Female | 0.53 | 4776 |
| Other | . | . |
| Missing | . | . |
| Marital Status |  |  |
| Single/Never Been Married | 0.25 | 2260 |
| Married | 0.61 | 5577 |
| Separated | 0.04 | 404 |
| Divorced | 0.01 | 103 |
| Widowed | 0.05 | 450 |
| Domestic Partner | 0.03 | 275 |
| Missing | 0.00 | 7 |
| Employment |  |  |
| Employed for an Employer | 0.06 | 513 |
| Self-Employed | 0.51 | 4625 |
| Retired | 0.02 | 139 |
| Student | 0.04 | 319 |
| Homemaker | 0.20 | 1796 |
| Unemployed and Looking for a Job | 0.16 | 1491 |
| None of These/Other | 0.02 | 186 |
| Missing | 0.00 | 6 |
| Education |  |  |
| Up to 8 Years | 0.74 | 6699 |
| 9-15 Years | 0.25 | 2252 |
| 16+ Years | 0.01 | 122 |
| Missing | 0.00 | 2 |
| Service Attendance |  |  |
| >1/Week | 0.29 | 2622 |
| 1/Week | 0.47 | 4268 |
| 1-3/Month | 0.12 | 1082 |
| A Few Times a Year | 0.09 | 814 |
| Never | 0.03 | 288 |
| Missing | 0.00 | 1 |
| Immigration Status |  |  |
| Born in This Country | 1.00 | 9048 |
| Born in Another Country | 0.00 | 25 |
| Missing | 0.00 | 1 |
| Religion |  |  |
| Christianity | 0.62 | 5647 |
| Islam | 0.35 | 3189 |
| Hinduism | . | . |
| Buddhism | . | . |
| Judaism | . | . |
| Sikhism | . | . |
| Baha'i | . | . |
| Jainism | . | . |
| Shinto | . | . |
| Taoism | 0.00 | 1 |
| Confucianism | . | . |
| Primal, Animist, or Folk Religion | 0.00 | 12 |
| Spiritism | . | . |
| African-Derived | . | . |
| Chinese | . | . |
| Some Other Religion | . | . |
| No Religion/Atheist/Agnostic | 0.02 | 216 |
| Missing | 0.00 | 10 |
| Race/Ethnicity |  |  |
| African | 1.00 | 9060 |
| Indian | 0.00 | 3 |
| Arab | 0.00 | 11 |
| Other | . | . |
| Missing | 0.00 | 2 |

**Table 19b: Variations Across Demographic Characteristics (Tanzania)**

| Variable | Mean | SE | LCI | UCI | Global p-value |
| --- | --- | --- | --- | --- | --- |
| Age |  |  |  |  |  |
| 18-24 | 3.13 | 0.09 | 2.95 | 3.31 | 0.00 |
| 25-29 | 3.15 | 0.10 | 2.96 | 3.35 | . |
| 30-39 | 3.26 | 0.09 | 3.08 | 3.45 | . |
| 40-49 | 3.55 | 0.10 | 3.35 | 3.75 | . |
| 50-59 | 3.43 | 0.12 | 3.20 | 3.67 | . |
| 60-69 | 3.39 | 0.16 | 3.08 | 3.70 | . |
| 70-79 | 2.79 | 0.27 | 2.25 | 3.32 | . |
| 80 or Older | 2.56 | 0.34 | 1.87 | 3.25 | . |
| Gender |  |  |  |  |  |
| Male | 3.98 | 0.07 | 3.83 | 4.12 | 0.00 |
| Female | 2.62 | 0.08 | 2.47 | 2.78 | . |
| Other | . | . | . |  | . |
| Marital Status |  |  |  |  |  |
| Single/Never Been Married | 3.16 | 0.09 | 2.99 | 3.32 | 0.00 |
| Married | 3.40 | 0.08 | 3.25 | 3.55 | . |
| Separated | 3.24 | 0.18 | 2.90 | 3.59 | . |
| Divorced | 2.46 | 0.29 | 1.88 | 3.05 | . |
| Widowed | 2.63 | 0.19 | 2.27 | 3.00 | . |
| Domestic Partner | 2.74 | 0.20 | 2.35 | 3.14 | . |
| Employment |  |  |  |  |  |
| Employed for an Employer | 3.34 | 0.15 | 3.05 | 3.63 | 0.001 |
| Self-Employed | 3.57 | 0.08 | 3.41 | 3.73 | . |
| Retired | 2.75 | 0.29 | 2.17 | 3.33 | . |
| Student | 3.18 | 0.19 | 2.81 | 3.54 | . |
| Homemaker | 2.61 | 0.11 | 2.40 | 2.83 | . |
| Unemployed and Looking for a Job | 3.11 | 0.10 | 2.92 | 3.31 | . |
| None of These/Other | 3.55 | 0.22 | 3.12 | 3.99 | . |
| Education |  |  |  |  |  |
| Up to 8 Years | 3.26 | 0.08 | 3.11 | 3.41 | 0.832 |
| 9-15 Years | 3.29 | 0.08 | 3.13 | 3.44 | . |
| 16+ Years | 3.15 | 0.23 | 2.69 | 3.61 | . |
| Service Attendance |  |  |  |  |  |
| >1/Week | 3.35 | 0.09 | 3.17 | 3.52 | 0.677 |
| 1/Week | 3.22 | 0.08 | 3.06 | 3.38 | . |
| 1-3/Month | 3.24 | 0.12 | 3.00 | 3.48 | . |
| A Few Times a Year | 3.33 | 0.12 | 3.09 | 3.57 | . |
| Never | 3.09 | 0.27 | 2.55 | 3.62 | . |
| Immigration Status |  |  |  |  |  |
| Born in This Country | 3.27 | 0.07 | 3.14 | 3.40 | 0.054 |
| Born in Another Country | 2.33 | 0.51 | 1.27 | 3.38 | . |
| Religion |  |  |  |  |  |
| Christianity | 3.29 | 0.08 | 3.15 | 3.44 | 0.00 |
| Islam | 3.17 | 0.10 | 2.97 | 3.37 | . |
| Taoism | 7.00 | . | . |  | . |
| Primal, Animist, or Folk Religion | 3.66 | 0.29 | 3.00 | 4.13 | . |
| No Religion/Atheist/Agnostic | 3.81 | 0.30 | 3.22 | 4.39 | . |
| Race/Ethnicity |  |  |  |  |  |
| African | 3.26 | 0.07 | 3.14 | 3.39 | 0.824 |
| Indian | 3.36 | . | . | . | . |
| Arab | 2.66 | 1.13 | 0.02 | 5.30 | . |

**Table 20a: Nationally Representative Descriptive Statistics of the Observed Sample (Turkey)**

| Variable | Proportion | Frequency |
| --- | --- | --- |
| Age |  |  |
| 18-24 | 0.15 | 222 |
| 25-29 | 0.10 | 152 |
| 30-39 | 0.21 | 315 |
| 40-49 | 0.21 | 312 |
| 50-59 | 0.15 | 225 |
| 60-69 | 0.11 | 164 |
| 70-79 | 0.04 | 65 |
| 80 or Older | 0.01 | 18 |
| Missing | . | . |
| Gender |  |  |
| Male | 0.51 | 754 |
| Female | 0.49 | 719 |
| Other | . | . |
| Missing | . | . |
| Marital Status |  |  |
| Single/Never Been Married | 0.26 | 379 |
| Married | 0.64 | 936 |
| Separated | 0.01 | 13 |
| Divorced | 0.04 | 64 |
| Widowed | 0.04 | 64 |
| Domestic Partner | . | . |
| Missing | 0.01 | 17 |
| Employment |  |  |
| Employed for an Employer | 0.28 | 413 |
| Self-Employed | 0.17 | 255 |
| Retired | 0.14 | 205 |
| Student | 0.07 | 107 |
| Homemaker | 0.24 | 347 |
| Unemployed and Looking for a Job | 0.06 | 87 |
| None of These/Other | 0.04 | 59 |
| Missing | . | . |
| Education |  |  |
| Up to 8 Years | 0.30 | 436 |
| 9-15 Years | 0.48 | 711 |
| 16+ Years | 0.22 | 326 |
| Missing | . | . |
| Service Attendance |  |  |
| >1/Week | 0.33 | 493 |
| 1/Week | 0.18 | 271 |
| 1-3/Month | 0.12 | 174 |
| A Few Times a Year | 0.17 | 255 |
| Never | 0.19 | 274 |
| Missing | 0.00 | 6 |
| Immigration Status |  |  |
| Born in This Country | 0.96 | 1415 |
| Born in Another Country | 0.04 | 58 |
| Missing | . | . |
| Religion |  |  |
| Christianity | 0.00 | 2 |
| Islam | 0.94 | 1381 |
| Hinduism | . | . |
| Buddhism | 0.00 | 1 |
| Judaism | 0.00 | 1 |
| Sikhism | 0.00 | 1 |
| Baha'i | . | . |
| Jainism | . | . |
| Shinto | . | . |
| Taoism | . | . |
| Confucianism | . | . |
| Primal, Animist, or Folk Religion | 0.00 | 1 |
| Spiritism | . | . |
| African-Derived | . | . |
| Chinese | . | . |
| Some Other Religion | 0.00 | 1 |
| No Religion/Atheist/Agnostic | 0.04 | 66 |
| Missing | 0.01 | 19 |
| Race/Ethnicity |  |  |
| Turkish | 0.70 | 1030 |
| Kurdish/Zaza | 0.17 | 252 |
| Arab | 0.03 | 51 |
| Laz | 0.02 | 25 |
| Circassian | 0.01 | 19 |
| Bosnian | 0.00 | 5 |
| Armenian | 0.00 | 1 |
| Georgian | 0.00 | 4 |
| Uyghur | 0.00 | 1 |
| Jewish | . | . |
| Albanian | 0.01 | 8 |
| Greek | 0.00 | 1 |
| Azeri | 0.01 | 9 |
| Other | 0.04 | 58 |
| Missing | 0.01 | 9 |

**Table 20b: Variations Across Demographic Characteristics (Turkey)**

| Variable | Mean | SE | LCI | UCI | Global p-value |
| --- | --- | --- | --- | --- | --- |
| Age |  |  |  |  |  |
| 18-24 | 2.71 | 0.18 | 2.36 | 3.06 | 0.00 |
| 25-29 | 2.92 | 0.26 | 2.40 | 3.44 | . |
| 30-39 | 2.39 | 0.16 | 2.07 | 2.71 | . |
| 40-49 | 2.64 | 0.18 | 2.29 | 2.99 | . |
| 50-59 | 3.20 | 0.25 | 2.70 | 3.70 | . |
| 60-69 | 3.74 | 0.35 | 3.05 | 4.43 | . |
| 70-79 | 2.54 | 0.62 | 1.25 | 3.93 | . |
| 80 or Older | 0.47 | 0.52 | -0.85 | 1.78 | . |
| Gender |  |  |  |  |  |
| Male | 2.85 | 0.12 | 2.61 | 3.09 | 0.601 |
| Female | 2.75 | 0.14 | 2.48 | 3.03 | . |
| Other | . | . | . |  | . |
| Marital Status |  |  |  |  |  |
| Single/Never Been Married | 3.03 | 0.15 | 2.73 | 3.32 | 0.291 |
| Married | 2.71 | 0.12 | 2.48 | 2.85 | . |
| Separated | 3.19 | 0.37 | 2.40 | 3.99 | . |
| Divorced | 2.37 | 0.40 | 1.56 | 3.18 | . |
| Widowed | 3.17 | 0.51 | 2.13 | 4.21 | . |
| Domestic Partner | . | . | . |  | . |
| Employment |  |  |  |  |  |
| Employed for an Employer | 2.78 | 0.15 | 2.49 | 3.08 | 0.136 |
| Self-Employed | 2.95 | 0.21 | 2.55 | 3.35 | . |
| Retired | 3.16 | 0.30 | 2.57 | 3.76 | . |
| Student | 2.62 | 0.19 | 2.24 | 3.01 | . |
| Homemaker | 2.79 | 0.22 | 2.37 | 3.22 | . |
| Unemployed and Looking for a Job | 2.56 | 0.31 | 1.94 | 3.17 | . |
| None of These/Other | 1.82 | 0.41 | 1.01 | 2.64 | . |
| Education |  |  |  |  |  |
| Up to 8 Years | 3.10 | 0.21 | 2.69 | 3.52 | 0.092 |
| 9-15 Years | 2.73 | 0.13 | 2.48 | 2.98 | . |
| 16+ Years | 2.57 | 0.11 | 2.34 | 2.79 | . |
| Service Attendance |  |  |  |  |  |
| >1/Week | 3.26 | 0.17 | 2.92 | 3.60 | 0.00 |
| 1/Week | 2.79 | 0.23 | 2.33 | 3.24 | . |
| 1-3/Month | 3.11 | 0.28 | 2.56 | 3.66 | . |
| A Few Times a Year | 2.20 | 0.18 | 1.84 | 2.56 | . |
| Never | 2.37 | 0.19 | 1.99 | 2.75 | . |
| Immigration Status |  |  |  |  |  |
| Born in This Country | 2.80 | 0.09 | 2.62 | 2.99 | 0.747 |
| Born in Another Country | 2.85 | 0.49 | 1.86 | 3.84 | . |
| Religion |  |  |  |  |  |
| Christianity | 2.25 | 0.04 | 2.06 | 2.44 | 0.00 |
| Islam | 2.80 | 0.10 | 2.61 | 2.99 | . |
| Buddhism | 7.00 | . | . | . | . |
| Judaism | 2.00 | . | . | . | . |
| Sikhism | 7.00 | . | . | . | . |
| Primal, Animist, or Folk Religion | 2.13 | . | . | . | . |
| Some Other Religion | 0.42 | . | . | . | . |
| No Religion/Atheist/Agnostic | 2.85 | 0.32 | 2.19 | 3.50 | . |
| Race/Ethnicity |  |  |  |  |  |
| Turkish | 2.72 | 0.11 | 2.51 | 2.93 | 0.00 |
| Kurdish/Zaza | 2.68 | 0.23 | 2.24 | 3.13 | . |
| Arab | 3.72 | 0.55 | 2.59 | 4.84 | . |
| Laz | 2.38 | 0.66 | 1.01 | 3.75 | . |
| Circassian | 4.15 | 0.76 | 2.55 | 5.75 | . |
| Bosnian | 5.24 | 0.11 | 4.91 | 5.57 | . |
| Armenian | 5.86 | . | . | . | . |
| Georgian | 2.84 | 0.44 | 1.55 | 4.13 | . |
| Uyghur | 7.00 | . | . | . | . |
| Albanian | 2.45 | 0.12 | 2.09 | 2.81 | . |
| Greek | 2.29 | . | . | . | . |
| Azeri | 2.31 | 0.54 | 1.04 | 3.59 | . |
| Other | 3.49 | 0.49 | 2.49 | 4.49 | . |

**Table 21a: Nationally Representative Descriptive Statistics of the Observed Sample (United_Kingdom)**

| Variable | Proportion | Frequency |
| --- | --- | --- |
| Age |  |  |
| 18-24 | 0.09 | 490 |
| 25-29 | 0.07 | 391 |
| 30-39 | 0.18 | 946 |
| 40-49 | 0.15 | 827 |
| 50-59 | 0.18 | 949 |
| 60-69 | 0.17 | 889 |
| 70-79 | 0.13 | 711 |
| 80 or Older | 0.03 | 163 |
| Missing | 0.00 | 1 |
| Gender |  |  |
| Male | 0.48 | 2557 |
| Female | 0.52 | 2789 |
| Other | 0.00 | 14 |
| Missing | 0.00 | 9 |
| Marital Status |  |  |
| Single/Never Been Married | 0.27 | 1456 |
| Married | 0.47 | 2510 |
| Separated | 0.02 | 114 |
| Divorced | 0.08 | 435 |
| Widowed | 0.05 | 294 |
| Domestic Partner | 0.10 | 512 |
| Missing | 0.01 | 48 |
| Employment |  |  |
| Employed for an Employer | 0.52 | 2798 |
| Self-Employed | 0.09 | 469 |
| Retired | 0.24 | 1262 |
| Student | 0.04 | 229 |
| Homemaker | 0.03 | 184 |
| Unemployed and Looking for a Job | 0.04 | 215 |
| None of These/Other | 0.04 | 201 |
| Missing | 0.00 | 11 |
| Education |  |  |
| Up to 8 Years | 0.24 | 1314 |
| 9-15 Years | 0.39 | 2072 |
| 16+ Years | 0.37 | 1974 |
| Missing | 0.00 | 8 |
| Service Attendance |  |  |
| >1/Week | 0.05 | 291 |
| 1/Week | 0.09 | 499 |
| 1-3/Month | 0.05 | 293 |
| A Few Times a Year | 0.22 | 1165 |
| Never | 0.58 | 3110 |
| Missing | 0.00 | 10 |
| Immigration Status |  |  |
| Born in This Country | 0.87 | 4659 |
| Born in Another Country | 0.13 | 682 |
| Missing | 0.00 | 27 |
| Religion |  |  |
| Christianity | 0.51 | 2750 |
| Islam | 0.04 | 218 |
| Hinduism | 0.01 | 61 |
| Buddhism | 0.01 | 30 |
| Judaism | 0.01 | 44 |
| Sikhism | 0.01 | 29 |
| Baha'i | 0.00 | 6 |
| Jainism | 0.00 | 4 |
| Shinto | . | . |
| Taoism | 0.00 | 4 |
| Confucianism | 0.00 | 2 |
| Primal, Animist, or Folk Religion | 0.01 | 36 |
| Spiritism | . | . |
| African-Derived | . | . |
| Chinese | . | . |
| Some Other Religion | 0.01 | 61 |
| No Religion/Atheist/Agnostic | 0.39 | 2099 |
| Missing | 0.00 | 25 |
| Race/Ethnicity |  |  |
| Asian | 0.08 | 426 |
| Black | 0.03 | 152 |
| White | 0.87 | 4647 |
| Other | 0.02 | 96 |
| Missing | 0.01 | 47 |

**Table 21b: Variations Across Demographic Characteristics (United_Kingdom)**

| Variable | Mean | SE | LCI | UCI | Global p-value |
| --- | --- | --- | --- | --- | --- |
| Age |  |  |  |  |  |
| 18-24 | 2.71 | 0.17 | 2.38 | 3.05 | 0.00 |
| 25-29 | 2.89 | 0.16 | 2.58 | 3.20 | . |
| 30-39 | 2.66 | 0.10 | 2.45 | 2.86 | . |
| 40-49 | 2.61 | 0.10 | 2.41 | 2.81 | . |
| 50-59 | 2.73 | 0.11 | 2.51 | 2.94 | . |
| 60-69 | 2.52 | 0.12 | 2.27 | 2.76 | . |
| 70-79 | 1.98 | 0.13 | 1.74 | 2.23 | . |
| 80 or Older | 1.87 | 0.27 | 1.32 | 2.41 | . |
| Gender |  |  |  |  |  |
| Male | 2.70 | 0.07 | 2.56 | 2.83 | 0.005 |
| Female | 2.42 | 0.06 | 2.29 | 2.54 | . |
| Other | 1.91 | 0.49 | 0.83 | 2.98 | . |
| Marital Status |  |  |  |  |  |
| Single/Never Been Married | 2.70 | 0.09 | 2.51 | 2.88 | 0.001 |
| Married | 2.52 | 0.06 | 2.40 | 2.65 | . |
| Separated | 2.39 | 0.22 | 1.96 | 2.82 | . |
| Divorced | 2.24 | 0.18 | 1.89 | 2.59 | . |
| Widowed | 1.97 | 0.19 | 1.60 | 2.34 | . |
| Domestic Partner | 2.88 | 0.16 | 2.57 | 3.19 | . |
| Employment |  |  |  |  |  |
| Employed for an Employer | 2.79 | 0.06 | 2.67 | 2.91 | 0.00 |
| Self-Employed | 3.11 | 0.16 | 2.80 | 3.42 | . |
| Retired | 2.07 | 0.10 | 1.88 | 2.26 | . |
| Student | 2.77 | 0.22 | 2.33 | 3.21 | . |
| Homemaker | 2.16 | 0.27 | 1.63 | 2.69 | . |
| Unemployed and Looking for a Job | 1.87 | 0.24 | 1.39 | 2.35 | . |
| None of These/Other | 1.65 | 0.24 | 1.18 | 2.13 | . |
| Education |  |  |  |  |  |
| Up to 8 Years | 2.21 | 0.13 | 1.94 | 2.47 | 0.00 |
| 9-15 Years | 2.46 | 0.06 | 2.33 | 2.58 | . |
| 16+ Years | 2.87 | 0.06 | 2.76 | 2.98 | . |
| Service Attendance |  |  |  |  |  |
| >1/Week | 2.74 | 0.18 | 2.38 | 3.10 | 0.043 |
| 1/Week | 2.86 | 0.14 | 2.58 | 3.14 | . |
| 1-3/Month | 2.70 | 0.19 | 2.33 | 3.08 | . |
| A Few Times a Year | 2.61 | 0.10 | 2.42 | 2.80 | . |
| Never | 2.44 | 0.06 | 2.32 | 2.56 | . |
| Immigration Status |  |  |  |  |  |
| Born in This Country | 2.52 | 0.05 | 2.43 | 2.69 | 0.184 |
| Born in Another Country | 2.70 | 0.12 | 2.46 | 2.95 | . |
| Religion |  |  |  |  |  |
| Christianity | 2.56 | 0.07 | 2.43 | 2.69 | 0.00 |
| Islam | 2.23 | 0.21 | 1.81 | 2.65 | . |
| Hinduism | 2.59 | 0.36 | 1.86 | 3.33 | . |
| Buddhism | 2.75 | 0.39 | 1.96 | 3.54 | . |
| Judaism | 3.00 | 0.65 | 1.69 | 4.31 | . |
| Sikhism | 3.28 | 0.61 | 1.98 | 4.57 | . |
| Baha'i | 0.00 | 0.00 | 0.00 | 0.00 | . |
| Jainism | 2.00 | . | . | . | . |
| Taoism | 2.34 | 1.45 | -2.61 | 7.30 | . |
| Confucianism | 0.00 | . | . | . | . |
| Primal, Animist, or Folk Religion | 3.00 | 0.73 | 1.48 | 4.53 | . |
| Some Other Religion | 2.88 | 0.43 | 2.02 | 3.73 | . |
| No Religion/Atheist/Agnostic | 2.54 | 0.07 | 2.39 | 2.68 | . |
| Race/Ethnicity |  |  |  |  |  |
| Asian | 2.36 | 0.16 | 2.05 | 2.67 | 0.220 |
| Black | 2.71 | 0.20 | 2.32 | 3.11 | . |
| White | 2.55 | 0.05 | 2.45 | 2.65 | . |
| Other | 3.15 | 0.40 | 2.36 | 3.94 | . |

**Table 22a: Nationally Representative Descriptive Statistics of the Observed Sample (United_States)**

| Variable | Proportion | Frequency |
| --- | --- | --- |
| Age |  |  |
| 18-24 | 0.07 | 2682 |
| 25-29 | 0.09 | 3540 |
| 30-39 | 0.19 | 7284 |
| 40-49 | 0.15 | 5649 |
| 50-59 | 0.18 | 6745 |
| 60-69 | 0.18 | 6832 |
| 70-79 | 0.11 | 4054 |
| 80 or Older | 0.04 | 1525 |
| Missing | . | . |
| Gender |  |  |
| Male | 0.48 | 18222 |
| Female | 0.51 | 19562 |
| Other | 0.01 | 392 |
| Missing | 0.00 | 136 |
| Marital Status |  |  |
| Single/Never Been Married | 0.25 | 9431 |
| Married | 0.53 | 20360 |
| Separated | 0.02 | 727 |
| Divorced | 0.09 | 3636 |
| Widowed | 0.05 | 1978 |
| Domestic Partner | 0.05 | 1971 |
| Missing | 0.01 | 207 |
| Employment |  |  |
| Employed for an Employer | 0.51 | 19502 |
| Self-Employed | 0.09 | 3445 |
| Retired | 0.24 | 9016 |
| Student | 0.03 | 1144 |
| Homemaker | 0.05 | 2049 |
| Unemployed and Looking for a Job | 0.05 | 1777 |
| None of These/Other | 0.03 | 1292 |
| Missing | 0.00 | 87 |
| Education |  |  |
| Up to 8 Years | 0.01 | 210 |
| 9-15 Years | 0.66 | 25322 |
| 16+ Years | 0.33 | 12705 |
| Missing | 0.00 | 75 |
| Service Attendance |  |  |
| >1/Week | 0.07 | 2633 |
| 1/Week | 0.15 | 5887 |
| 1-3/Month | 0.07 | 2819 |
| A Few Times a Year | 0.23 | 8870 |
| Never | 0.47 | 17975 |
| Missing | 0.00 | 128 |
| Immigration Status |  |  |
| Born in This Country | 0.91 | 34865 |
| Born in Another Country | 0.08 | 3020 |
| Missing | 0.01 | 427 |
| Religion |  |  |
| Christianity | 0.60 | 22954 |
| Islam | 0.01 | 205 |
| Hinduism | 0.00 | 167 |
| Buddhism | 0.01 | 336 |
| Judaism | 0.02 | 638 |
| Sikhism | 0.00 | 24 |
| Baha'i | 0.00 | 13 |
| Jainism | 0.00 | 18 |
| Shinto | 0.00 | 12 |
| Taoism | 0.00 | 93 |
| Confucianism | 0.00 | 8 |
| Primal, Animist, or Folk Religion | 0.01 | 240 |
| Spiritism | . | . |
| African-Derived | . | . |
| Chinese | . | . |
| Some Other Religion | 0.03 | 1267 |
| No Religion/Atheist/Agnostic | 0.31 | 11870 |
| Missing | 0.01 | 467 |
| Race/Ethnicity |  |  |
| White | 0.62 | 23605 |
| Other | 0.03 | 997 |
| Black | 0.12 | 4501 |
| Asian | 0.06 | 2466 |
| Hispanic | 0.18 | 6724 |
| Other | . | . |
| Missing | 0.00 | 20 |

**Table 22b: Variations Across Demographic Characteristics (United_States)**

| Variable | Mean | SE | LCI | UCI | Global p-value |
| --- | --- | --- | --- | --- | --- |
| Age |  |  |  |  |  |
| 18-24 | 2.49 | 0.16 | 2.17 | 2.81 | 0.00 |
| 25-29 | 2.58 | 0.14 | 2.31 | 2.84 | . |
| 30-39 | 2.53 | 0.07 | 2.39 | 2.67 | . |
| 40-49 | 2.59 | 0.07 | 2.46 | 2.72 | . |
| 50-59 | 2.58 | 0.05 | 2.48 | 2.68 | . |
| 60-69 | 2.74 | 0.04 | 2.67 | 2.81 | . |
| 70-79 | 2.63 | 0.05 | 2.54 | 2.72 | . |
| 80 or Older | 2.16 | 0.08 | 2.00 | 2.32 | . |
| Gender |  |  |  |  |  |
| Male | 2.73 | 0.04 | 2.65 | 2.81 | 0.00 |
| Female | 2.44 | 0.04 | 2.37 | 2.50 | . |
| Other | 2.85 | 0.46 | 1.95 | 3.75 | . |
| Marital Status |  |  |  |  |  |
| Single/Never Been Married | 2.52 | 0.08 | 2.37 | 2.84 | 0.00 |
| Married | 2.67 | 0.03 | 2.61 | 2.27 | . |
| Separated | 2.70 | 0.34 | 2.03 | 3.37 | . |
| Divorced | 2.41 | 0.06 | 2.29 | 2.53 | . |
| Widowed | 2.27 | 0.08 | 2.12 | 2.41 | . |
| Domestic Partner | 2.61 | 0.12 | 2.37 | 2.84 | . |
| Employment |  |  |  |  |  |
| Employed for an Employer | 2.58 | 0.04 | 2.51 | 2.65 | 0.00 |
| Self-Employed | 3.01 | 0.11 | 2.79 | 3.22 | . |
| Retired | 2.63 | 0.04 | 2.56 | 2.70 | . |
| Student | 2.43 | 0.19 | 2.06 | 2.79 | . |
| Homemaker | 2.29 | 0.09 | 2.12 | 2.46 | . |
| Unemployed and Looking for a Job | 2.26 | 0.21 | 1.85 | 2.68 | . |
| None of These/Other | 2.16 | 0.22 | 1.73 | 2.60 | . |
| Education |  |  |  |  |  |
| Up to 8 Years | 1.50 | 0.56 | 0.35 | 2.66 | 0.00 |
| 9-15 Years | 2.43 | 0.04 | 2.35 | 2.51 | . |
| 16+ Years | 2.90 | 0.02 | 2.86 | 2.94 | . |
| Service Attendance |  |  |  |  |  |
| >1/Week | 2.62 | 0.09 | 2.45 | 2.79 | 0.106 |
| 1/Week | 2.68 | 0.06 | 2.56 | 2.80 | . |
| 1-3/Month | 2.74 | 0.09 | 2.57 | 2.91 | . |
| A Few Times a Year | 2.53 | 0.05 | 2.42 | 2.63 | . |
| Never | 2.55 | 0.04 | 2.46 | 2.63 | . |
| Immigration Status |  |  |  |  |  |
| Born in This Country | 2.57 | 0.03 | 2.51 | 2.62 | 0.106 |
| Born in Another Country | 2.76 | 0.12 | 2.52 | 2.99 | . |
| Religion |  |  |  |  |  |
| Christianity | 2.52 | 0.03 | 2.46 | 2.59 | 0.00 |
| Islam | 2.21 | 0.38 | 1.46 | 2.96 | . |
| Hinduism | 3.07 | 0.33 | 2.41 | 3.73 | . |
| Buddhism | 2.83 | 0.18 | 2.47 | 3.20 | . |
| Judaism | 2.65 | 0.15 | 2.36 | 2.94 | . |
| Sikhism | 3.60 | 0.97 | 1.47 | 5.73 | . |
| Baha'i | 2.04 | 0.53 | 0.92 | 3.16 | . |
| Jainism | 0.33 | 0.32 | -0.39 | 1.05 | . |
| Shinto | 4.87 | 1.20 | 2.17 | 7.56 | . |
| Taoism | 3.29 | 1.43 | -0.06 | 6.65 | . |
| Confucianism | 2.53 | 0.80 | 0.39 | 4.67 | . |
| Primal, Animist, or Folk Religion | 2.88 | 0.73 | 1.34 | 4.43 | . |
| Some Other Religion | 2.99 | 0.17 | 2.66 | 3.31 | . |
| No Religion/Atheist/Agnostic | 2.63 | 0.05 | 2.53 | 2.72 | . |
| Race/Ethnicity |  |  |  |  |  |
| White | 2.62 | 0.02 | 2.57 | 2.66 | 0.004 |
| Other | 2.78 | 0.11 | 2.57 | 2.99 | . |
| Black | 2.28 | 0.09 | 2.10 | 2.47 | . |
| Asian | 2.69 | 0.11 | 2.47 | 2.91 | . |
| Hispanic | 2.58 | 0.11 | 2.37 | 2.79 | . |
